# Hydroxyl Functionalized Pyridinium Ionic Liquids: Experimental and Theoretical Study on Physicochemical and Electrochemical Properties

**DOI:** 10.3389/fchem.2019.00625

**Published:** 2019-09-18

**Authors:** Kallidanthiyil Chellappan Lethesh, Sigvart Evjen, Jaganathan Joshua Raj, Denis C. D. Roux, Vishwesh Venkatraman, Kaushik Jayasayee, Anne Fiksdahl

**Affiliations:** ^1^Department of Chemistry, Norwegian University of Science and Technology, Trondheim, Norway; ^2^Center of Research in Ionic Liquids (CORIL), Universiti Teknologi PETRONAS, Perak, Malaysia; ^3^Université Grenoble Alpes (UGA), Grenoble, France; ^4^SINTEF Industry, Sustainable Energy Technology, Trondheim, Norway

**Keywords:** charge distribution, conductivity, COSMO-RS, electrodeposition, ionic liquid

## Abstract

Structurally modified hydroxyl functionalized pyridinium ionic liquids (ILs), liquid at room temperature, were synthesized and characterized. Alkylated *N*-(2-hydroxyethyl)-pyridinium ILs were prepared from alkylpyridines via corresponding bromide salts by *N*-alkylation (65–93%) and final anion exchange (75–96%). Pyridinium-alkylation strongly influenced the IL physicochemical and electrochemical properties. Experimental values for the ILs physicochemical properties (density, viscosity, conductivity, and thermal decomposition temperature), were in good agreement with corresponding predicted values obtained by theoretical calculations. The pyridinium ILs have electrochemical window of 3.0–5.4 V and were thermally stable up to 405°C. The IL viscosity and density were measured over a wide temperature range (25–80°C). Pyridine alkyl-substitution strongly affected the partial positive charge on the nitrogen atom of the pyridinium cations, as shown by charge distribution calculations. On-going studies on Mg complexes of the new ILs demonstrate promising properties for high current density electrodeposition of magnesium.

## Introduction

Ionic liquids (ILs) are organic salts with melting points preferably below 100°C (Welton, 1999). ILs have interesting properties, such as very low vapor pressure, large liquid range, wide electrochemical window, high thermal stability (Wasserscheid and Welton, 2008). Because of their unique properties, ILs are applied in various fields. One key advantage of ILs is the ability to tune their properties for specific applications by careful selection and design of cations and anions. For instance, some ILs are good electrolytes for rechargeable batteries and promising media for separation processes (Brennecke and Maginn, 2001; Domanska et al., 2007; Nasir Shah et al., 2014; Shah et al., 2016; Su et al., 2016). Others are promising solvents for chemical reactions and material transformations (Green et al., 2009; Chen and Ying, 2013). In order to develop processes and technologies, it is necessary to have a better understanding of the effect on IL physicochemical and electrochemical properties, obtained by chemical modifications of IL cations and anions.

**Figure 1:**
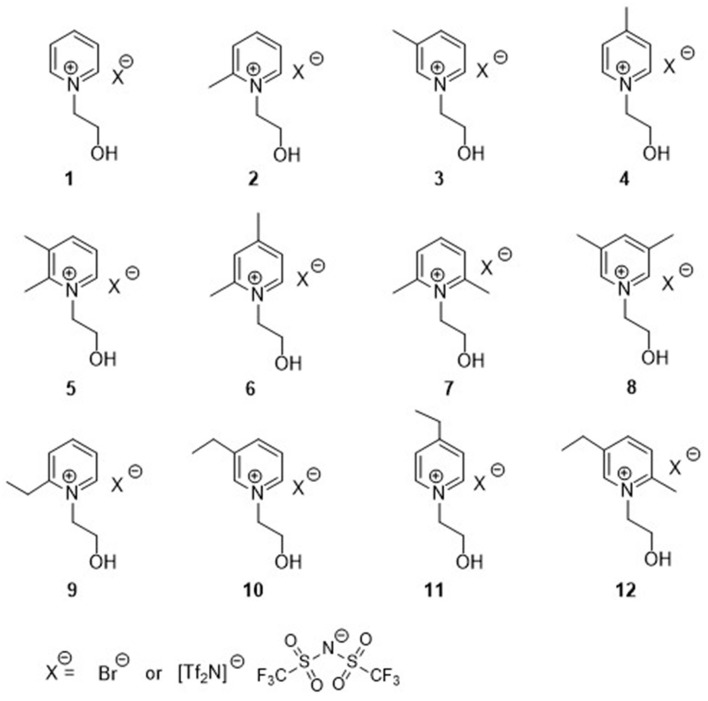
Structure of hydroxyl functionalized pyridinium ILs (**1-12Tf**_**2**_**N**) and the corresponding Br salts (**1-12Br**).

Although a large number of studies on the properties of conventional ILs has been performed (Suarez et al., 1998; Tokuda et al., 2005; Lethesh et al., 2012, 2014, 2015; Kermanioryani et al., 2016), functionalized ILs (Brasse et al., 2000; Visser et al., 2001; Lethesh et al., 2011, 2012; Leys et al., 2014; Raj et al., 2017) with coordinating group attached to the cation or anion or on both, are still to be explored (Davis, 2004; Ullah et al., 2015, 2016; Khan et al., 2018; Grøssereid et al., 2019). Functionalized ILs, especially with carboxylic groups are well-known for their ability to dissolve large amount of metal salts (Nockemann et al., 2008, 2009). However, most of them have very low electrochemical stability, in contrast to nitrile functionalized ILs having higher electrochemical stability. A comprehensive study on the physicochemical properties of nitrile-functionalized pyridinium ILs is reported (Lethesh et al., 2011), while limited studies on hydroxyl functionalized ILs are known. However, volumetric, physicochemical, as well as electrical conductivity properties of hydroxyl functionalized pyrrolidinium, piperidinum (Wu et al., 2011), and imidazolium (Liu et al., 2015) ILs have been studied. The synthesis of *N-*(hydroxyethyl) functionalized pyridinium ILs is also reported, but only unsubstituted pyridinium cations were studied (Makino et al., 2013). Selection of target compounds for IL synthesis have mostly been based on chemical experience and anticipated properties. In contrast, in this work, the strategy for design of target ILs for specific applications was based on initial theoretical predictions of IL thermophysical properties prior to experimental synthesis. Theoretical predictions focused mainly on the viscosity, density, and thermal stability of the ILs, as these properties are most important for IL applications. In the present project, the effect on IL physicochemical and electrochemical properties obtained by IL modification by varying the pyridinium cation structures, is studied (Figure 1). Hydroxyl functionalized ILs were modified by pyridine mono- or di-alkylation in different positions. An ethyl-C2 chain was used as a hydroxyl/pyridine spacer and bis(trifluoromethanesulfonyl) imide (Tf_2_N) was the anion. The temperature dependent density and viscosity, as well as the thermal stability of the hydroxyl functionalized pyridinium ILs were measured. The ILs were prepared for subsequent studies of their ability to give Mg-IL complexes by Mg coordination and their potential as electrolytes for Mg-ion rechargeable batteries.

## Experimental

### General

All commercially available chemicals were used without further purification. ^1^H and ^13^C NMR spectra were recorded using a 400 or 600 MHz spectrometer. Chemical shifts are reported in ppm (δ) downfield from tetramethylsilane (TMS) as internal standard. Coupling constants (*J*) are reported in Hertz (Hz). Accurate mass determination by HRMS in positive and negative mode was performed on a “Synapt G2-S” Q-TOF instrument from Water TM. Samples were ionized by ASAP (APCI) or ESI probes. No chromatographic separation was used prior to the mass analysis. Calculated exact mass and spectra processing was done by Waters TM Software Masslynx V4.1 SCN871.

### Synthesis and Characterization

#### General Procedure A; Synthesis of 1-(2-Hydroxyethyl)pyridinium Bromides (1-12Br)

2-Bromoethanol (e.g., 32 mmol) was added to a solution of the appropriate pyridine derivative (e.g., 32 mmol), and the reaction mixture was stirred at 50°C for 24 h. The obtained solid was washed with ethyl acetate (3 × 20 mL) and the residual solvent was removed in a rotary evaporator. The product was further dried in a Schlenk line at 50°C for 24 h to yield the respective pyridinium bromide salt (65–93%), which was characterized by ^1^H and ^13^C NMR (Supporting Information).

### 1-(2-Hydroxyethyl)pyridinium Bromide (1Br)

The target product was prepared by *General Procedure A* from pyridine (2.5 g, 32.20 mmol) and 2-bromoethanol (4.02 g, 32.20 mmol) and obtained as a white solid (11.60 g, 90%). ^1^H NMR [DMSO(d_6_)] δ: 3.85 (m, 2H), 4.7 (t, 2H, J = 5.40 Hz), 5.28 (t, 1H, J = 5.46 Hz), 8.15 (t, 1H, J = 8.19 Hz), 8.60 (t, 1H, J = 5.35 Hz), 9.0 (d, 2H, J = 5.46 Hz). ^13^C NMR [DMSO (d_6_)] δ: 60.47, 63.56, 128.18, 145.60, 146.04.

### 1-(2-Hydroxyethyl)-2-Methylpyridinium Bromide (2Br)

The target product was prepared by *General Procedure A* from 2-methylpyridine (3.0 g, 32.20 mmol) and 2-bromoethanol (4.02 g, 32.20 mmol) and obtained as colorless liquid (6 g, 86%). ^1^H NMR [DMSO(d_6_)] δ: 2.86 (m, 3H), 3.88 (m, 2H), 4.67(t, 2H, J = 5.11 Hz), 5.26 (t, 1H, J = 5.67 Hz), 7.97 (t, 1H, J = 6.13 Hz), 8.06 (d, 1H, J = 7.66 Hz), 8.48 (t, 1H, J = 6.13 Hz), 8.90 (d, 1H, J = 6.13 Hz). ^13^C NMR [DMSO (d_6_)] δ: 20.48, 59.72, 59.81, 125.56, 130.08, 145, 61, 146.57, 156.10.

### 1-(2-Hydroxyethyl)-3-Methylpyridinium Bromide (3Br)

The target product was prepared by *General Procedure A* from 3-methylpyridine (3.0 g, 32.20 mmol) and 2-bromoethanol (4.02 g, 32.20 mmol) and obtained as a colorless solid (6.0 g, 86%). ^1^H NMR [DMSO(d_6_)] δ: 2.48 (s, 3H), 3.86 (m, 2H), 4.61 (m, 2H, J = 5.20 Hz), 5.24 (t, 1H, J = 5.59 Hz), 8.06 (t, 1H, J = 6.64 Hz), 8.47 (d, 1H, J = 7.81 Hz), 8.84 (d, 1H, J = 5.86 Hz), 9.01 (s, 1H). ^13^C NMR [DMSO (d_6_)] δ: 18.31, 60.43, 63.47, 127.47, 138.79, 142.97, 144. 98, 146.32.

### 1-(2-Hydroxyethyl)-4-Methylpyridinium Bromide (4Br)

The target product was prepared by *General Procedure A* from 4-methylpyridine (5.0 g, 53.68 mmol) and 2-bromoethanol (6.81 g, 53.68 mmol) and obtained as a colorless solid (10.7 g, 91%). ^1^H NMR [DMSO(d_6_)] δ: 2.61(s, 3H), 3.82 (m, 2H), 4.59 (t, 2H, J = 5.21 Hz), 5.24 (t, 1H, J = 5.21 Hz), 7.99 (d, 2H, J = 5.95 Hz), 8.84 (d, 2H, J = 6.59 Hz). ^13^C NMR [DMSO (d_6_)] δ: 21.84, 60.44, 62.74, 128.44, 144, 60, 159. 31.

### 1-(2-Hydroxyethyl)-2,3-Dimethylpyridinium Bromide (5Br)

The target product was prepared by *General Procedure A* from 2,3-methylpyridine (5.0 g, 46.66 mmol) and 2-bromoethanol 5.92 g, 46.66 mmol) and obtained as a colorless solid (10.0 g, 92%). ^1^H NMR [DMSO (d_6_)] δ: 2.88 (s, 6H), 3.99 (m, 2H), 4.61 (t, 2H, J = 6.35 Hz), 4.73 (t, 1H, J = 5.19 Hz), 7.69 (t, 2H, J = 7.31 Hz), 8.19 (d, 1H, J = 7.74 Hz). ^13^C NMR [DMSO (d_6_)] δ: 22.15, 55.39, 58.89, 128.38, 144.78, 157.16.

### 1-(2-Hydroxyethyl)-2,5-Dimethylpyridinium Bromide (6Br)

The target product was prepared by *General Procedure A* from 2,5-methylpyridine (5.0 g, 46.66 mmol) and 2-bromoethanol (5.92 g, 46.66 mmol) and obtained as a colorless solid (9.9 g, 91%). ^1^H NMR [DMSO (d_6_)] δ: 2.48 (s, 3H), 2.73 (s, 3H), 3.92 (m, 2H), 4.62 (t, 1H, 5.81 Hz), 4.74 (t, 2H, 5.77 Hz), 7.64 (d, 1H, J = 7.03 Hz), 7.69 (s, 1H), 8.68 (d, 1H, J = 6.44 Hz). ^13^C NMR [DMSO (d_6_)] δ: 17.29, 19.80, 59.77, 60.97, 117.87, 124.51, 139.58, 144.

### 1-(2-Hydroxyethyl)-2,6-Dimethylpyridinium Bromide (7Br)

The target product was prepared by *General Procedure A* from 2,6-methylpyridine (5.0 g, 46.66 mmol) and 2-bromoethanol (5.92 g, 46.66 mmol) and obtained as a colorless solid (7.0 g, 65%). ^1^H NMR [DMSO (d_6_)] δ: 2.81 (s, 6H), 3.82 (t, 2H, J = 5.51 Hz), 4.59 (t, 2H, J = 5.38 Hz), 7.83 (d, 2H, J = 7.85 Hz), 8.28 (t, 2H, J = 7.85 Hz). ^13^C NMR [DMSO (d_6_)] δ: 21.86, 54.94, 58.95, 128.07, 144.79, 156.74.

### 1-(2-Hydroxyethyl)-3,5-Dimethylpyridinium Bromide (8Br)

The target product was prepared by *General Procedure A* from 3,5-methylpyridine (5.0 g, 46.66 mmol) and 2-bromoethanol (5.92 g, 46.66 mmol) and obtained as a colorless solid (10.0 g, 92%). ^1^H NMR [DMSO (d_6_)] δ: 2.88 (s, 6H), 3.87 (m, 2H), 4.57 (t, 2H, J = 4.57 Hz), 5.23 (t, 1H, J = 5.08 Hz), 8.33 (s, 1H), 8.80 (s, 1H). ^13^C NMR [DMSO (d_6_)] δ: 18.16, 60.37, 63.32,137.94, 142.41, 146.71.

### 1-(2-Hydroxyethyl)-2-Ethylpyridinium Bromide (9Br)

The target product was prepared by *General Procedure A* from 2-ethylpyridine (5.0 g, 46.66 mmol) and 2-bromoethanol (5.92 g, 46.66 mmol) and obtained as a colorless solid (7.2 g, 66%). ^1^H NMR [DMSO (d_6_)] δ: 1.33 (t, 3H, J = 7.74 Hz), 3.20 (m, 2H), 3.87 (m, 2H), 4.68 (t, 2H, J = 4.30 Hz), 5.27 (t, 1H, J = 5. 08 Hz), 7.98 (t, 1H, J = 6.17 Hz), 8.06 (d, 1H, J = 7.20 Hz), 8.52 (m, 1H, J = 6.48 Hz), 8.90 (m, 1H, J = 5.08 Hz). ^13^C NMR [DMSO (d_6_)] δ:12.61, 25.70, 59.26, 60.16, 125.43, 128.07, 145.85, 146.65, 160.16.

### 1-(2-Hydroxyethyl)-3-Ethylpyridinium Bromide (10Br)

The target product was prepared by *General Procedure A* from 3-ethylpyridine (5.0 g, 46.66 mmol) and 2-bromoethanol (5.92 g, 46.66 mmol) and obtained as a colorless solid (10.15 g, 93%). ^1^H NMR [DMSO (d_6_)] δ: 1.31 (t, 3H, J = 7.79 Hz), 2.88 (m, 2H), 3.96 (m, 2H), 4. 73(t, 3H, J = 5.45 Hz), 7.93 (t, 1H, J = 7.30 Hz), 8.36 (d, 1H, J = 7.91 Hz), 8.77 (d, 2H, J = 6.09 Hz), 8.87 (s, 1H). ^13^C NMR [DMSO (d_6_)] δ: 14.37, 26.21, 60.95, 64.01, 127.96, 143.26, 144.98, 145.52, 145.68.

### 1-(2-Hydroxyethyl)-4-Ethylpyridinium Bromide (11Br)

The target product was prepared by *General Procedure A* from 4-ethylpyridine (5.0 g, 46.66 mmol) and 2-bromoethanol (5.92 g, 46.66 mmol) and obtained as a colorless solid (10.0 g, 92%). ^1^H NMR [DMSO (d_6_)] δ: 1.21 (t, 3H, J = 7.18 Hz), 2.87 (m, 2H), 3.79 (t, 2H, J = 5.03 Hz), 4.57 (t, 2H, J = 5.06 Hz), 5.18 (s, 1H), 7.97 (d, 2H, J = 6.66 Hz), 8.84 (d, 2H, J = 6.98 Hz). ^13^C NMR [DMSO (d_6_)] δ: 13.96, 28.44, 60.45, 62.70, 127.28, 144.90, 164.23.

### 5-Ehyl-1-(2-Hydroxyethyl)-2-Methylpyridinium Bromide (12Br)

The target product was prepared by *General Procedure A* from 5-ethyl-2-methylpyridine (5.0 g, 41.26 mmol) and 2-bromoethanol (5.23 g, 41.26 mmol) and obtained as a colorless solid (9.5 g, 93%). ^1^H NMR [DMSO (d_6_)] δ: 1.20 (t, 3H, J = 7.48 Hz), 2.71 (t, 2H, J = 7.48 Hz), 2.75 (s, 3H), 3.81 (t, 2H), 4.56 (t, 2H, J = 5. 34 Hz), 5.19 (t, 1H), 7.92(d, 1H, J = 8.30 Hz), 8.33 (t, 1H, 6.30 Hz), 8.79 (s,1H). ^13^C NMR [DMSO (d_6_)] δ: 14.86, 20.04, 25.07, 59.59, 59.97, 129.61, 141.50, 145.16, 153.47.

### *General Procedure B*; Preparation of 1-(2-Hydroxyethyl)pyridinium ILs (1-12Tf_2_N)

To a solution of the appropriate bromide (**1-12Br**) (e.g., 14.70 mmol) in water (15 mL), lithiumbis(trifluoromethylsulfonyl)imide (4.22 g, 14.70 mmol) was added. The mixture was stirred at room temperature for 2 h and two layers were formed. The aqueous layer was removed by decantation and the ionic liquid layer was washed several times with deionized water until the aqueous layer gave no precipitate by treatment with a AgNO_3_ solution. The water was removed by a rotary evaporator and the IL was further dried in a Schlenk line at 50°C for 24 h. The target hydroxyl functionalized pyridinium IL (75–96%) was characterized by ^1^H and ^13^C NMR (Supporting Information). Accurate high-resolution mass determination by HRMS in positive and negative mode, confirmed the identity of the respective pyridinium cation and the Tf_2_N imide anion.

### 1-(2-Hydroxyethyl)pyridinium bis(Trifluoromethanesulfonyl)imide (1Tf_2_N)

The target product, prepared by *General Procedure B* from **1Br** (3.0 g, 14.70 mmol) and LiTf_2_N (4.22 g, 14.70 mmol), was obtained as a colorless liquid (5.5 g, 92%). ^1^H NMR [DMSO (d_6_)] δ: 8.99 (d, 2H, J = 5.74 Hz), 8.60 (t, 1H, J = 6.45 Hz), 8.14 (t, 2H, J = 6.81 Hz), 5.22 (s, 1H), 4.65 (t, 2H, J = 4.74 Hz), 3.87 (t, 2H, J = 5.15 Hz). ^13^C NMR [DMSO (d_6_)] δ:145.99, 145.56, 128.15, 121.52(q), 63.66, 60.46. HRMS (ES+): calcd for C_7_H_10_NO [M] 124.0762, obsd 124.0760. HRMS (ES-): calcd for Tf_2_N [M] 279.9173, obsd 271.9173.

### 1-(2-Hydroxyethyl)-2-Methylpyridinium bis(Trifluoromethanesulfonyl)imide (2Tf_2_N)

The target product, prepared by *General Procedure B* from **2Br** (2.5 g, 11.46 mmol) and LiTf_2_N (3.29 g, 11.46 mmol), was obtained as a colorless liquid **(**4.5 g, 93%). ^1^H NMR [DMSO (d_6_)] δ: 8.86 (d, 1H, J = 5.42 Hz), 8.46 (t, 1H, J = 6.77 Hz), 8.04 (d, 1H, J = 8.13 Hz), 7.97 (t, 1H, J = 6.32 Hz), 5.29 (s, 1H), 4.64 (t, 2H, J = 5.67 Hz), 3.89 (t, 2H, J = 4.90 Hz), 2.85 (s, 3H). ^13^C NMR [DMSO (d_6_)] δ:156.06, 146.51, 145.55, 130.06, 125.53, 121.52 (q), 59.80, 59.73, 20.40. HRMS (ES+): calcd for C_8_H_12_NO [M] 138.0919, obsd 138.0917. HRMS (ES-): calcd for Tf_2_N [M] 279.9173, obsd 271.9179.

### 1-(2-Hydroxyethyl)-3-Methylpyridinium bis(Trifluoromethanesulfonyl)imide (3Tf_2_N)

The target product, prepared by *General Procedure B* from **3Br** (2.5 g, 11.46 mmol) and LiTf_2_N (3.29 g, 11.46 mmol), was obtained as a colorless liquid (4.0 g, 83%). ^1^H NMR [DMSO (d_6_)] δ: 8.89 (s, 1H), 8.82 (d, 1H J = 6.08 Hz), 8.45 (d, 1H, J = 7.91 Hz), 8.05 (t, 1H, J = 6.08 Hz), 5.26 (s, 1H), 4.61 (t, 2H, J = 5.09 Hz), 3.88 (t, 2H, J = 5. Hz), 2.51 (s, 3H). ^13^C NMR [DMSO (d_6_)] δ: 146.27, 144.89, 142.88, 127.42, 121.52 (q), 63.55, 60.42, 18.20. HRMS (ES+): calcd for C_8_H_12_NO [M] 138.0919, obsd 138.0918. HRMS (ES-): calcd for Tf_2_N [M] 279.9173, obsd 279.9179.

### 1-(2-Hydroxyethyl)-4-Methylpyridinium bis(Trifluoromethanesulfonyl)imide (4Tf_2_N)

The target product, prepared by *General Procedure B* from **4Br** (5.0 g, 22.92 mmol) and LiTf_2_N (6.58, 22.92 mmol), was obtained as a colorless liquid (8.0 g, 83%). ^1^H NMR [DMSO (d_6_)] δ: 8.50 (d, 2H, J = 6.03 Hz), 7.83 (d, 2H, J = 6.30 Hz), 4.52 (t, 2H, J = 4.79 Hz), 3.91 (t, 2H, J = 4.79 Hz), 3.31 (s, 1H), 2.63 (s, 3H). ^13^C NMR [DMSO (d_6_)] δ: 144.42, 128.97, 117.97, 121.52 (q), 63.49, 60.85, 21.75. HRMS (ES+): calcd for C_8_H_12_NO [M] 138.0919, obsd 138.0918. HRMS (ES-): calcd for Tf_2_N [M] 279.9173, obsd 279.9173.

### 1-(2-Hydroxyethyl)-2,3-Dimethylpyridinium bis(Trifluoromethanesulfonyl)imide (5Tf_2_N)

The target product, prepared by *General Procedure B* from **5Br** (5.0 g, 21.54 mmol) and LiTf_2_N (6.18, 21.54 mmol), was obtained as a colorless liquid (9.0 g, 96%). ^1^H NMR [DMSO (d_6_)] δ: 8.24 (t, 1H, J = 7.50 Hz), 7.80 (d, 2H, J = 8.03 Hz), 5.24 (s, 1H), 4.57 (t, 2H, J = 5.34 Hz), 3.81 (d, 2H, J = 5.57 Hz), 2.79 (s, 6H). ^13^C NMR [DMSO (d_6_)] δ: 156.74, 144.79, 128.05, 121.54 (q), 59.03, 54.87, 21.77. HRMS (ES+): calcd for C_9_H_14_NO [M] 152.1075, obsd 152.1071. HRMS (ES-): calcd for Tf_2_N [M] 279.9173, obsd 279.9176.

### 1-(2-Hydroxyethyl)-2,5-Dimethylpyridinium bis(Trifluoromethanesulfonyl)imide (6Tf_2_N)

The target product, prepared by *General Procedure B* from **6Br** (5.0 g, 21.54 mmol) and LiTf_2_N (6.18, 21.54 mmol), was obtained as a colorless liquid (7.6 g, (81%). ^1^H NMR [DMSO (d_6_)] δ: 8.67 (d, 1H, J = 6.20 Hz), 7.84 (s, 1H), 7. 68 (d, 1H, J = 4.55 Hz), 5.30 (s, 1H), 4.56 (t, 2H, J = 5.16 Hz), 3.84 (t, 2H, J = 4.95 Hz), 2.74 (s, 3H), 2.49 (s, 3H). ^13^C NMR [DMSO (d_6_)] δ: 158.74, 154.70, 145.43, 130.17, 126.06, 121.52(q), 59.83, 58.90, 21.45, 19.88. HRMS (ES+): calcd for C_9_H_14_NO [M] 152.1075, obsd 152.1077. HRMS (ES-): calcd for Tf_2_N [M] 279.9173, obsd 279.9177.

### 1-(2-Hydroxyethyl)-2,6-Dimethylpyridinium bis(Trifluoromethanesulfonyl)imide (7Tf_2_N)

The target product, prepared by *General Procedure B* from **7Br** (5.0 g, 21.54 mmol) and LiTf_2_N (6.18, 21.54 mmol), was obtained as a colorless liquid (7.7 g, (82%). ^1^H NMR [DMSO (d_6_)] δ: 8.26 (t, 2H, J = 7.61 Hz), 7.80 (d, 1H, J = 7.61 Hz), 5.24 (s, 1H), 4.57 (t, 2H, J = 5.74 Hz), 3.81 (t, 2H, J = 5.74 Hz), 2.79 (s, 6H). ^13^C NMR [DMSO (d_6_)] δ:156.74, 144.79, 128.05, 121.50 (q), 59.03, 54.87, 21.77. HRMS (ES+): calcd for C_9_H_14_NO [M] 152.1075, obsd 152.1074. HRMS (ES-): calcd for Tf_2_N [M] 279.9173, obsd 279.9177.

### 1-(2-Hydroxyethyl)-3,5-Dimethylpyridinium bis(Trifluoromethanesulfonyl)imide (8Tf_2_N)

The target product, prepared by *General Procedure B* from **8Br** (5.0 g, 21.54 mmol) and LiTf_2_N (6.18, 21.54 mmol), was obtained as a colorless liquid (7.7 g, 82%). ^1^H NMR [DMSO (d_6_)] δ: 8.74 (s, 2H), 8.30 (s, 1H), 5.22 (s, 1H), 4.54 (t, 2H, J = 5.10 Hz), 3.87 (t, 2H, J = 4.54 Hz), 2.47 (s, 6H). ^13^C NMR [DMSO (d_6_)] δ:146.69, 142.36, 138.0, 121.50 (q), 63.44, 60.39, 18.07. HRMS (ES+): calcd for C_9_H_14_NO [M] 152.1075, obsd 152.1075. HRMS (ES-): calcd for Tf_2_N [M] 279.9173, obsd 279.9178.

### 2-Ethyl-1-(2-Hydroxyethyl)pyridinium bis(Trifluoromethanesulfonyl)imide (9Tf_2_N)

The target product, prepared by *General Procedure B* from **9Br** (6.0 g, 25.84 mmol) and LiTf_2_N (7.42 g, 25.84 mmol), was obtained as a colorless liquid (8.5 g, 76%). ^1^H NMR [DMSO (d_6_)] δ:8.82 (d, 1H, J = 5.03 Hz), 8.43 (t, 1H, J = 6.82 Hz), 7.95 (d, 1H, J = 8.42 Hz), 7.88 (t, 1H, J = 6.82 Hz), 5.30 (s, 1H), 4.60 (t, 2H, J = 5.47 Hz), 3.80 (t, 2H, J = 5.47 Hz), 3.12 (m, 2H), 1.27 (t, 3H, J = 7.30 Hz). ^13^C NMR [DMSO (d_6_)] δ:160.17, 146.67, 145.81, 128. 04, 125.40, 121.54(q), 60.17, 59.29, 25.66, 12.50. HRMS (ES+): calcd for C_9_H_14_NO [M] 152.1075, obsd 152.1073. HRMS (ES-): calcd for Tf_2_N [M] 279.9173, obsd 279.9179.

### 3-Ethyl-1-(2-Hydroxyethyl)pyridinium bis(Trifluoromethanesulfonyl)imide (10Tf_2_N)

The target product, prepared by *General Procedure B* from **10Br** (5.0 g, 21.54 mmol) and LiTf_2_N (6.18 g, 21.54 mmol), was obtained as a colorless liquid (8.5 g, 91%). ^1^H NMR [DMSO (d_6_)] δ: 8.88 (s, 1H), 8.80 (d, 1H, J = 6.21 Hz), 8.49 (d, 2H, J = 7.99 Hz), 8.05 (t, 1H, J = 6.24 Hz), 5.20 (s, 1H), 4.60 (t, 2H, J = 4.55 Hz), 3.88 (t, 2H, J = 5.91 Hz), 2.84 (m, 2H), 1.28 (t, 3H, J = 6.98 Hz). ^13^C NMR [DMSO (d_6_)] δ:145.28, 144.51, 144.33, 143.04, 127.63, 121.50 (q), 63.57, 60.43, 25.52, 14.63. HRMS (ES+): calcd for C_9_H_14_NO [M] 152.1075, obsd 152.1073. HRMS (ES-): calcd for Tf_2_N [M] 279.9173, obsd 279.9178.

### 4-Ethyl-1-(2-Hydroxyethyl)pyridinium bis(Trifluoromethanesulfonyl)imide (11Tf_2_N)

The target product, prepared by *General Procedure B* from **11Br** (5.0 g, 21.54 mmol) and LiTf_2_N (6.18 g, 21.54 mmol), was obtained as a colorless liquid (7.0 g, 75%). ^1^H NMR [DMSO (d_6_)] δ: 8.80 (d, 2H, J = 6.97 Hz), 7.96 (d, 2H, J = 6.39 Hz), 5.18 (s, 1H), 4.52 (t, 2H, J = 5.25 Hz), 3.78 (t, 2H, J = 4.59 Hz), 2.86 (m, 2H), 1.21 (t, 3H, J = 7.21 Hz). ^13^C NMR [DMSO (d_6_)] δ:164.29, 144.87, 127.27, 121.54 (q), 62.81, 60.44, 28.44, 13.87. HRMS (ES+): calcd for C_9_H_14_NO [M] 152.1075, obsd 152.1078. HRMS (ES-): calcd for Tf_2_N [M] 279.9173, obsd 279.9178.

### 5-Ethyl-1-(2-Hydroxyethyl)-2-Methylpyridinium bis(Trifluoromethanesulfonyl)imide (12Tf_2_N)

The target product, prepared by *General Procedure B* from **12Br** (5.0 g, 20.31 mmol) and LiTf_2_N (5.83 g, 20.31 mmol), was obtained as a colorless liquid (8.1 g, 89%). ^1^H NMR [DMSO (d_6_)] δ:8.43 (s, 1H), 8.23 (m, 1H, J = 6.19 Hz), 7.79 (d, 1H, J = 8.47 Hz), 4.54 (t, 2H, J = 4.79 Hz), 3.95 (t, 2H, J = 4.44 Hz), 3.29 (s, 1H), 2.82(m, 2H), 2.76(s, 3H), 1.27 (t, 3H, J = 7.61 Hz). ^13^C NMR [DMSO (d_6_)] δ:145.62, 145.08, 142.72, 130.12, 121.4(q), 117.94, 60.36, 60.08, 25.53, 20.05, 14.25. HRMS (ES+): calcd for C_10_H_16_NO [M] 166.1232, obsd 166.1231. HRMS (ES-): calcd for Tf_2_N [M] 279.9173, obsd 279.9178.

### Physical Properties

Methods for measurement of IL viscosity, density, melting points (T_m_), glass transition (T_g_) temperatures, thermal decomposition (TGA) are described in Supporting Information.

### Electrochemical Stability Measurement

The ILs were dried in a Schlenk line at 55°C for 24 h before electrochemical studies. The measurement of electrochemical window (EW) was carried using Multichannel EIS System (Model: WEIS510), WonATech Co.Ltd in a glove box (25°C, water and oxygen content <1 ppm). Generally, three electrode systems were used in this EW measurement, namely the working electrode (GC-macro electrode), a counter electrode (platinum wire) and a reference electrode (Ag/AgCl electrode with glass frit). Approximately 0.5 mL of ILs sample was loaded into the glass cell for each experiment. Cyclic voltammetry (CV) measurement was taken with the sweep rate of 10 mV/s, starting from the anodic to the cathodic potentials and then reversing back to the initial value. In this case, 0.5 mA/cm^2^ was selected as the cut off current density, based on previously published work and for easy comparison (Rogers et al., 2009; Hayyan et al., 2013).

### Computational Details

For the optimization of the cation and anion structures, the BP functional B88-p86 with a triple-ζ valence polarized basis set (TZVP) and the resolution of identity standard (RI) approximation was employed (Weigend and Ahlrichs, 2005). The calculations were carried out using the COSMO-RS (COnductor-like Screening MOdel for Real Solvent) (Klamt, 2005) solvation scheme implemented in the software ORCA (Klamt, 2005). The output from ORCA (COSMO files) were then used as an input to the software COSMOtherm along with the parameterization set BP_TZVP_C30_01601. From this, values for ILs properties, such as temperature dependent densities and viscosities were obtained. The values for T_*d*_, T_*g*_, and electrical conductivity were estimated using machine learning models, reported in previous studies from our group (Venkatraman and Alsberg, 2016; Venkatraman et al., 2018a,b).

## Results and Discussion

### Synthesis

**Scheme 1:**
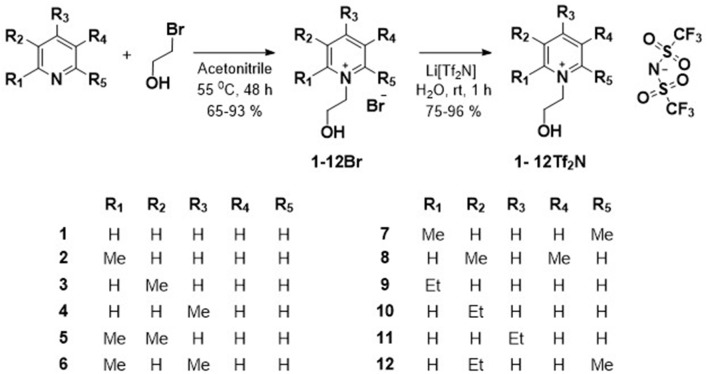
Synthesis of hydroxyl functionalized pyridinium ILs (**1-12Tf**_**2**_**N**).

The syntheses of hydroxyl functionalized pyridinium ILs (**1-12Tf**_**2**_**N**) were performed in two steps (Scheme 1). Introduction of the *N*-(hydroxyethyl)-functionality on pyridine was carried out by *N*-alkylation of the appropriate methyl-/ethyl-substituted pyridines with 2-bromoethanol. The resulting *N*-(hydroxyethyl)-pyridinium bromide salts (**1-12Br**; (65–93%) were solids at room temperature. Subsequent anion exchange, by treatment of the pyridinium bromide salts (**1-12Br**) with lithium bis(trifluromethylsulfonyl)imide (LiTf_2_N), afforded the corresponding Tf_2_N based *N*-(hydroxyethyl)-pyridinium ILs (**1-12Tf**_**2**_**N**; 75–96%), being liquid at room temperature. The molecular structure of the synthesized ILs were confirmed by ^1^H, ^13^C NMR, and HRMS (in positive and negative mode for confirmation of the respective cations and the imide anion).

### Thermal Profile

**Figure 2:**
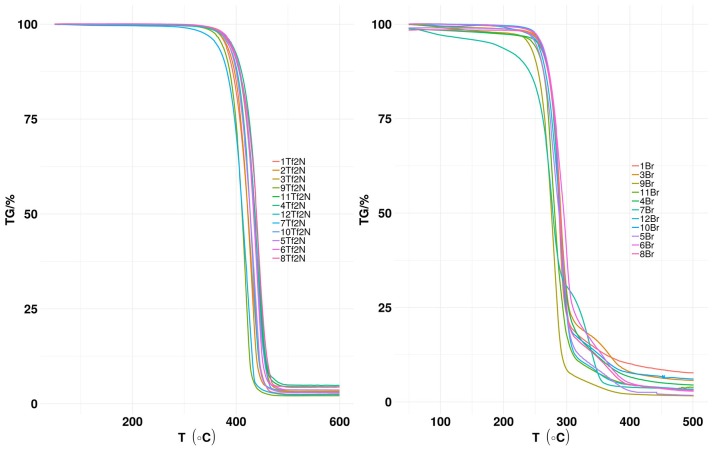
TGA profile of (Tf_2_N) anion-based hydroxyl functionalized pyridinium ILs (**1-12Tf**_**2**_**N**) and the corresponding Br salts (**1-12Br**).

The thermal stability of (Tf_2_N) based ILs (**1-12Tf**_**2**_**N**) and the corresponding bromide salts (**1-12Br** was measured (Figure 2).

The thermal stability (T_*d*_) of the hydroxyl functionalized bromide salts **1-12Br** was in the range of 234–269°C (Table 2 in Supporting Information). Pyridinium alkyl-substitution seems to have minor influence on the thermal stability, as demonstrated by the identical T_*d*_ value (267°C) for both **10Br** (3-ethyl) and non-substituted **1Br**. This result is in accordance with previous reports for *N*-alkylated pyridinium bromide salts, as similar T_*d*_ values of N-ethylpyridinium bromide (T_*d*_ 230°C) and 2,3-dimethyl-derivate (T_*d*_ 234°C) are reported (Venkatraman et al., 2018a).

T_*d*_ values of [Tf_2_N] anion based ILs **1-12Tf**_**2**_**N** (Figure 2 above and Table 1 in Supporting Information) are significantly higher (377–405°C) than the bromide salts **1-12Br** because of the weakly coordinating nature of the Tf_2_N anion. Like the bromide salts, pyridine alkyl-substitution has minor influence on thermal stability of [Tf_2_N] anion based ILs. For example, the T_*d*_ values of the non-alkylated pyridinium IL (**1Tf**_**2**_**N**; T_*d*_ 389°C), the mono-methyl IL **2Tf**_**2**_**N** (T_*d*_ 392°C), the di-methyl IL **5Tf**_**2**_**N** (T_*d*_ 377°C), the mono-ethyl IL **9Tf**_**2**_**N** (T_*d*_ 384°C) as well as ethyl-methyl IL **12Tf**_**2**_**N** (T_*d*_ 397°C) are quite similar. The studies show that hydroxyl functionalized (Tf_2_N) based ILs are thermally more stable than their nitrile functionalized analog, as shown by the higher T_*d*_ value (389°C) of **1Tf**_**2**_**N** than the corresponding nitrile functionalized IL (360°C) at similar experimental conditions (Lethesh et al., 2011).

Predicted values for thermal stability of the ILs were obtained using machine learning models (Tables 1, 2 in Supporting Information) (Venkatraman et al., 2018a). The deviation of the values of **5Tf**_**2**_**N** varies with only 3°C (experimental T_*d*_ 377°C; predicted T_*d*_ 374°C), while the largest deviation (39°C) was observed for **7Tf**_**2**_**N** (experimental T_*d*_ 403°C; predicted T_*d*_ 364°C). In most cases, deviations between the experimental and calculated T_*d*_ values of **1-12Tf**_**2**_**N** ILs are within the calculated prediction uncertainties (±60°C).

Glass transition temperatures were measured for the ILs (**1-12Tf**_**2**_**N**; Table 1). The lowest and highest values were observed for **10Tf**_**2**_**N** (T_*g*_ −77.4°C) and **5Tf**_**2**_**N** (T_*g*_ −65.5°C), respectively. No systematic effect on T_*g*_ was observed by varied pyridinium substitution of the hydroxyl functionalized ILs **1-12Tf**_**2**_**N**. For instance, the T_*g*_ values of non-substituted **1Tf**_**2**_**N** (T_*g*_ −72.9°C) and methyl-ethyl derivate **12Tf**_**2**_**N** (T_*g*_ −72.8) are close. T_*g*_ of the hydroxyl functionalized ILs (**1-12Tf**_**2**_**N**) are significantly lower than the analogous non-hydroxy ILs; as demonstrated by the higher value for the N-ethylpyridinium Tf_2_N IL (T_*g*_ −39.91°C) (Bittner et al., 2012) relative to the *N*-hydroxyethyl functionalized analog, **1Tf**_**2**_**N** (T_*g*_ −72.9°C) (Liu et al., 2010).

**Table 1:** Glass transition temperature (T_*g*_) of [Tf_2_N] based hydroxyl functionalized ILs (1-12Tf_2_N).

**Entry**	**ILs**	**T_g_ (^°^C)**	**ILs**	**T_g_ (^°^C)**
1	1Tf_2_N	−72.9	7Tf_2_N	−71.1
2	2Tf_2_N	−73.8	8Tf_2_N	−69.8
3	3Tf_2_N	−75.5	9Tf_2_N	−70.7
4	4Tf_2_N	−74.3	10Tf_2_N	−77.4
5	5Tf_2_N	−65.5	11Tf_2_N	−76.3
6	6Tf_2_N	−67.8	12Tf_2_N	−72.8

### Density

**Figure 3:**
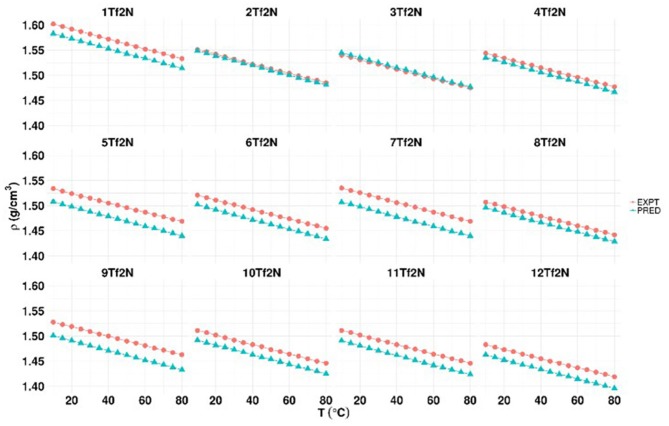
Experimental and predicted density of [Tf_2_N] ionic liquids (**1-12Tf**_**2**_**N**).

The density (ρ) of ILs **1-12Tf**_**2**_**N** was measured at 15 different temperatures from 10 to 80°C (Figure 3 and Table 3 in Supporting Information). The density of **1Tf**_**2**_**N** was in accordance with the reported value (Makino et al., 2013) at similar experimental conditions.

The density dropped considerably by increased alkyl substitution. This is seen by the reduction in density going from the highest density value for non-alkylated pyridinium IL (**1Tf**_**2**_**N**, 1.602 g/cm^3^) to the 2-methyl (**2Tf**_**2**_**N**; 1.551 g/cm^3^), 3-ethyl (**10 Tf**_**2**_**N**, 1.511 g/cm^3^), and 2-ethyl-6-ethyl (**12 Tf**_**2**_**N**;1.483 g/cm^3^) compounds. This effect may be due to the larger dispersive forces afforded by the increased number of carbons on the cation (GaciñO et al., 2011). The position of methyl substituents slightly influenced the density of the methyl-pyridinium ILs, as shown by the similar density for the 2-methyl (**2Tf**_**2**_**N**), 3-methyl (**3Tf**_**2**_**N**), and 4-methyl (**4Tf**_**2**_**N**) isomers.

The density of dimethyl substituted ILs (**5-8Tf**_**2**_**N**) increased significantly going from 3,5-dimethyl IL **8Tf**_**2**_**N** (1.507 g/cm^3^) through the 2,4-methyl-isomer **6Tf**_**2**_**N** (1.521 g/cm^3^) to the 2,3- and 2,6-dimethyl ILs **5Tf**_**2**_**N** and **7Tf**_**2**_**N** (1.535 g/cm^3^). The *N*-hydroxyethyl functionalized ILs **1-12Tf**_**2**_**N** have significantly higher density compared with their *N*-alkyl analog (Papaiconomou et al., 2012; Verdía et al., 2014). For example, the density of **1Tf**_**2**_**N** (1.592 g/cm^3^ at 20°C) is significantly higher than the density of *N*-ethylpyridinium Tf_2_N IL (1.534 g/cm^3^). Similarly, 1-(2-cyanoethyl)pyridinium IL also showed significantly lower density (1.50 g/cm^3^) compared to the hydroxyl functionalized analog studied in this work (Lethesh et al., 2011).

Temperature dependent densities of the ILs were obtained by DFT/COSMO-RS calculations (Figure 3 and Table 4 in Supporting Information). The smallest and highest deviations were observed for **2Tf**_**2**_**N/3Tf**_**2**_**N/4Tf**_**2**_**N/8Tf**_**2**_**N** and **5Tf**_**2**_**N/7Tf**_**2**_**N/9Tf**_**2**_**N**, respectively. The experimental and predicted density values of the ILs were in good agreement, as the average absolute deviation between the experimental and predicted density values was ≈ 0.017 g/cm^3^. IL **2Tf**_**2**_**N** (0.002 g/cm^3^) and **7Tf**_**2**_**N** (0.028 g/cm^3^) had the lowest and highest deviations (at 10°C).

### Viscosity

**Figure 4:**
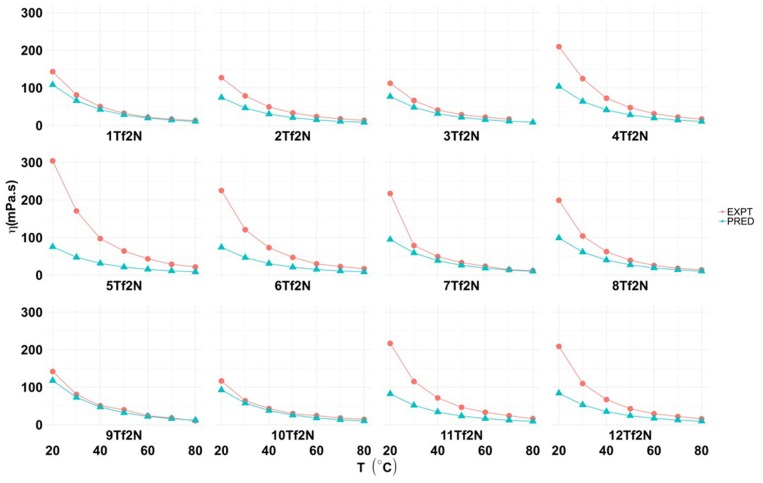
Experimental and predicted values for viscosity (η; mPa.s) of hydroxyl functionalized pyridinium ILs (**1-12Tf**_**2**_**N**).

As expected, the viscosity (η) of ILs **1-12Tf**_**2**_**N**, measured at seven temperatures (20, 30, 40, 50, 60, 70, 80°C) decreased by increasing temperature (Figure 4 and Table 5 in Supporting Information).

The viscosity of **1Tf**_**2**_**N** (143.1 mPa s) at 20°C was in accordance with previously reports (145.7 mPa s) (Makino et al., 2013). The study shows that the position of methyl/ethyl pyridine-substitution influences the viscosity of hydroxyl functionalized pyridinium ILs. Introduction of a methyl group in the pyridine 2- and 3-positions reduces the viscosity from 143.1 mPa.s (**1Tf**_**2**_**N**) to, respectively, 127.10 mPa.s (2-methyl; **2Tf**_**2**_**N**) and to the minimum value of 112.20 mPa.s (3-methyl; **3Tf**_**2**_**N**). This effect may be due to the asymmetry of the 2- and 3-methylpyridinium cations. The viscosity increased significantly for the 4-methyl compound **4Tf**_**2**_**N** (209.70 mPa.s; Figure 4). Similar observations are reported for other functionalized pyridinium ILs, such as the *N*-(3-cyanopropyl)pyridinium [Tf_2_N] IL (363 mPa.s). By methyl-pyridinium substituents in 2-, 3- and 4-positions, the viscosity changes to 308, 163, and 325 mPa.s, respectively (Lethesh et al., 2011). Also the ethyl substituted analogs ILs followed the same trend; 2-ethyl; **9Tf**_**2**_**N** (141.7 mPa.s); 3-ethyl; **10Tf**_**2**_**N** (116.6 mPa.s) and 4-ethyl IL **11Tf**_**2**_**N** (216 mPa.s). The viscosity of N-ethylpyridinium Tf_2_N IL is almost tree time lower (50.27 mPa.s) than the *N*-hydroxyethyl functionalized ILs (**1-12Tf**_**2**_**N**) (González et al., 2015).

Predicted values for temperature dependent viscosity of the ILs were obtained by DFT/COSMO-RS calculations (Figure 4 and Table 6 in Supporting Information). The theoretically predicted viscosity values were always lower than the experimental values. The deviation between the experimental and predicted viscosity values were very low at high temperatures (80°C; e.g., <2 mPa.s for **5Tf**_**2**_**N**). However, large differences were observed (25–228 mPa.s) at low temperature (20°C).

The viscosity of *N-(*hydroxyethyl) functionalized ILs, such as **1Tf**_**2**_**N** (143.1 mPa.s at 20°C), are significantly higher than their *N*-alkyl analogs (e.g., *N*-ethylpyridinium [Tf_2_N]; 47.4 mPa.s) (Bittner et al., 2012). On the other hand, the viscosity of the hydroxyl functionalized IL **1Tf**_**2**_**N** is much lower than the nitrile analog (326 mPa.s) (Lethesh et al., 2011).

### Electrochemical Stability

In general, ILs are known to show better stability toward oxidation and reduction over a wide range of voltage relative to conventional organic solvents (Armand et al., 2009). The difference in voltage (ΔE) between the onset potential of cathodic reduction (E_CL_) and anodic oxidation (E_AL_) is defined as the electrochemical potential window (EPW). While common organic solvents such as acetonitrile (ACN) and dimethylsulfoxide (DMSO) have a EPW of about 5.0 and 4.4 V, respectively (Younesi et al., 2014), EPW for a variety ILs exceeds 6.0 V (Hagiwara and Ito, 2000). ILs are considered to be suitable for a wide range of energy applications, such as supercapacitors, lithium-ion batteries, sodium-ion batteries, metal-air batteries, dye sensitized solar cells, etc (Fannin et al., 1984; Macfarlane et al., 2014; Watanabe et al., 2017). Table 2 reports the E_CL_, E_AL_, and EPW for all the 12 pyridinium-based ILs (**1-12Tf**_**2**_**N**) with various alkyl-substituents.

**Table 2:** Cathodic (E_CL_) and anodic potentials (E_AL_) of ILs (1-12Tf_2_N) vs. Ag/AgCl electrode (GC macro-electrode as a working electrode, 25°C).

**Entry**	**ILs**	**E_**CL**_ (V)**	**E_**AL**_ (V)**	**EPW(V)**
1	1Tf_2_N	−1.13	2.76	3.89
2	2Tf_2_N	−1.34	2.71	4.05
3	3Tf_2_N	−1.29	2.71	4.00
4	4Tf_2_N	−1.54	2.71	4.25
5	5Tf_2_N	−1.31	2.63	3.94
6	6Tf_2_N	−1.30	2.23	3.53
7	7Tf_2_N	−2.83	2.61	5.44
8	8Tf_2_N	−1.14	2.58	3.72
9	9Tf_2_N	−1.25	2.72	3.97
10	10Tf_2_N	−1.19	1.81	3.00
11	11Tf_2_N	−1.01	2.52	3.53
12	12Tf_2_N	−1.21	2.73	3.94

EPW of the ILs are determined by both the cations and anions, where the reduction is caused by cations and the oxidation by anions (Mcewen et al., 1999; Suarez et al., 2002; Rogers et al., 2009). In general, [Tf_2_N] anions are known to show excellent stability at high oxidative potentials. It has been reported (Yeon et al., 2005) that the EPW of morpholinium and pyrrolidinium ILs with [Tf_2_N] anion is significantly higher than for other anions, such as trifluoromethanesulfonate [TfO], dicyanamide [N(CN)_2_], and trifluoroacetate [TFA]. This trend clearly correlates with the high E_AL_ observed for all the [Tf_2_N]-based ILs (**1-12Tf**_**2**_**N**) within the range of 2.23–2.76 Vregardless of the cation structure. This trend provides a clear indication that the anionic decomposition is mainly influenced by the oxidation of [Tf_2_N] anion irrespective of the alkyl chain length. Similar conclusions are reported for a series of [Tf_2_N]-based pyrrolidinium ILs with varying alkyl chain length (Appetecchi et al., 2009). However, the effect of alkyl chain length is found to have a pronounced effect on the cathodic stability. Increased alkyl chain length mostly reduced the E_CL_ values, as seen by the values for 3-methyl IL (**3Tf**_**2**_**N**, −1.29 V) and 3-ethyl IL (**10Tf**_**2**_**N**, −1.19 V) and correspondingly, 4-methyl IL (**4Tf**_**2**_**N**, −1.70 V) and 4-ethyl IL (**11Tf**_**2**_**N**, −1.01 V). This can be explained by the adverse effect induced by longer alkyl chain length on the cation, increasing the possibility for the cations to undergo reductive degradation through dealkylation. During this step, unstable aliphatic free radical is formed from the alkyl group, to introduce cleavage and decomposition of the cationic IL core (Morrison and Boyd, 1992).

**Figure 5:**
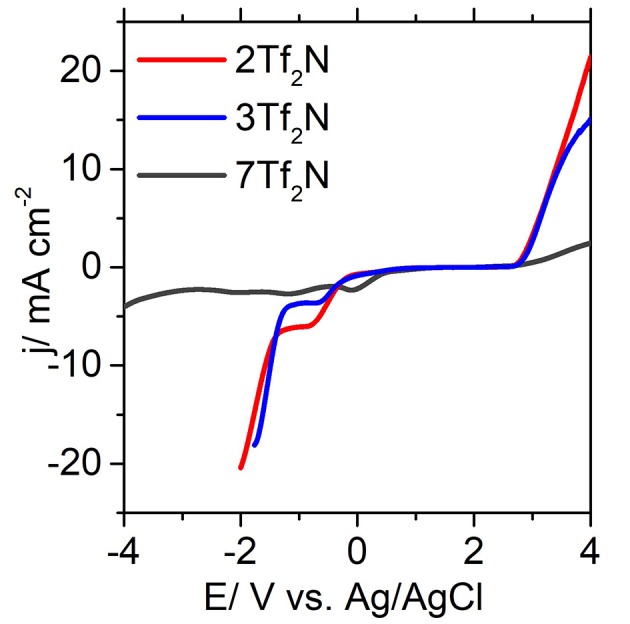
Forward scan of selected cyclic voltammograms of **2Tf**_**2**_**N, 3Tf**_**2**_**N, and 7Tf**_**2**_**N** obtained with glassy carbon electrode.

The position of the alkyl groups also plays a crucial role in determining the E_CL_. as observed from the noticable reduction of E_CL_ going from 2-methyl **2Tf**_**2**_**N** (−1.34 V) to 3-methyl **3Tf**_**2**_**N** (−1.29 V) and correspondingly from the 2-ethyl **9Tf**_**2**_**N** (−1.25 V) to the 3-ethyl **10Tf**_**2**_**N** (−1.19 V) in Figure 5. These observations may be explained by the steric hindrance and shielding effects, which are reduced by an increased distance from the pyridinium nitrogen (Makino et al., 2013) to the 2- and 3-alkyl group. These effects consequently affects the cathodic stability of the 3-alkyl ILs. The strongest effect of steric hindrance is seen for 2,6-dimethyl IL **7Tf**_**2**_**N** with the highest cathodic stability of −2.83 V thus widening the EPW to 5.44 V. Overall, the EPW for the ILs (**1-12Tf**_**2**_**N**) are in the range 3.00–5.44 V, which clearly showed their excellent potential for electrochemical applications.

### Electrical Conductivity

Estimated values for electrical conductivity of ILs **1-12Tf**_**2**_**N** at 25°C was obtained by DFT/COSMO-RS calculation (Table 3; Liu et al., 2010). The electrical conductivity (0.233 κ/S m^−1^) of the non-alkylated **1Tf**_**2**_**N** was in accordance with previous experimental data (Makino et al., 2013), indicating good agreement of predicted values with experimental results. An inverse relationship of electrical conductivity with viscosity was observed. It is known that the mobility of ions increases to give higher electrical conductivity as the viscosity decreases (Fannin et al., 1984). This observation is seen for the 3-methyl/ethyl ILs **3Tf**_**2**_**N** and **10Tf**_**2**_**N** with the highest conductivity values (0.366–0.383 κ/S m^−1^) among the synthesized ILs. Correspondingly, the viscosity of these ILs at room temperature are the lowest of the tested ILs; **3Tf**_**2**_**N** (112.2 mPa.s) and **10Tf**_**2**_**N** (116.6 mPa.s) (Figure 4). This trend is in accordance with reported data for electrical conductivity (Liu et al., 2015) of hydroxyethyl-imidazolium IL.

**Table 3:** Electrical conductivity, κ *(S m*^−1^*)*, of ILs (1-12Tf_2_N) predicted ^[13]^ at 25°C.

**Entry**	**ILS**	**κ (S m^−1^)**	**ILS**	**κ (S m^−1^)**
1	1Tf_2_N	0.233	7Tf_2_N	0.19
2	2Tf_2_N	0.311	8Tf_2_N	0.238
3	3Tf_2_N	0.383	9Tf_2_N	0.284
4	4Tf_2_N	0.276	10Tf_2_N	0.366
5	5Tf_2_N	0.260	11Tf_2_N	0.236
6	6Tf_2_N	0.234	12Tf_2_N	0.146

### Charge Distribution

**Figure 6:**
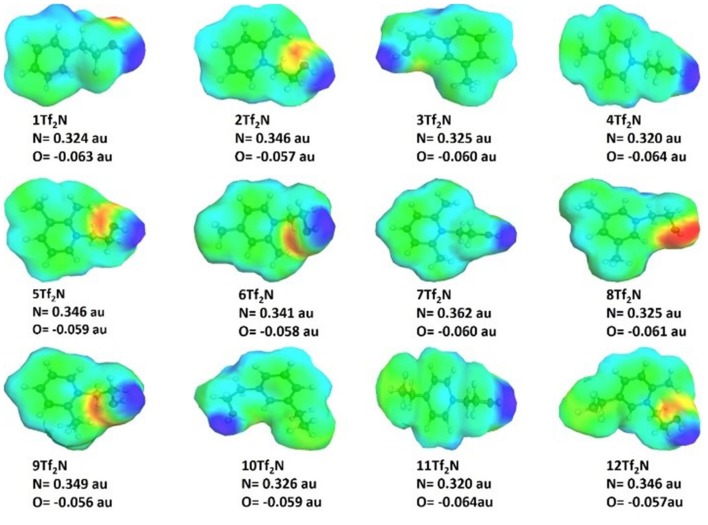
Nitrogen and oxygen charge distribution in the hydroxyl functionalized pyridinium ILs **1-12Tf**_**2**_**N** (only cations showed). The blue color represents positive COSMO charge density and the negative charge density regions are shown in red.

The charge distribution in the ILs (**1-12Tf**_**2**_**N**) pyridinium cations (Figure 6) was calculated using COSMO-RS to study how pyridinium-alkylation affected the charges on the nitrogen and oxygen atoms. The calculations showed that the partial positive charge on the pyridinium nitrogen atom mostly varied by the position of pyridinium alkylation, while analogous methyl and ethyl derivatives had very similar partial negative charge (e.g., 4-methyl IL **4Tf**_**2**_**N** and 4-ethyl IL **11Tf**_**2**_**N**; −0.320 au). 2-Alkylation had considerably stronger effect than alkylation in other positions, as seen by the higher partial positive nitrogen charge (higher than −0.340 au) for all the 2-methyl/ethyl substituted IL compared to other substituted ILs. In particular, the 2,6-dimethyl IL **7Tf**_**2**_**N** (0.3627 au) had the maximum partial positive charge on nitrogen. There is no significant difference in the positive nitrogen charge of 3- and 4-methyl/ethyl IL derivatives (e.g., **3Tf**_**2**_**N** and **4Tf**_**2**_**N**).

Alkylation had minor influence on the partial negative charge on hydroxyl oxygen, which varied from −0.056 auto −0.064 au for 2-ethyl IL **9Tf**_**2**_**N** and 4-ethyl IL **11Tf**_**2**_**N**, respectively. Analogous methyl and ethyl derivatives had very similar partial oxygen negative charge, e.g., 4-methyl IL **4Tf**_**2**_**N** and 4-ethyl IL **11Tf**_**2**_**N** (−0.064 au). The results are in agreement with the previous findings for nitrile-functionalized ILs, showing that the partial negative charge on the nitrile group varies only according to the spacer length between the pyridine nitrogen atom and the nitrile functionality (Lethesh et al., 2011). Pyridinium alkylation had no influence on partial negative charge of the nitrile functionality.

## Conclusions

A series of 12 new hydroxyl functionalized pyridinium ILs **1-12Tf**_**2**_**N**, with Tf_2_N anions were synthesized in order to study the effect of pyridine alkyl-substitution on ILs physicochemical and electrochemical properties. The ILs **1-12Tf**_**2**_**N** are thermally stable up to 405°C. The measured thermal stability values of the ILs are in reasonable agreement with corresponding predicted values. The deviations were mainly within the calculated prediction uncertainties. Substitution on the pyridine ring affected the density, which, in general, dropped by increased alkyl substitution. The deviation between the predicted and experimental values of density is small at all the measured temperatures. Methyl or ethyl groups on the pyridine ring had strong influence on the IL viscosity, increasing by the number, and position of alkyl groups. The deviation between the measured and calculated viscosity value was low at elevated temperatures, but high at low temperature. Cyclic voltammetry studies showed that the synthesized ILs (**1-12Tf**_**2**_**N**) have a promising electrochemical window between 3.0 and 5.54 V. The calculated charge distribution on the cationic core revealed that the partial positive charge on the pyridinium nitrogen atom varies according to the position of the methyl/ethyl substituents. The partial negative charge on the hydroxyl oxygen is not affected by the alkyl substituents. The present experimental and theoretical study contributes to an increased knowledge of important properties of functionalized ILs, and clearly shows their excellent potential for electrochemical applications. Mg complexes based on the new hydroxyl functionalized pyridinium ILs have been prepared, and on-going studies show promising results for the Mg-IL compounds for high current density electrodeposition of magnesium.

## Data Availability

All datasets generated for this study are included in the manuscript/Supplementary Files.

## Author Contributions

KL conceived the idea, performed the synthesis and characterization of ILs, and wrote the manuscript. SE performed the thermal stability, melting point, and glass transition analysis. JR and KJ performed the electrochemical study. DR performed the density and viscosity measurement. VV conducted the DFT/COSMO-RS study. AF supervised KL and SE and helped to write the manuscript.

### Conflict of Interest Statement

The authors declare that the research was conducted in the absence of any commercial or financial relationships that could be construed as a potential conflict of interest.
