# Supplementary material for: Hydroxyl Functionalized Pyridinium Ionic Liquids: Experimental and Theoretical Study on Physicochemical and Electrochemical Properties
Source: Front Chem. 2019 Sep 18;7:625. doi: 10.3389/fchem.2019.00625 (PMC6759651; doi:10.3389/fchem.2019.00625)
Supplement: Supplementary file 1 [file Table_1.DOCX]

**Supporting Information**

**Hydroxyl functionalized pyridinium ionic liquids;**

**Experimental and theoretical study on physicochemical and electrical properties**

Kallidanthiyil Chellappan Lethesh, *^1^ Sigvart Evjen,^1^ Jaganathan Joshua Raj,^2^ Denis C D Roux,^3^

Vishwesh Venkatraman,^1^ Kaushik Jayasayee,^4^ and Anne Fiksdahl ^1^

1. *Department of Chemistry, Norwegian University of Science and Technology (NTNU), Trondheim*
2. *Université Grenoble Alpes (UGA), BP53 Domaine Universitaire, 38610 Gières,*

*Grenoble Cedex 09, France*

1. *Universiti Teknologi PETRONAS, Bandar Seri Iskandar, 32610-Perak, Malaysia*
2. *New energy Solutions, SINTEF Industry, Trondheim*

**Physical properties** S-2

**^1^H and ^13^C NMR**

1. bromide salts; **1-12Br** S-3
2. ILs; **1-12Tf_2_N** S-15

**Cyclic voltammetry of 1-12Tf_2_N** S-27

**Thermal decomposition temperature (T_d_) of 1-12Tf_2_N and 1-12Br;**

experimental and predicted S-29

**Density values of 1-12Tf_2_N**; experimental and predicted S-30

**Viscosity of 1-12Tf_2_N**; experimental and predicted S-31

**Physical properties**

*Viscosity* measurements were carried out with a rolling ball viscosimeter Lovis 2000 M/ME from Anton Paar GbH company, Austria. Kinematic viscosity determination is based on the time of a rolling ball, submitted to the gravity, to travel along a determined distance. Depending on the viscosity, a glass capillary tube (diameter 1.59mm or 2.5mm) was used; inside a stainless-steel ball (diameter= 1.5mm, density = 7.68g/cm^-3^) was inserted. The dynamic viscosity is the ratio of the kinematic viscosity divided by the density. *Density* was measured with a densitometer DM4500 from Anton Paar company, Austria. The density determination is based on the shift of the natural frequency of an oscillating U tube, filled with a fluid. The viscosity and the density meter were equipped with a temperature controller with an accuracy of 0.02 °C in the range of 5 °C to 80 °C. Viscosity and density were measured by applying an identical temperature sweep. To check a non-Newtonian behavior for each temperature, different inclinations of the capillary tube were imposed, allowing to maximize the shear rate. *Melting points (T_m_*) and *glass transition (T_g_) temperatures* of ILs were measured with a Differential Scanning Calorimetry (DSC, Perkin Elmer, model Pyris1, USA) with a heating rate of 10 °C/min under nitrogen flow. *Thermal decomposition* temperatures of the ILs were measured using a Thermal Gravimetric analyzer (TGA, Perkin Elmer, Pyris V-3.81, USA) with a heating rate of 10 °C/min under nitrogen atmosphere.

**a) ^1^H and ^13^C NMR of bromide salts; 1-12Br**


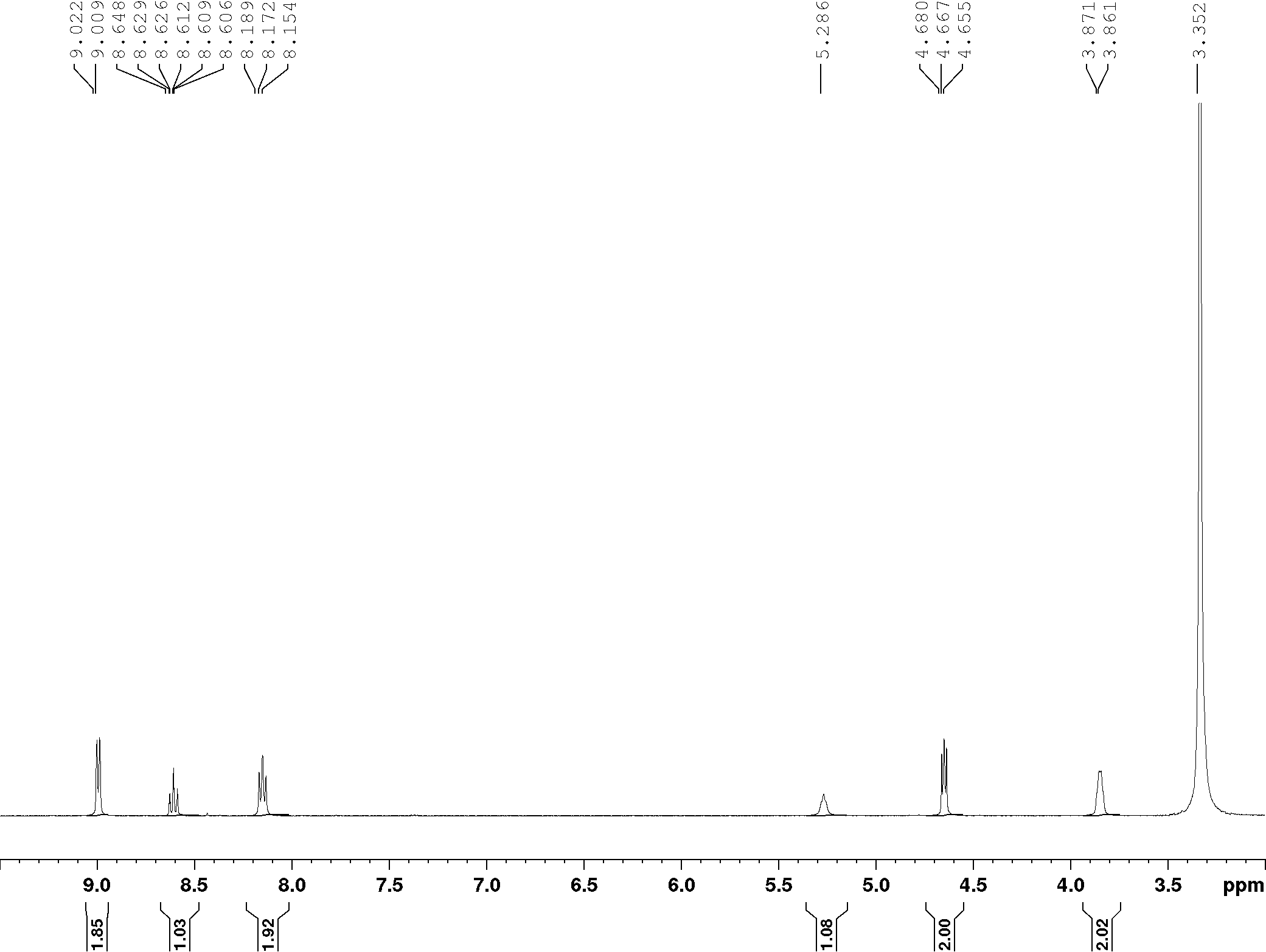


**Figure 1.** ^1^H NMR of 1-(2-hydroxyethyl)pyridinium bromide
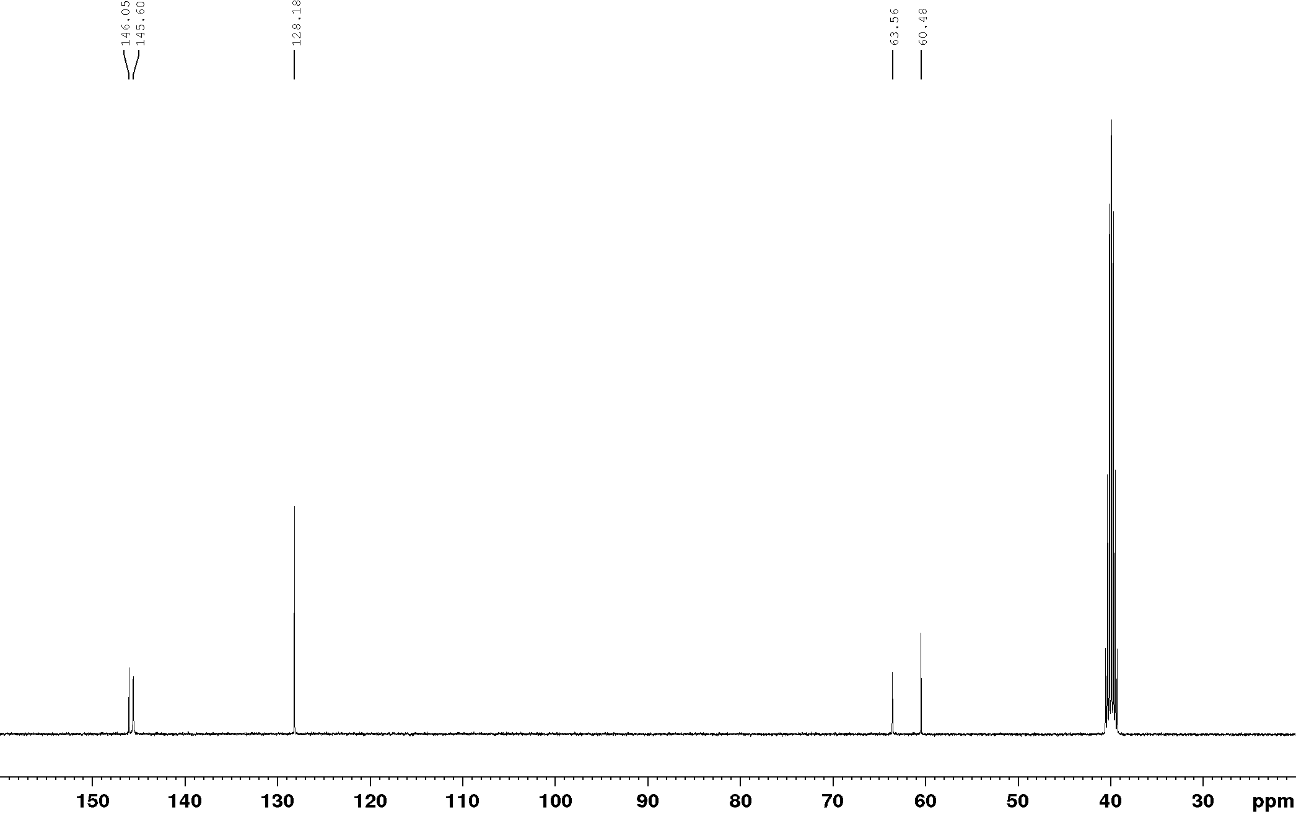
, **1Br**

**Figure 2.** ^13^C NMR of 1-(2-hydroxyethyl)pyridinium bromide, **1Br**


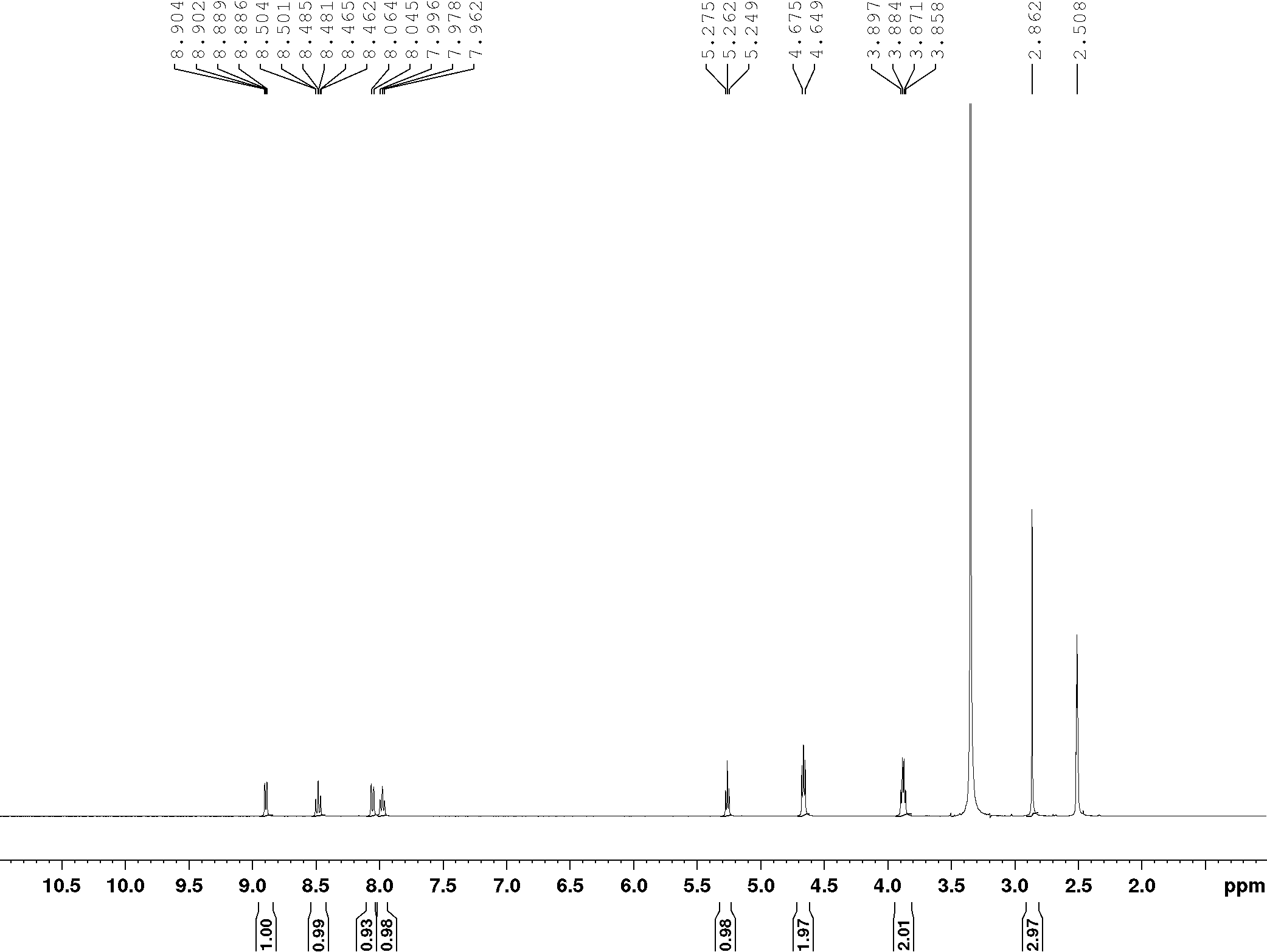


**Figure 3.** ^1^H NMR of 1-(2-hydroxyethyl)-2-methylpyridinium bromide, **2Br**


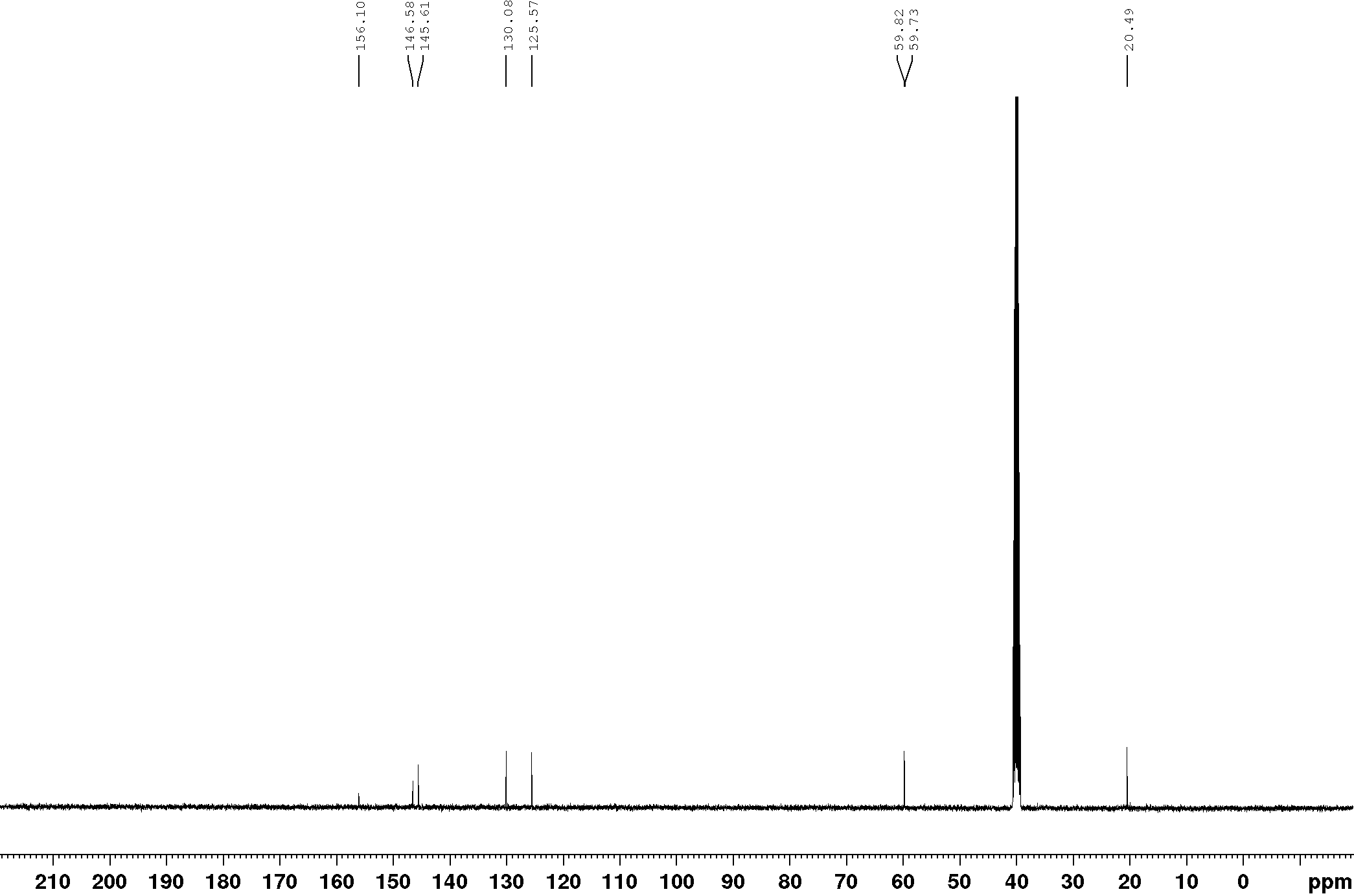


**Figure 4.** ^13^C NMR of 1-(2-hydroxyethyl)-2-methylpyridinium bromide, **2Br**


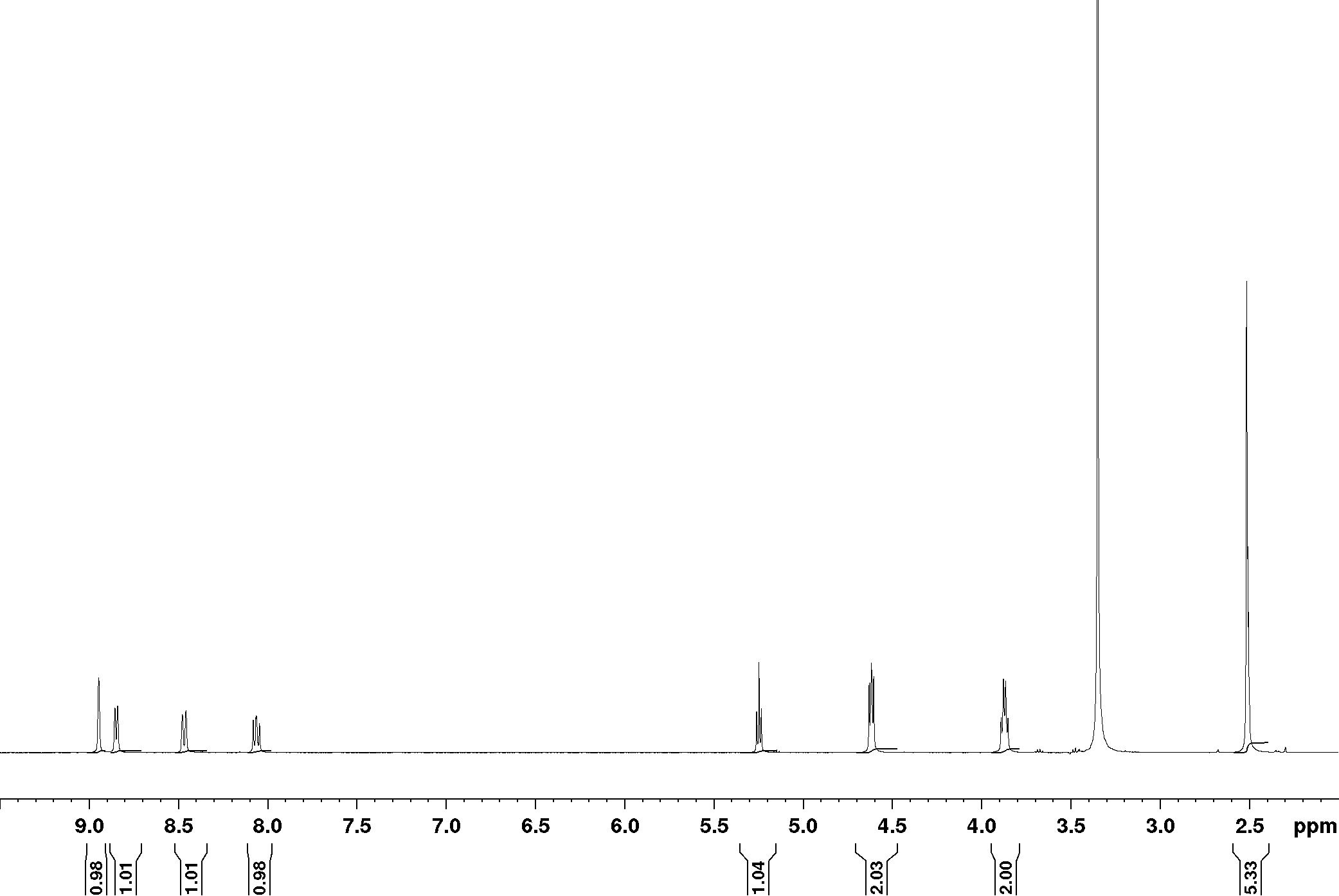


**Figure 5.** ^1^H NMR of 1-(2-hydroxyethyl)-3-methylpyridinium bromide, **3Br**


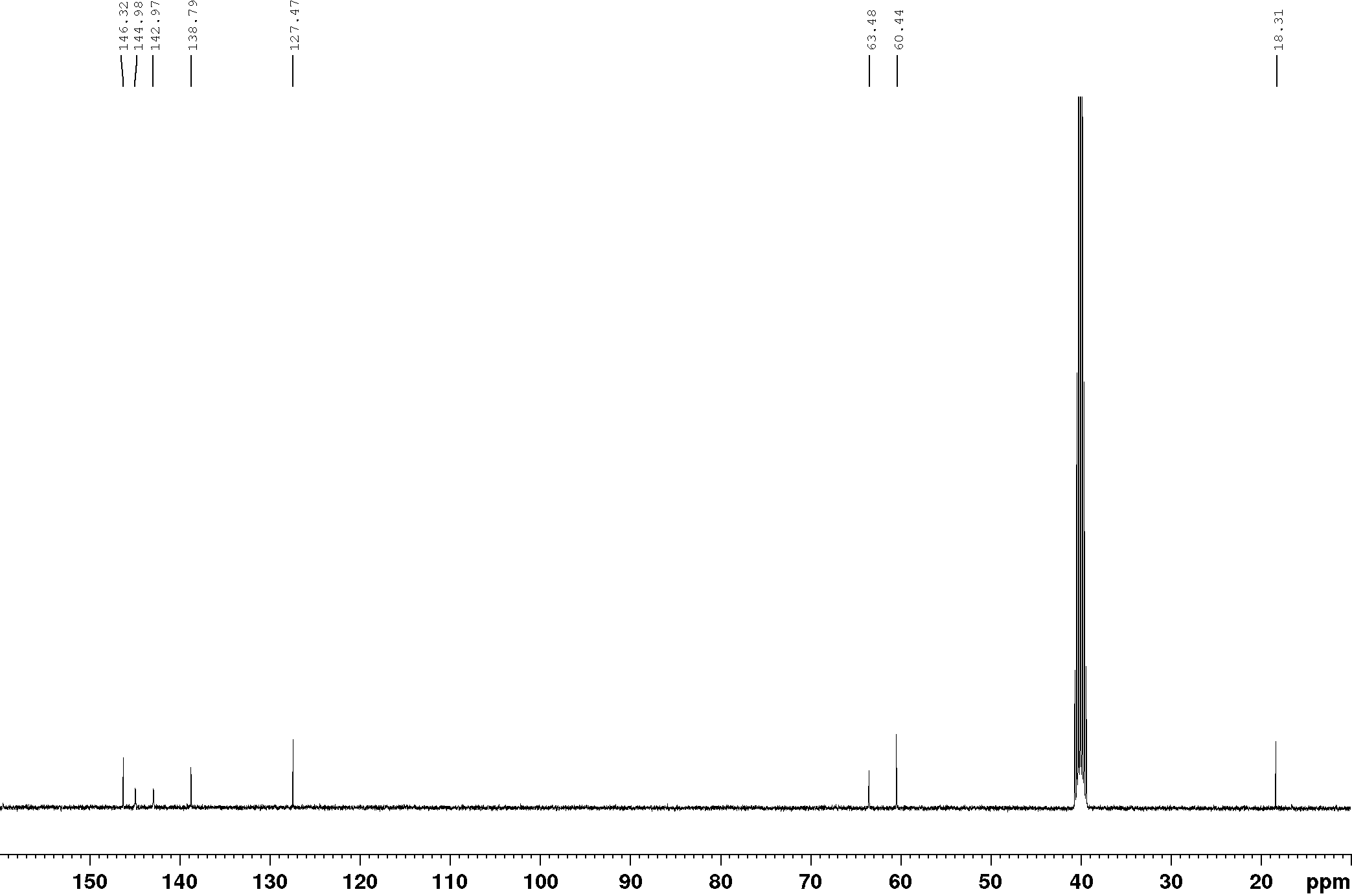


**Figure 6.** ^13^C NMR of 1-(2-hydroxyethyl)-3-methylpyridinium bromide, **3Br**


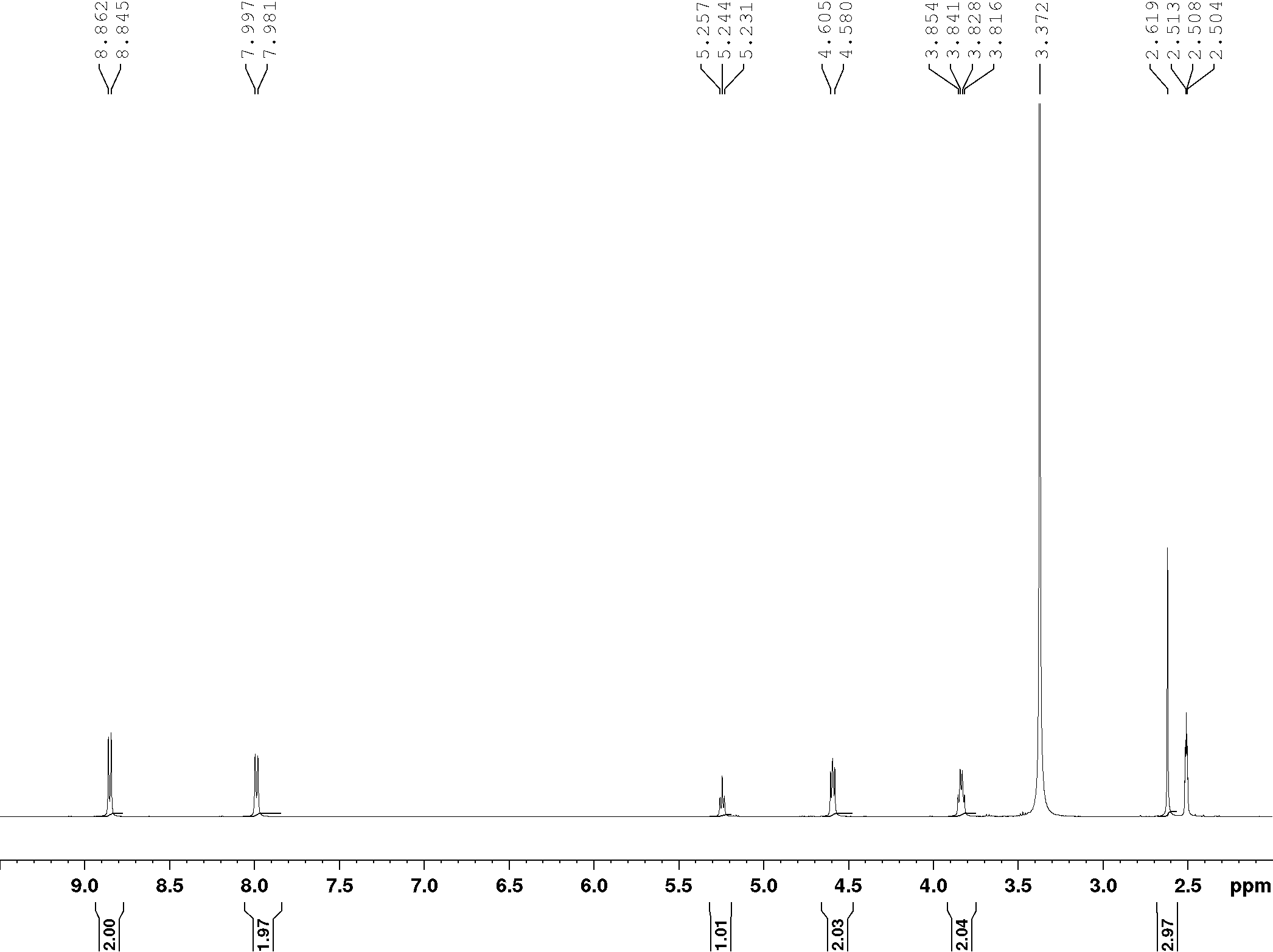


**Figure 7.** ^1^H NMR of 1-(2-hydroxyethyl)-4-methylpyridinium bromide, **4Br**


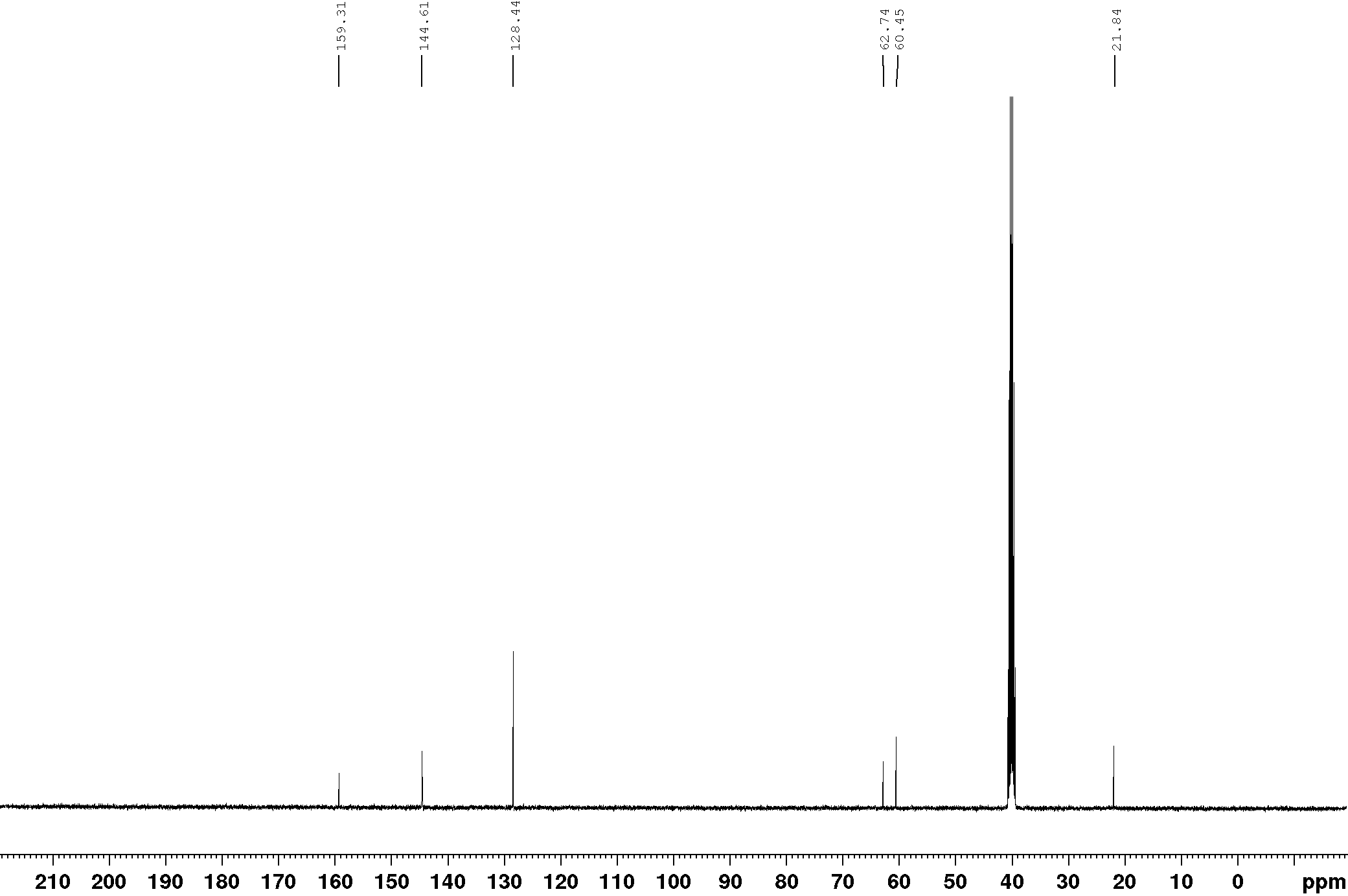


**Figure 8.** ^1^H NMR of 1-(2-hydroxyethyl)-4-methylpyridinium bromide, **4Br**


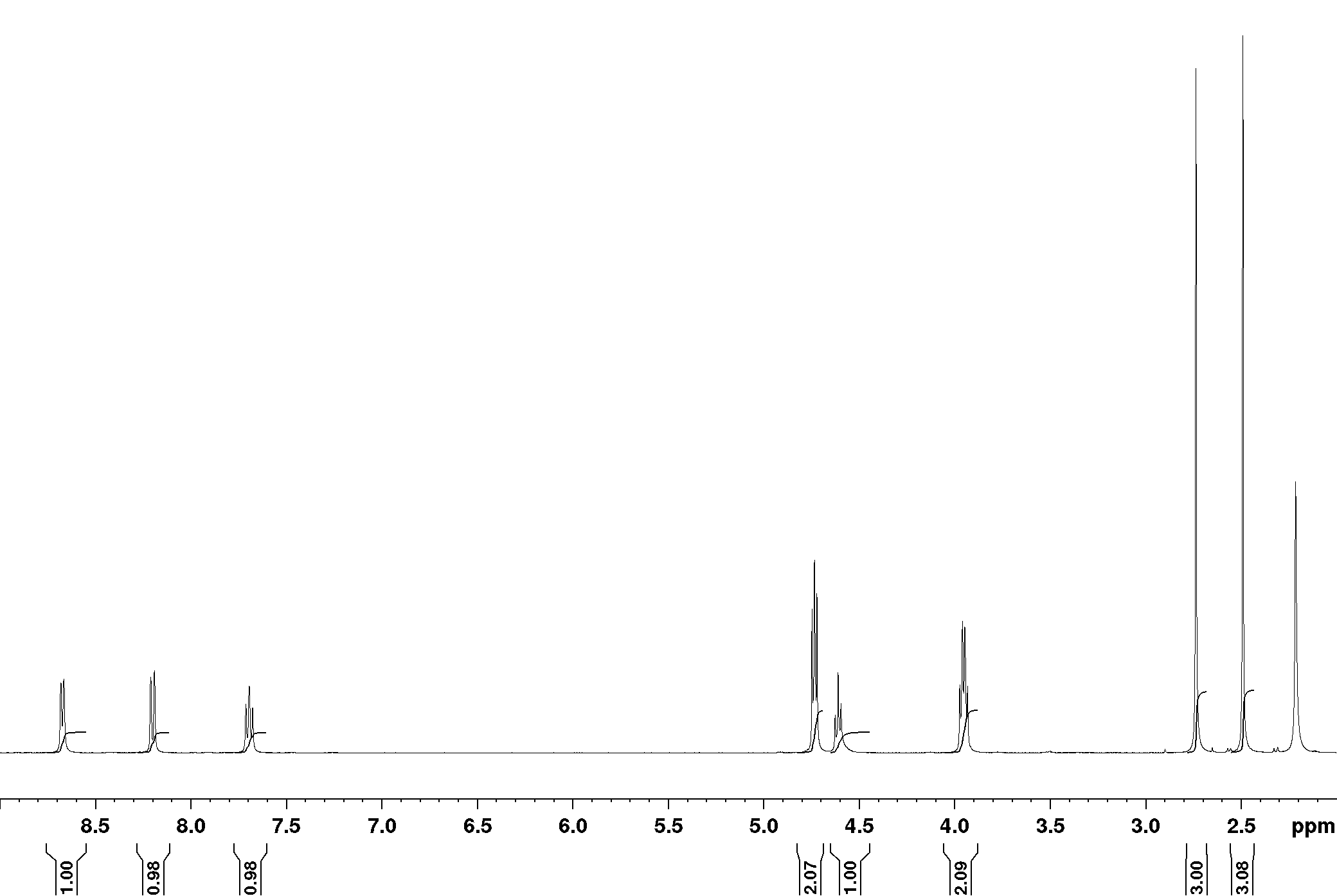


**Figure 9.** ^1^H NMR of 1-(2-hydroxyethyl)-2, 3-dimethylpyridinium bromide, **5Br**


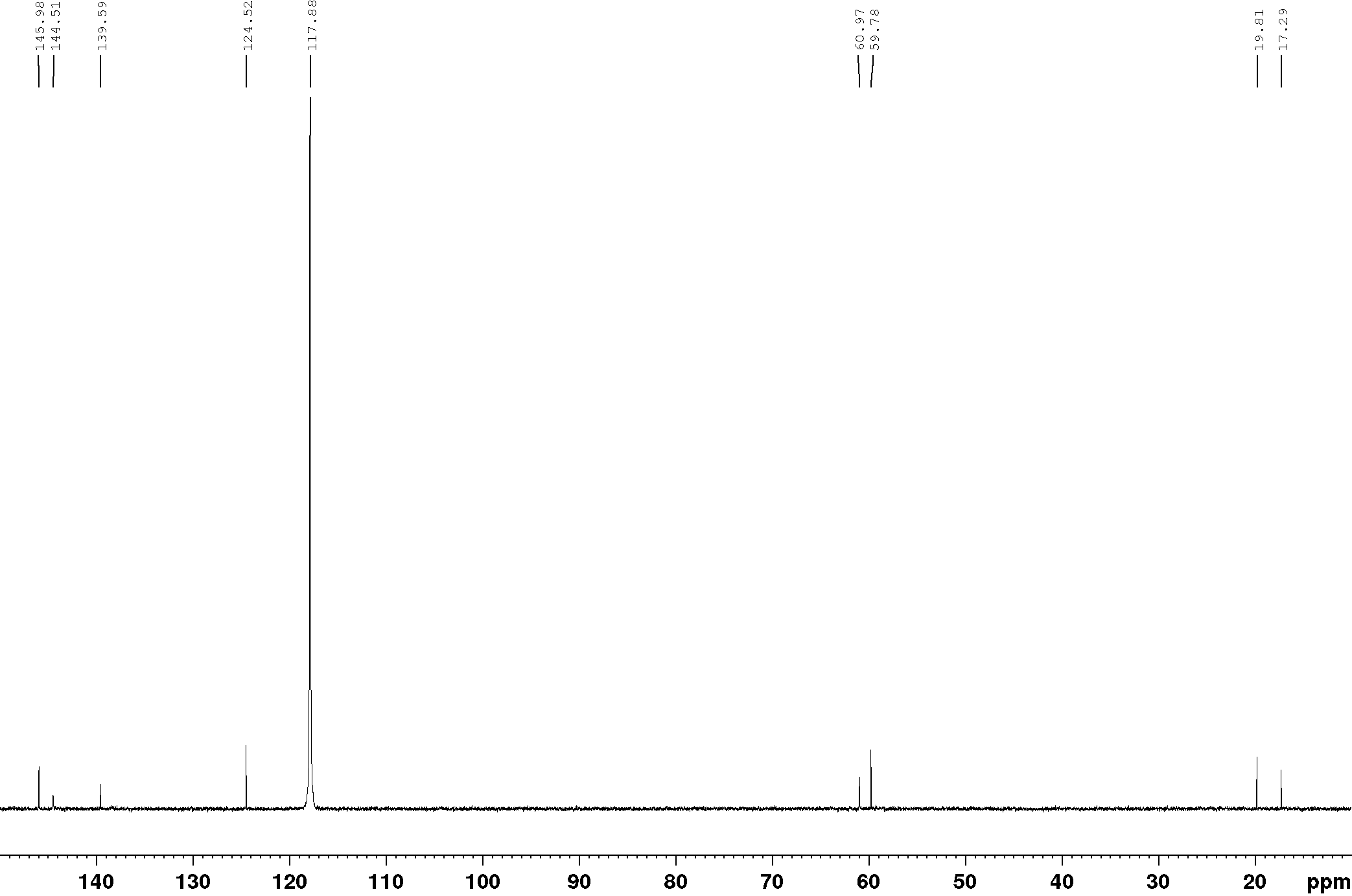


**Figure 10.** ^13^C NMR of 1-(2-hydroxyethyl)-2, 3-dimethylpyridinium bromide, **5Br**


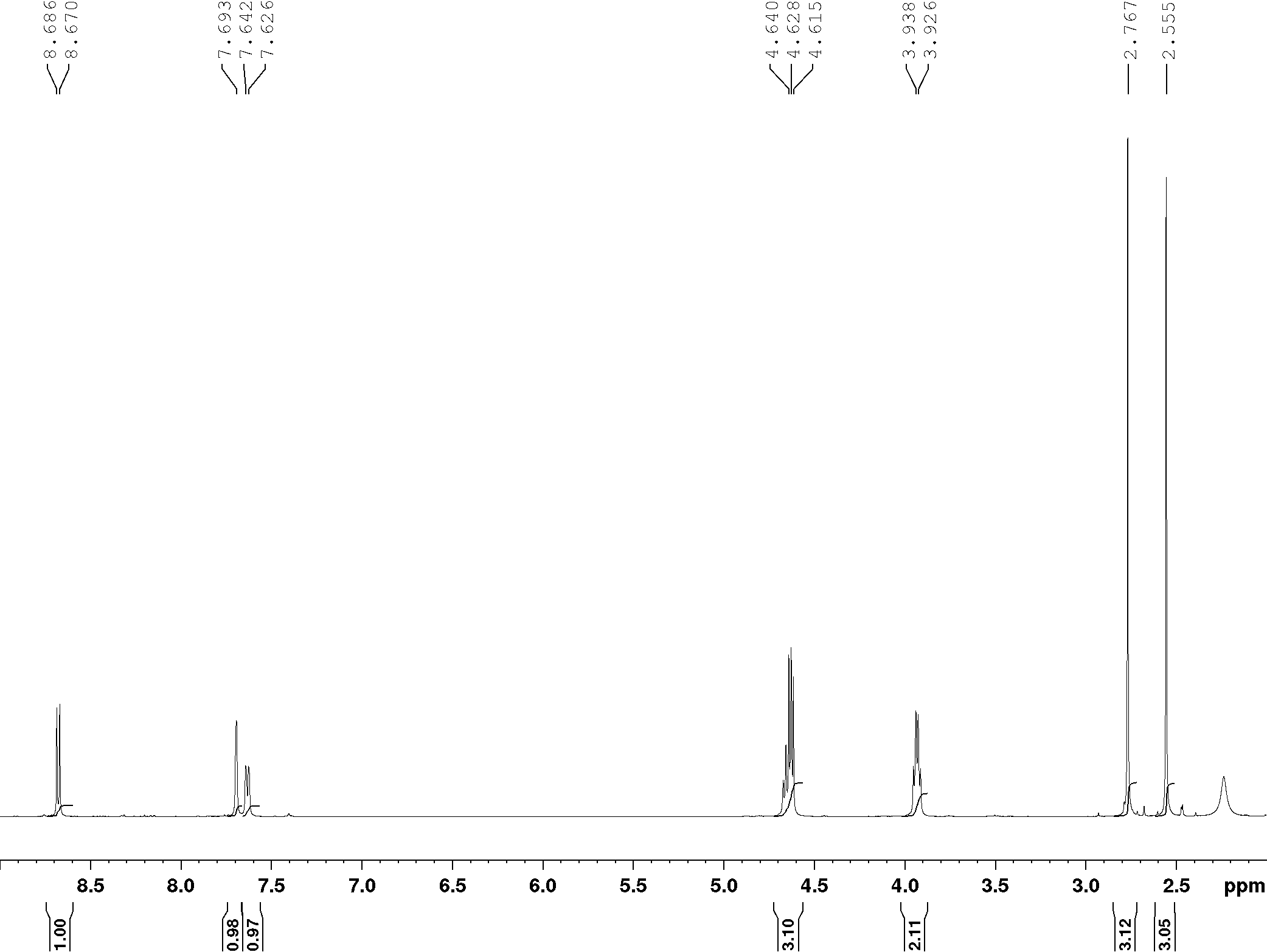


**Figure 11.** ^1^H NMR of 1-(2-hydroxyethyl)-2, 4-dimethylpyridinium bromide, **6Br**


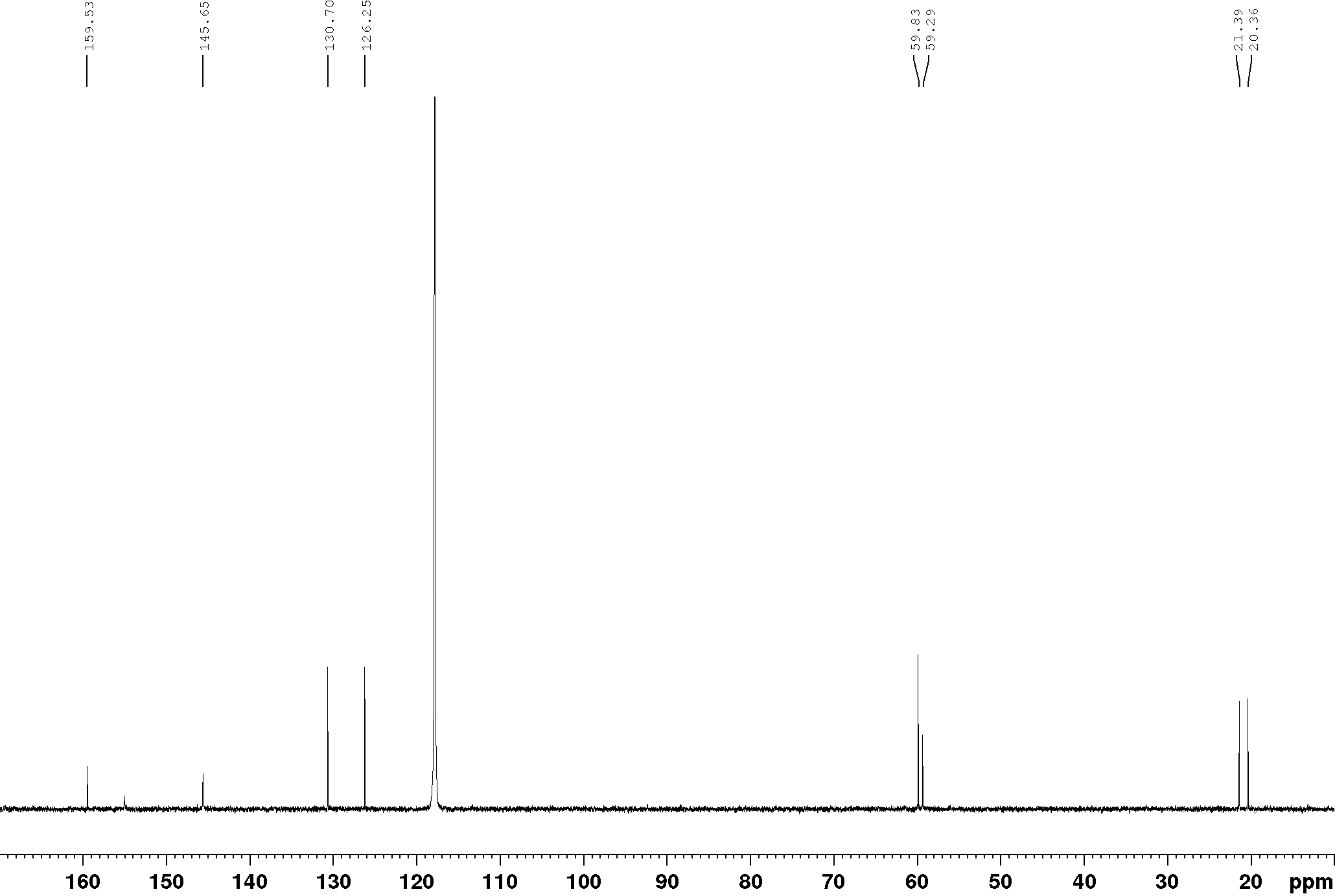


**Figure 12.** ^13^C NMR of 1-(2-hydroxyethyl)-2, 4-dimethylpyridinium bromide, **6Br**


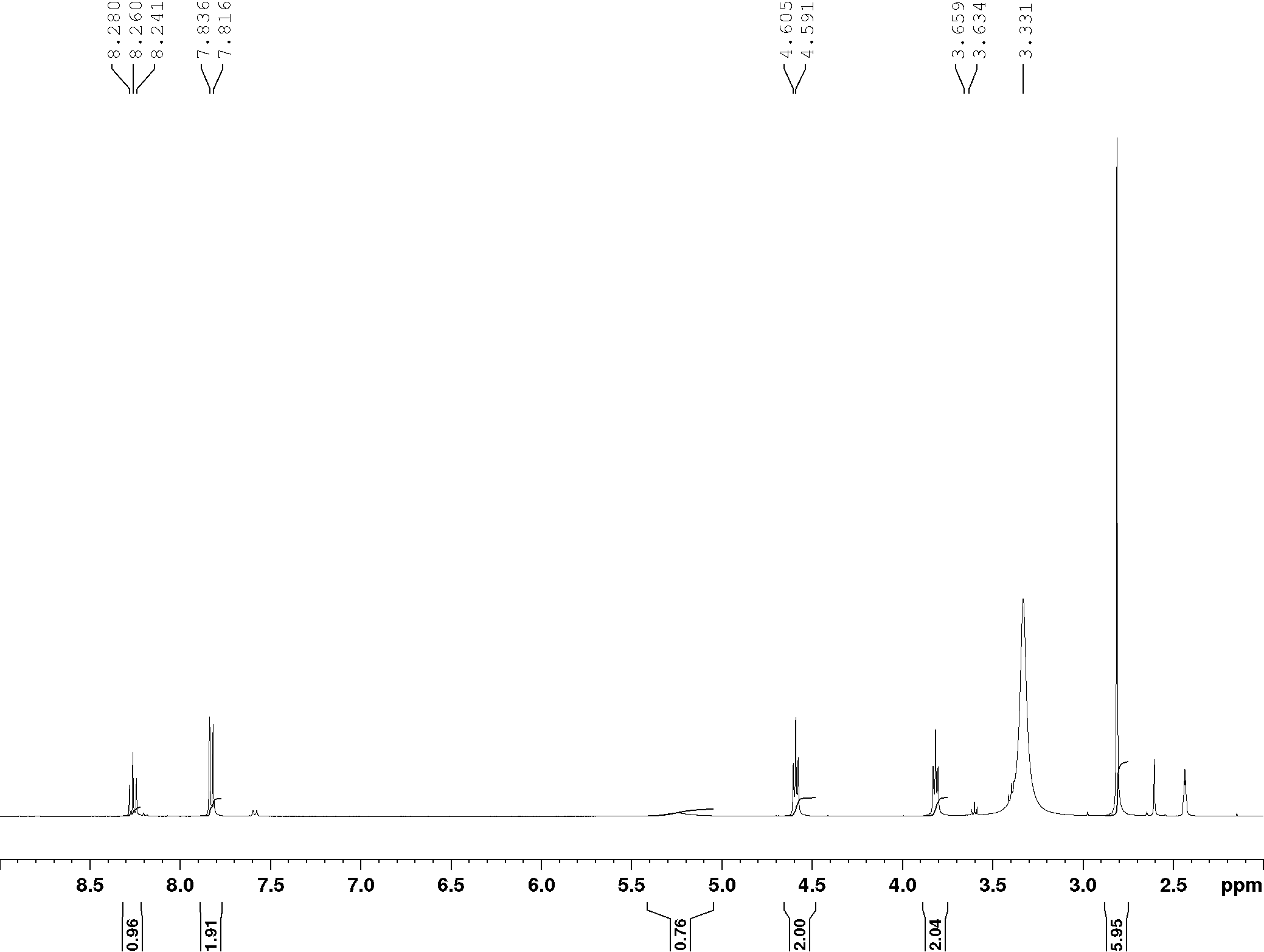


**Figure 13.** ^1^H NMR of 1-(2-hydroxyethyl)-2, 6-dimethylpyridinium bromide, **7Br**


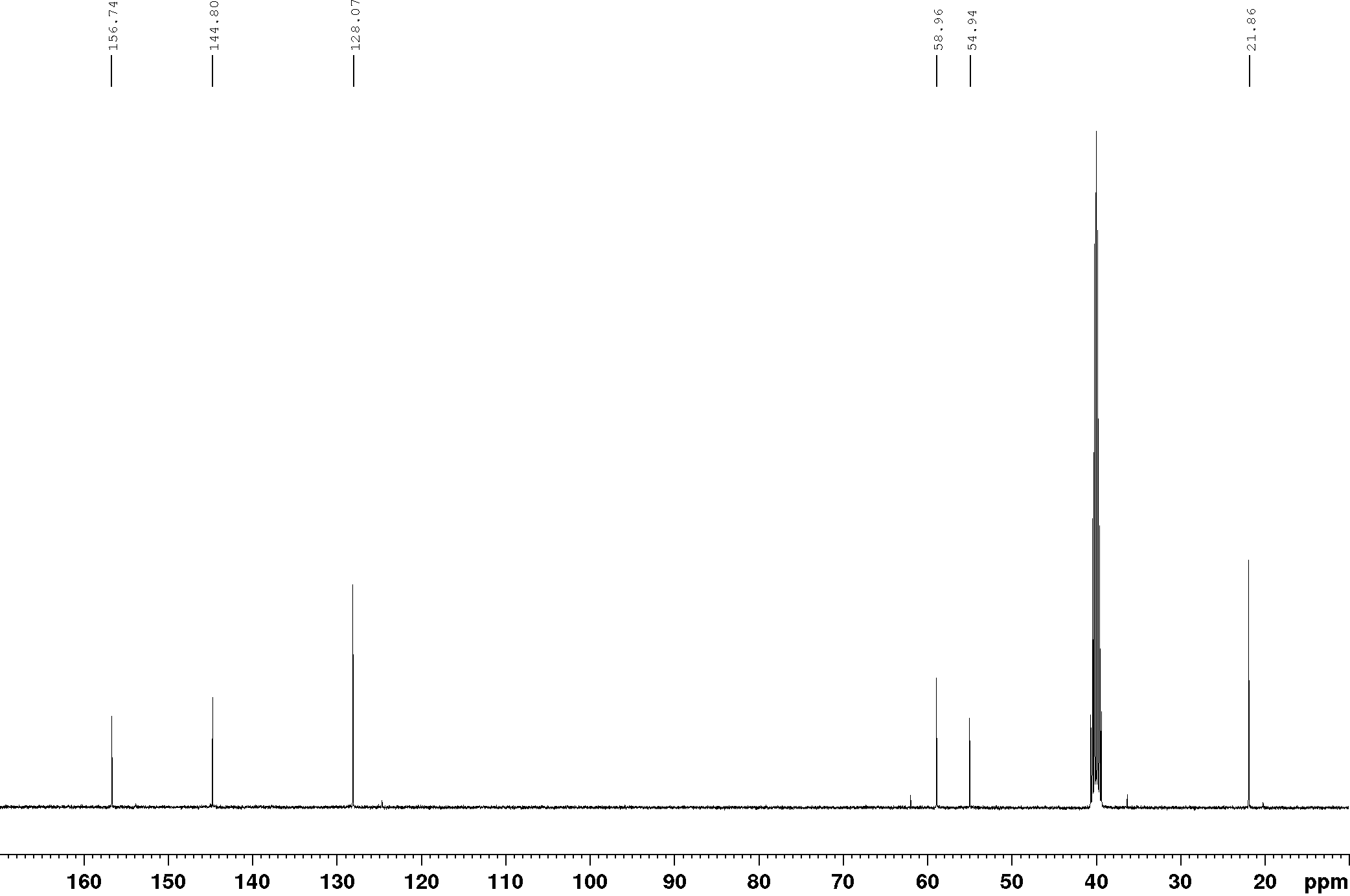


**Figure 14.** ^13^C NMR of 1-(2-hydroxyethyl)-2, 6-dimethylpyridinium bromide, **7Br**

**
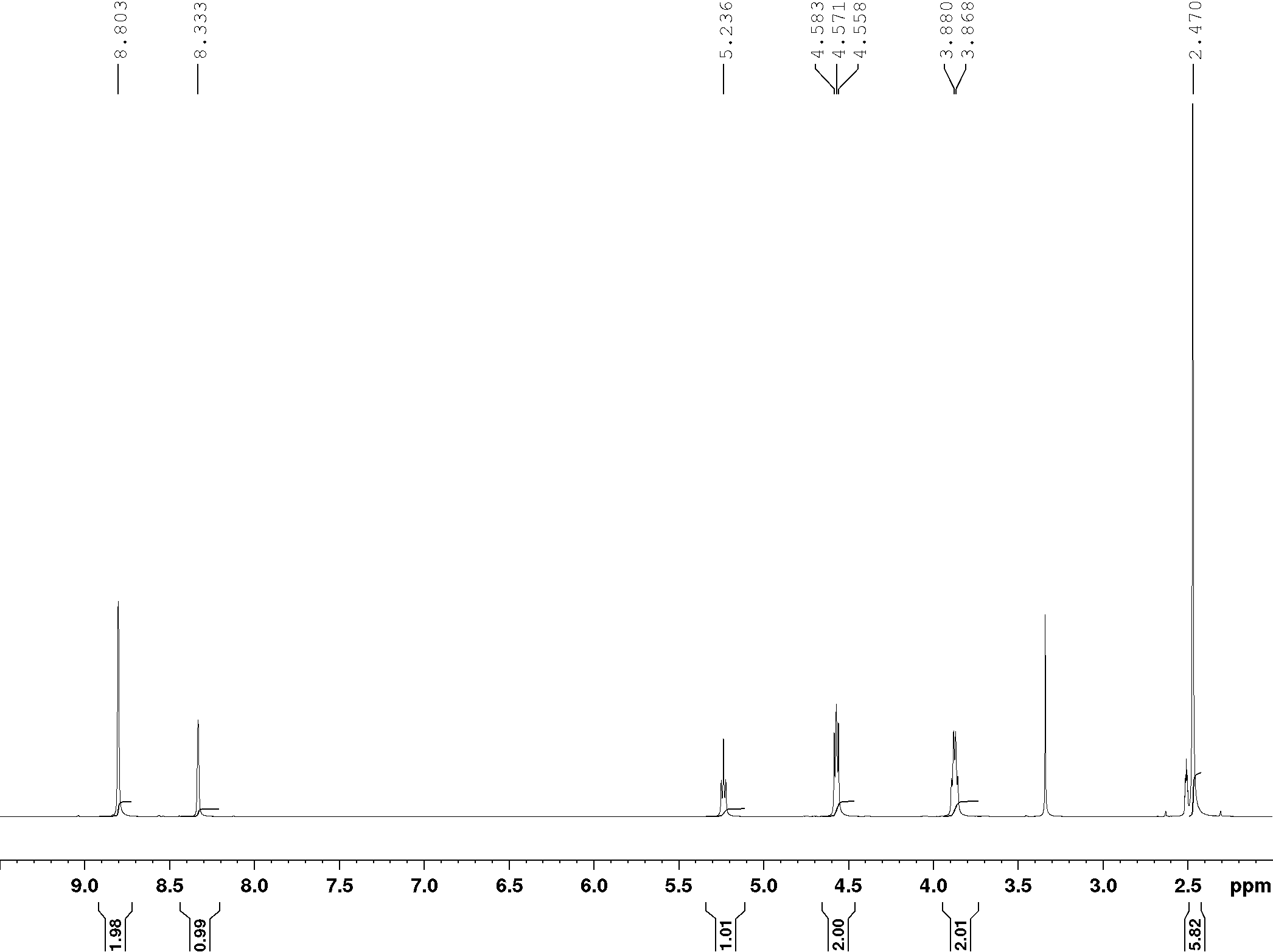
**

**Figure 15.** ^1^ H NMR of 1-(2-hydroxyethyl)-3, 5-dimethylpyridinium bromide, **8Br**

**
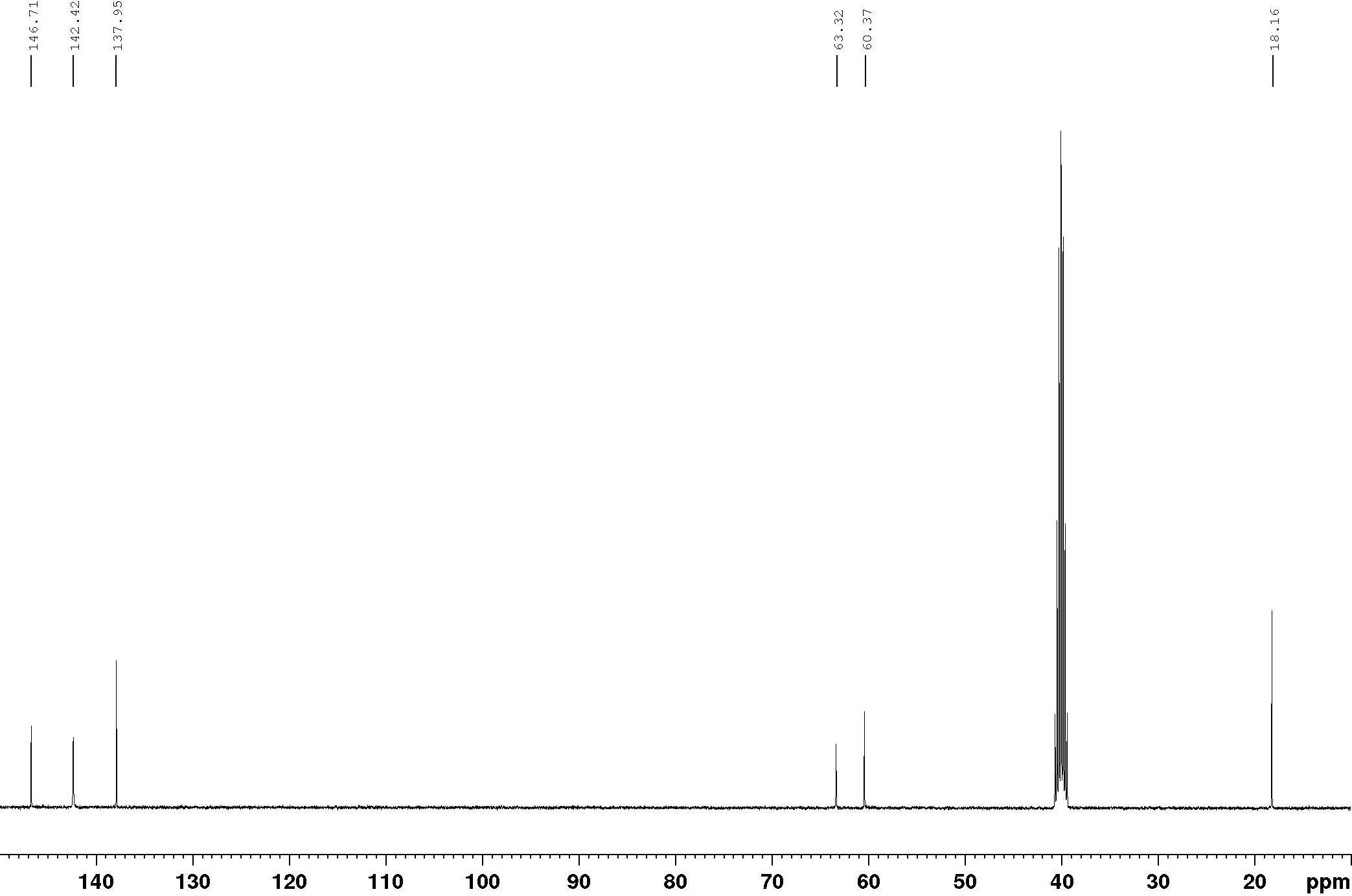
**

**Figure 16.** ^13^ C NMR of 1-(2-hydroxyethyl)-3, 5-dimethylpyridinium bromide, **8Br**


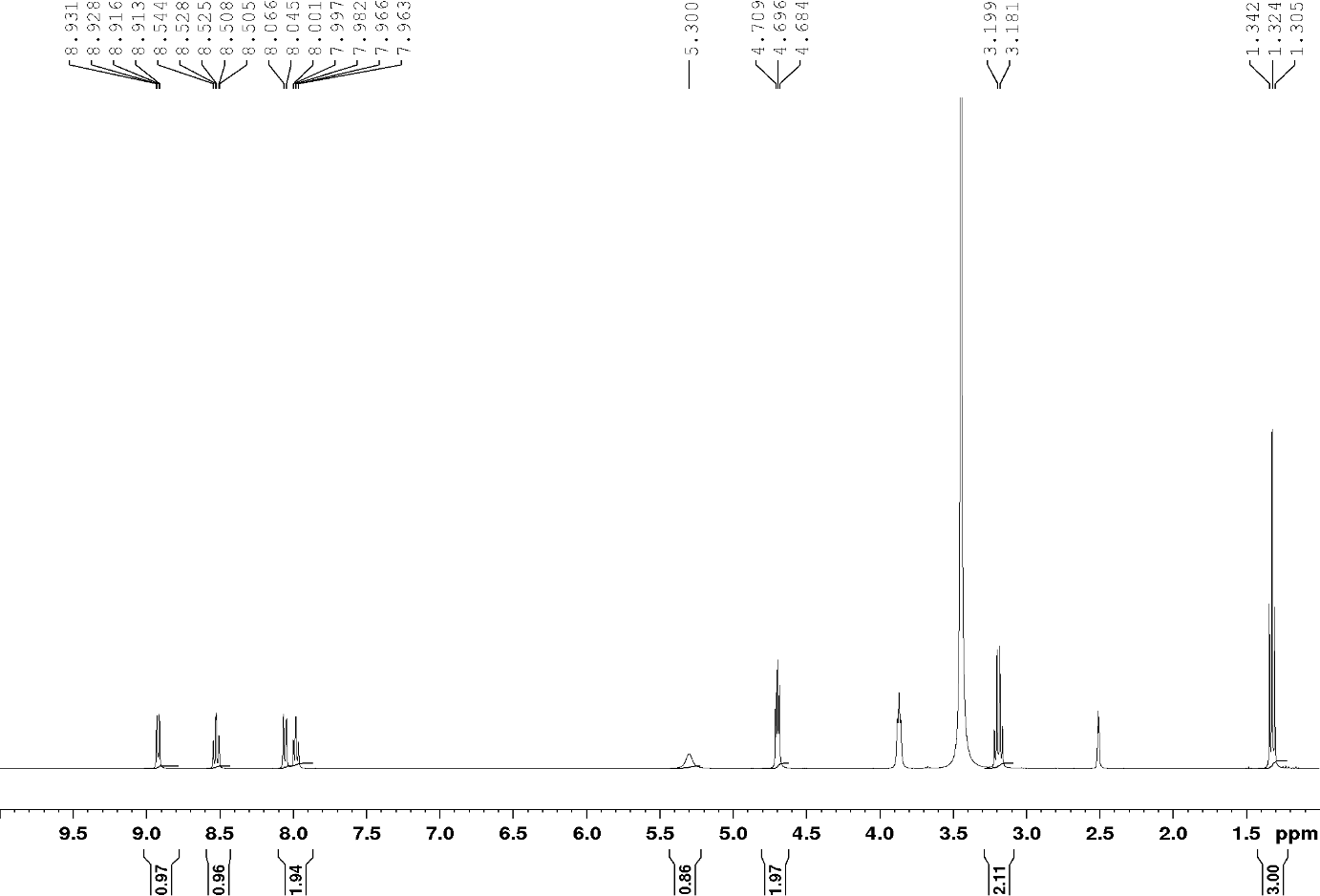
**Figure 17.** ^1^H NMR of 2-ethyl-1-(2-hydroxyethyl)pyridinium bromide, **9Br**

**
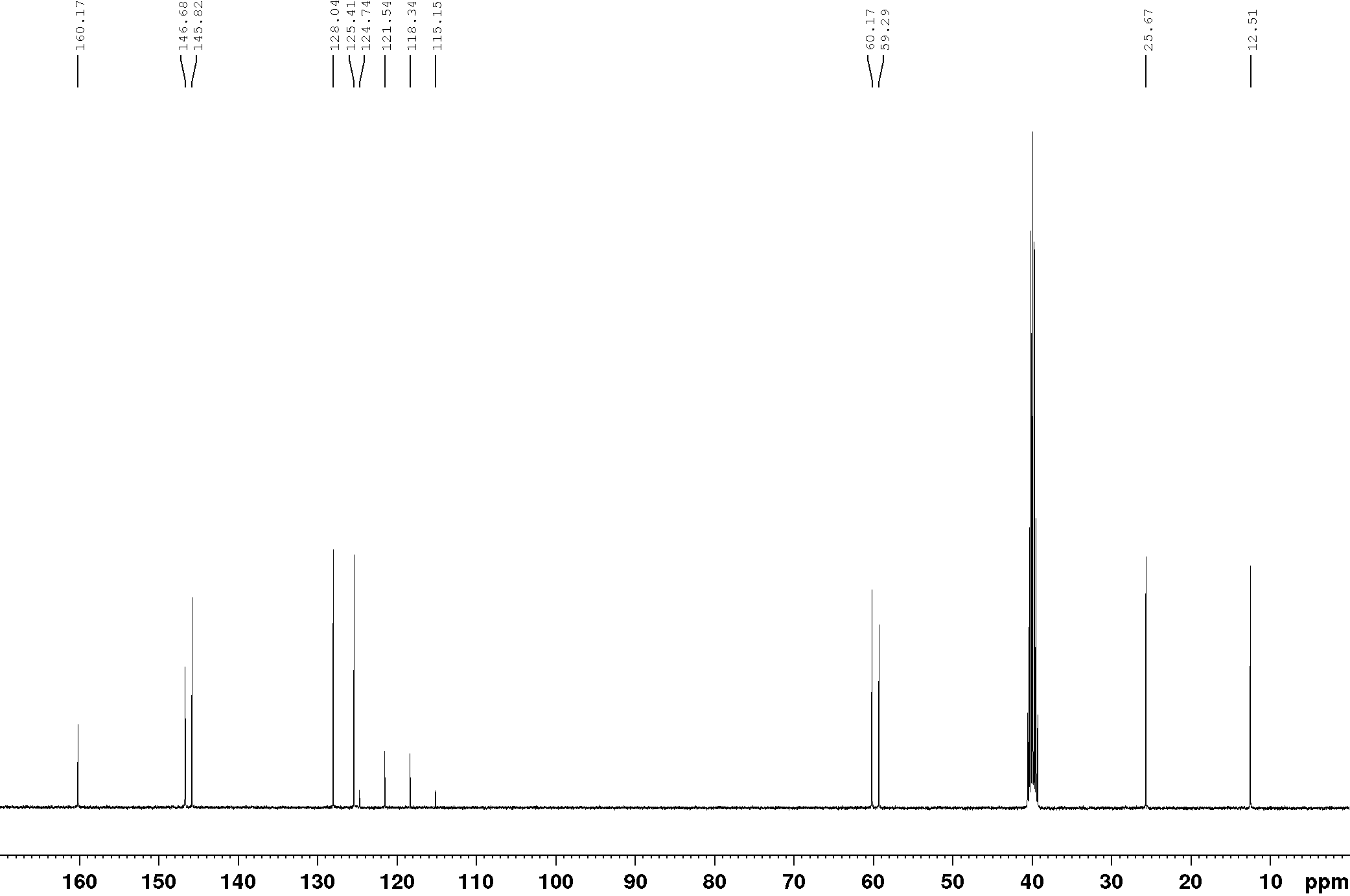
**

**Figure 18.** ^13^C NMR of 2-ethyl-1-(2-hydroxyethyl)pyridinium bromide, **9Br**


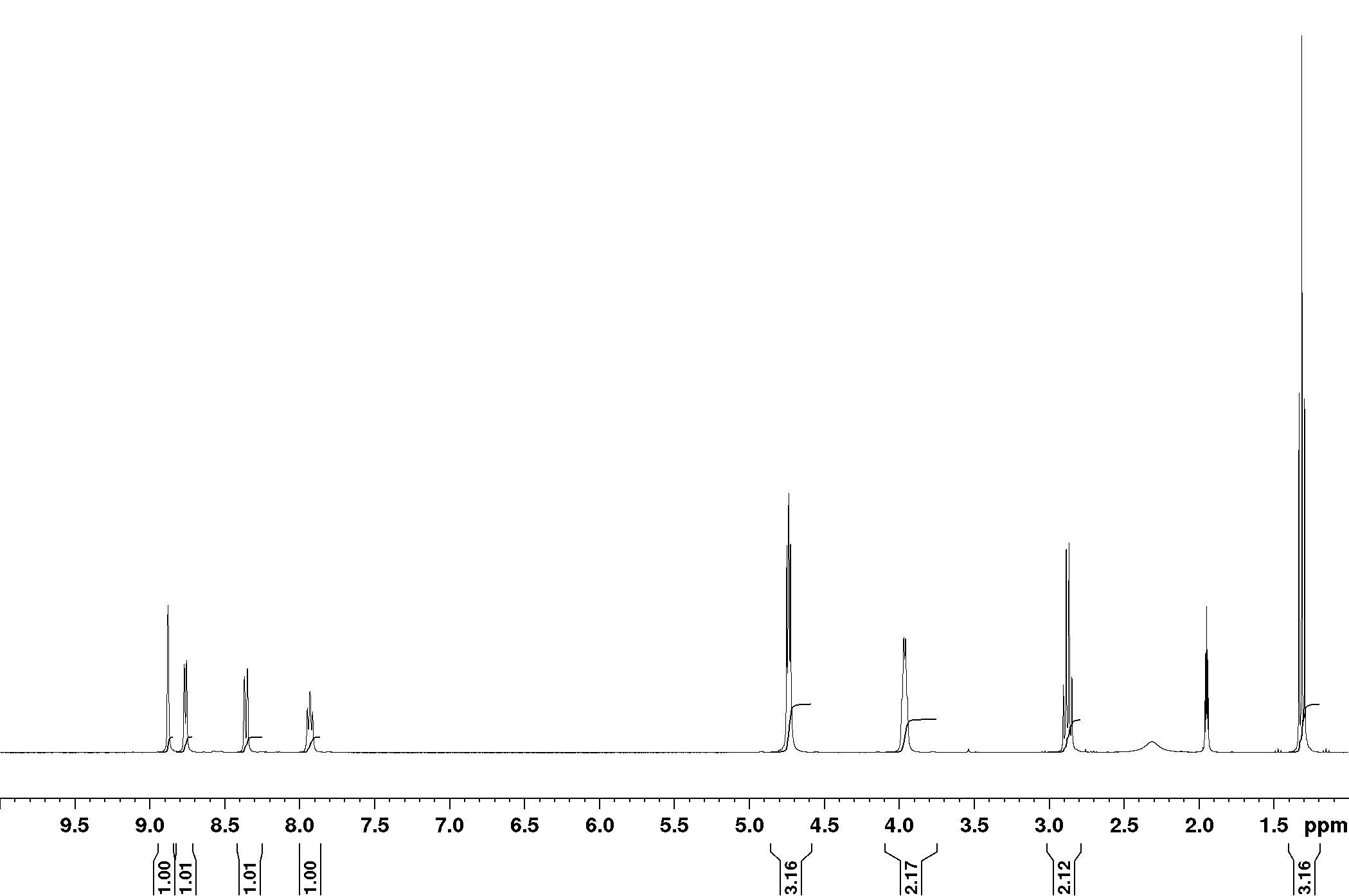


**Figure 19.** ^1^H NMR of 3-ethyl-1-(2-hydroxyethyl)pyridinium bromide, **10Br**


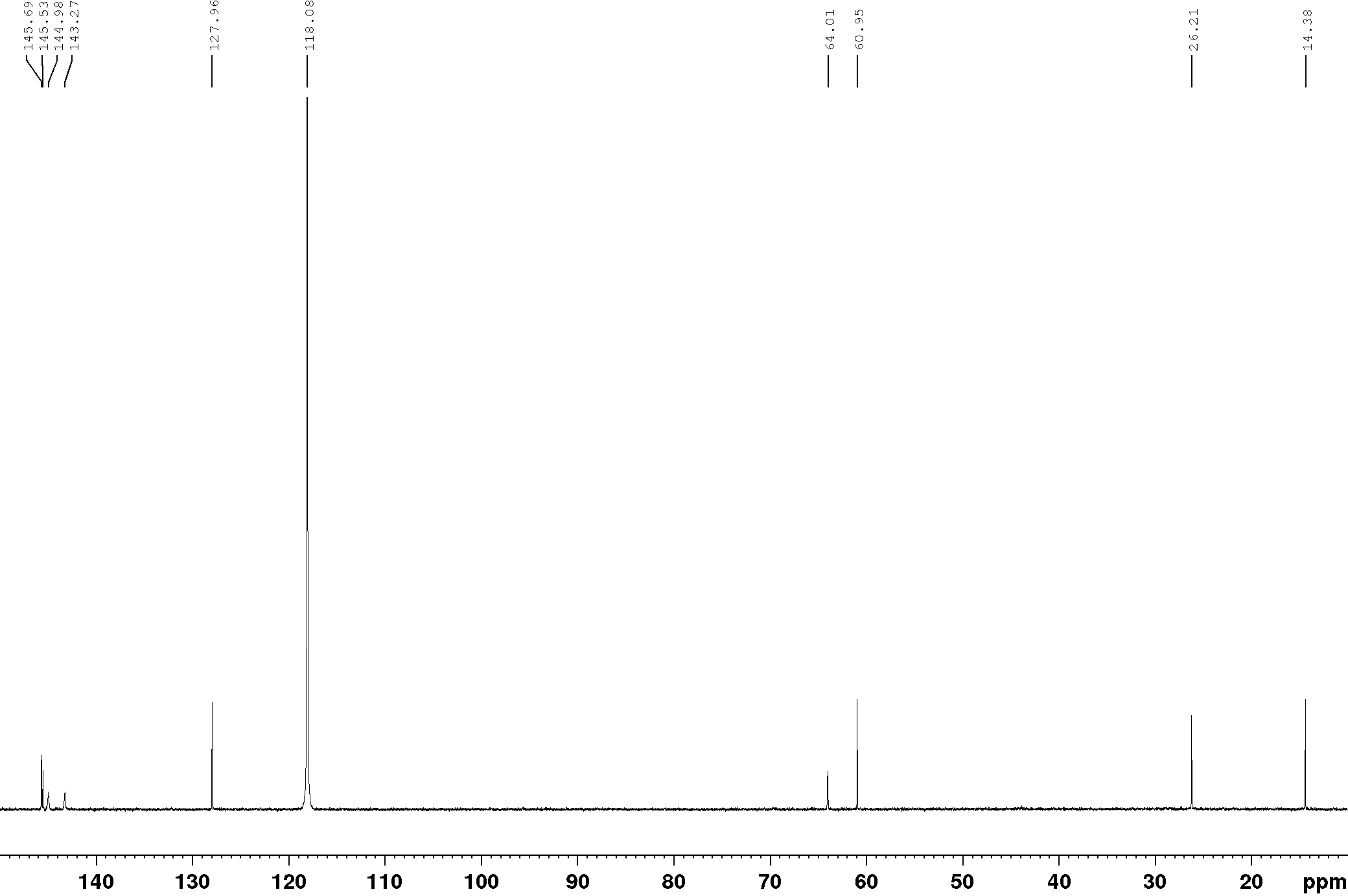


**Figure 20.** ^13^C NMR of 3-ethyl-1-(2-hydroxyethyl)pyridinium bromide, **10Br**


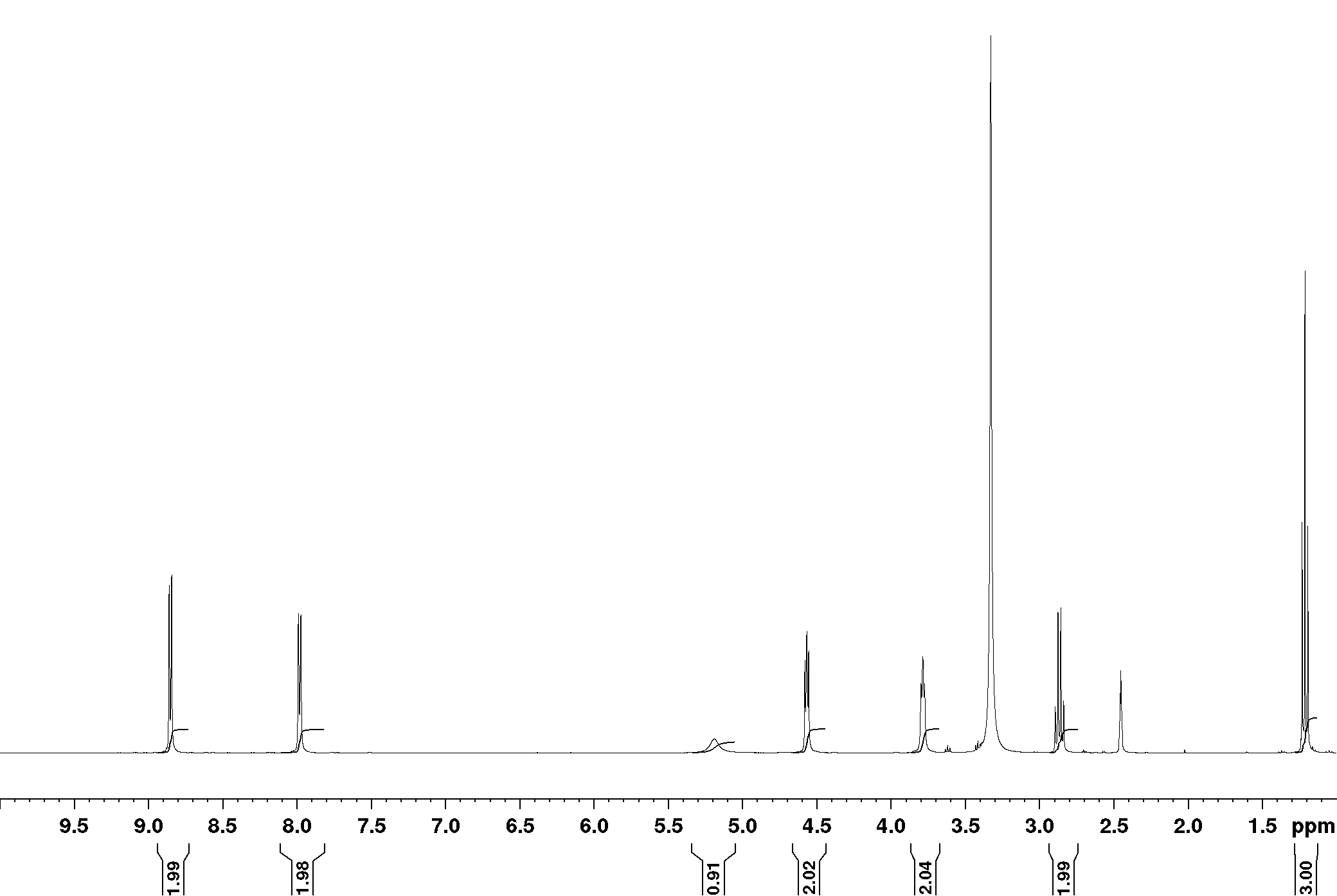


**Figure 21.** ^1^H NMR of 4-ethyl-1-(2-hydroxyethyl)pyridinium bromide, **11Br**


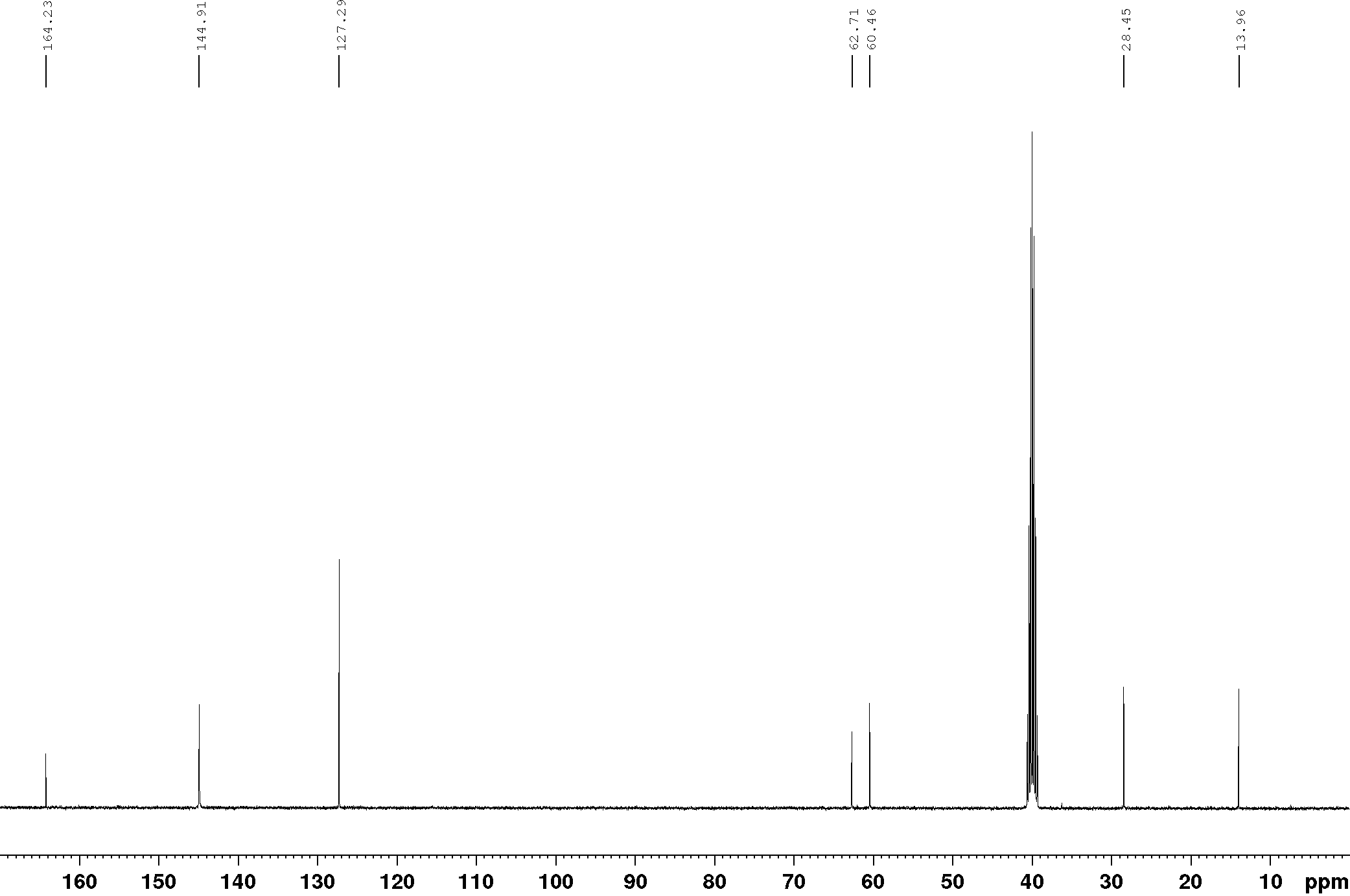


**Figure 22.** ^13^C NMR of 4-ethyl-1-(2-hydroxyethyl)pyridinium bromide, **11Br**


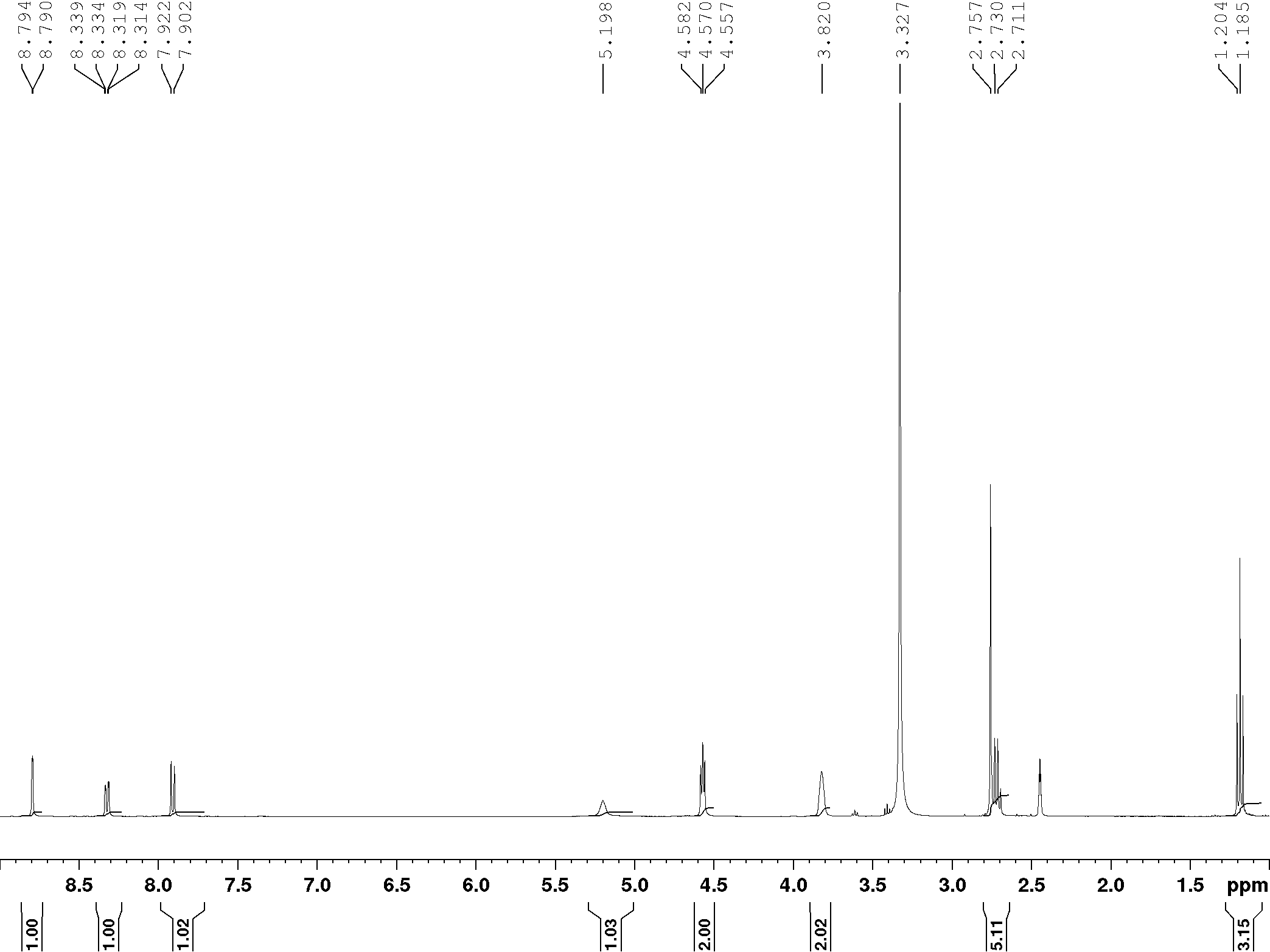


**Figure 23.**^1^H NMR of 5-ethyl-1-(2-hydroxyethyl)-2-methyl pyridinium bromide, **12Br**


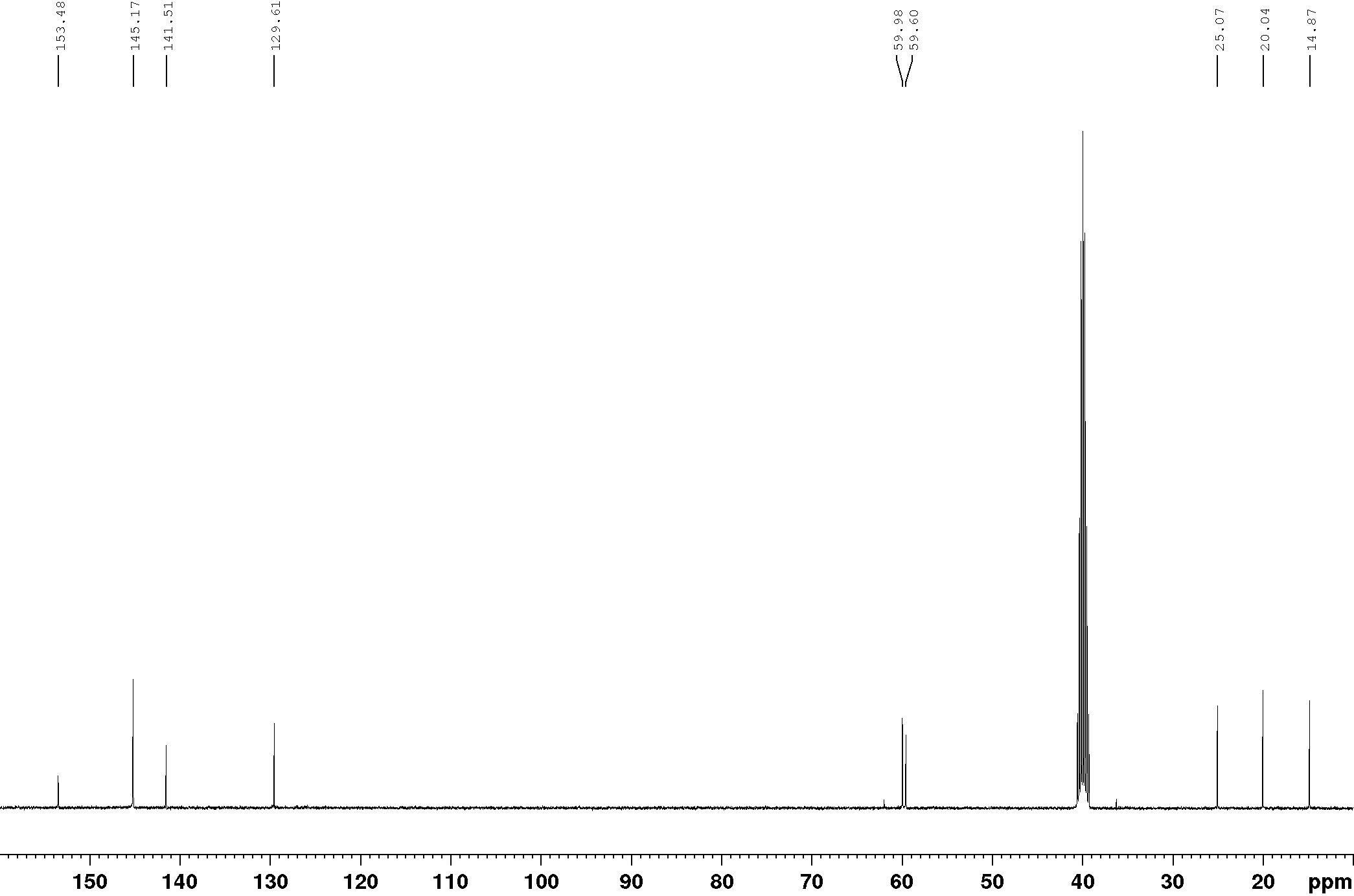


**Figure 24.**^1^H NMR of 5-ethyl-1-(2-hydroxyethyl)-2-methylpyridinium bromide, **12Br**

**b) ^1^H and ^13^C NMR** of ILs; **1-12Tf_2_N**


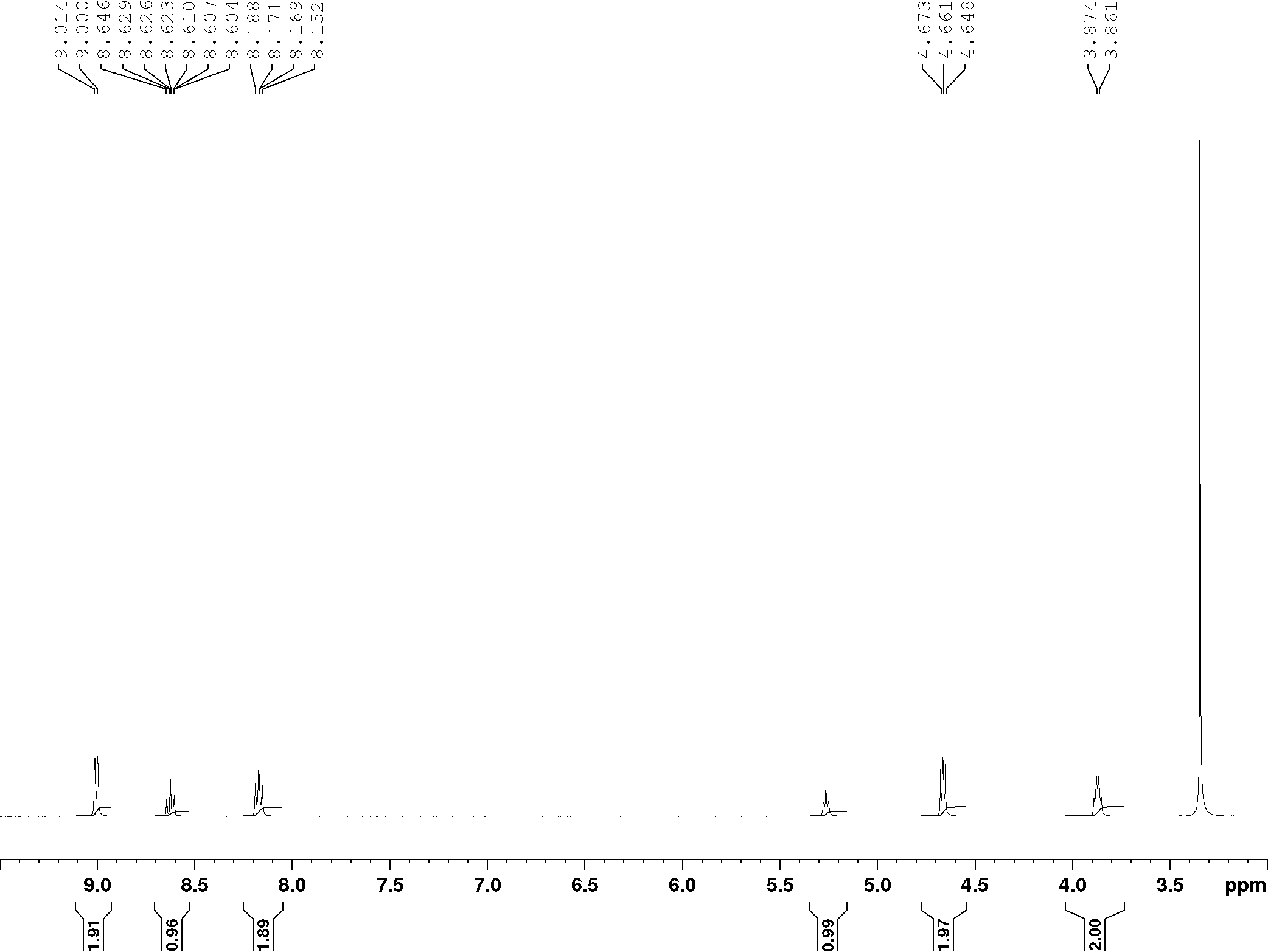


**Figure 25.** ^1^H NMR of 1-(2-hydroxyethyl)pyridinium bis(trifluoromethyl sulfonyl)amide, **1Tf_2_N**


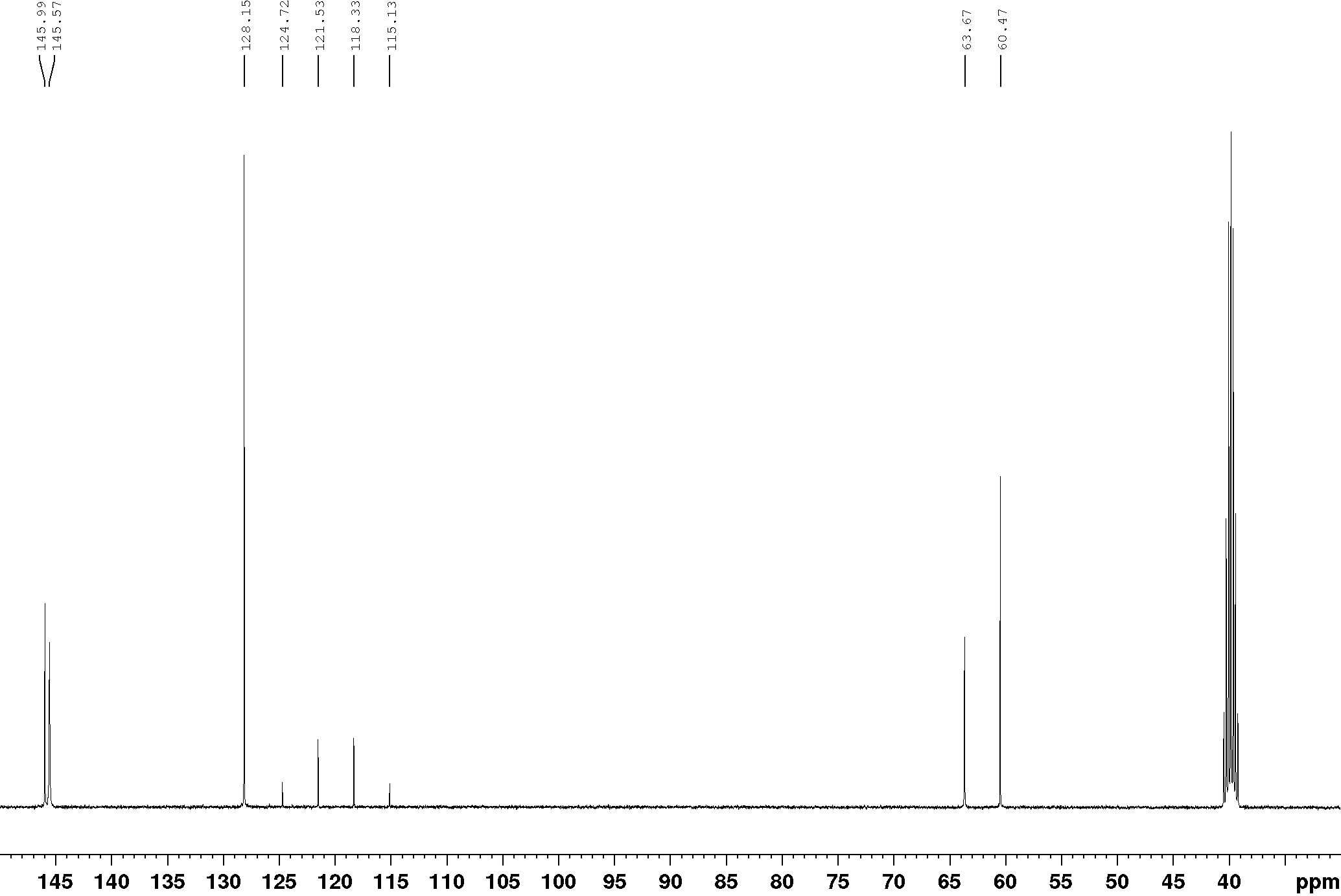


**Figure 26.** ^13^C NMR of 1-(2-hydroxyethyl)pyridinium bis(trifluoromethyl)sulfonyl)amide, **1Tf_2_N**


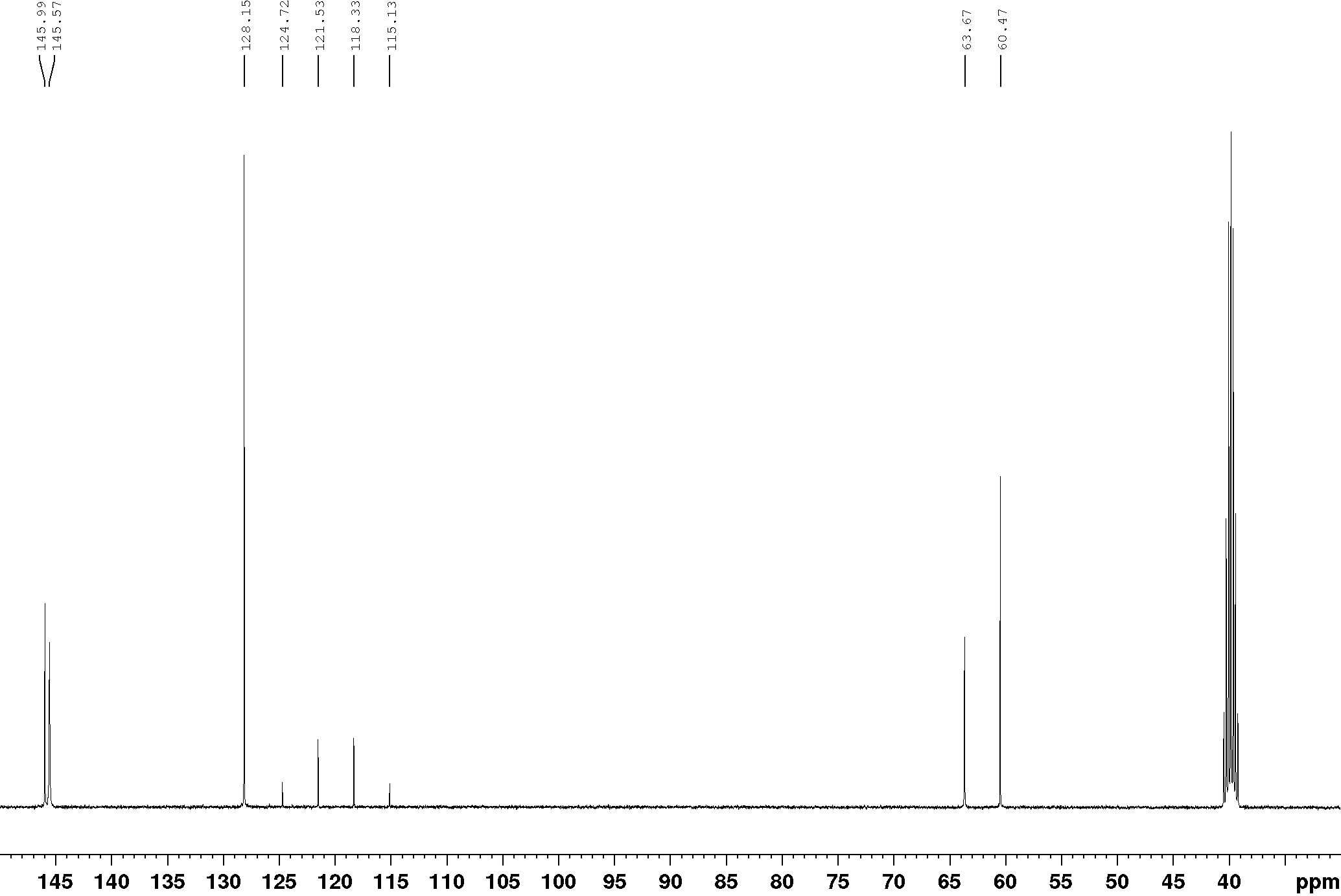


**Figure 27**. ^13^C NMR of 1-(2-hydroxyethyl)-2-methylpyridinium bis(trifluoromethyl sulfonyl)amide, **2Tf_2_N**


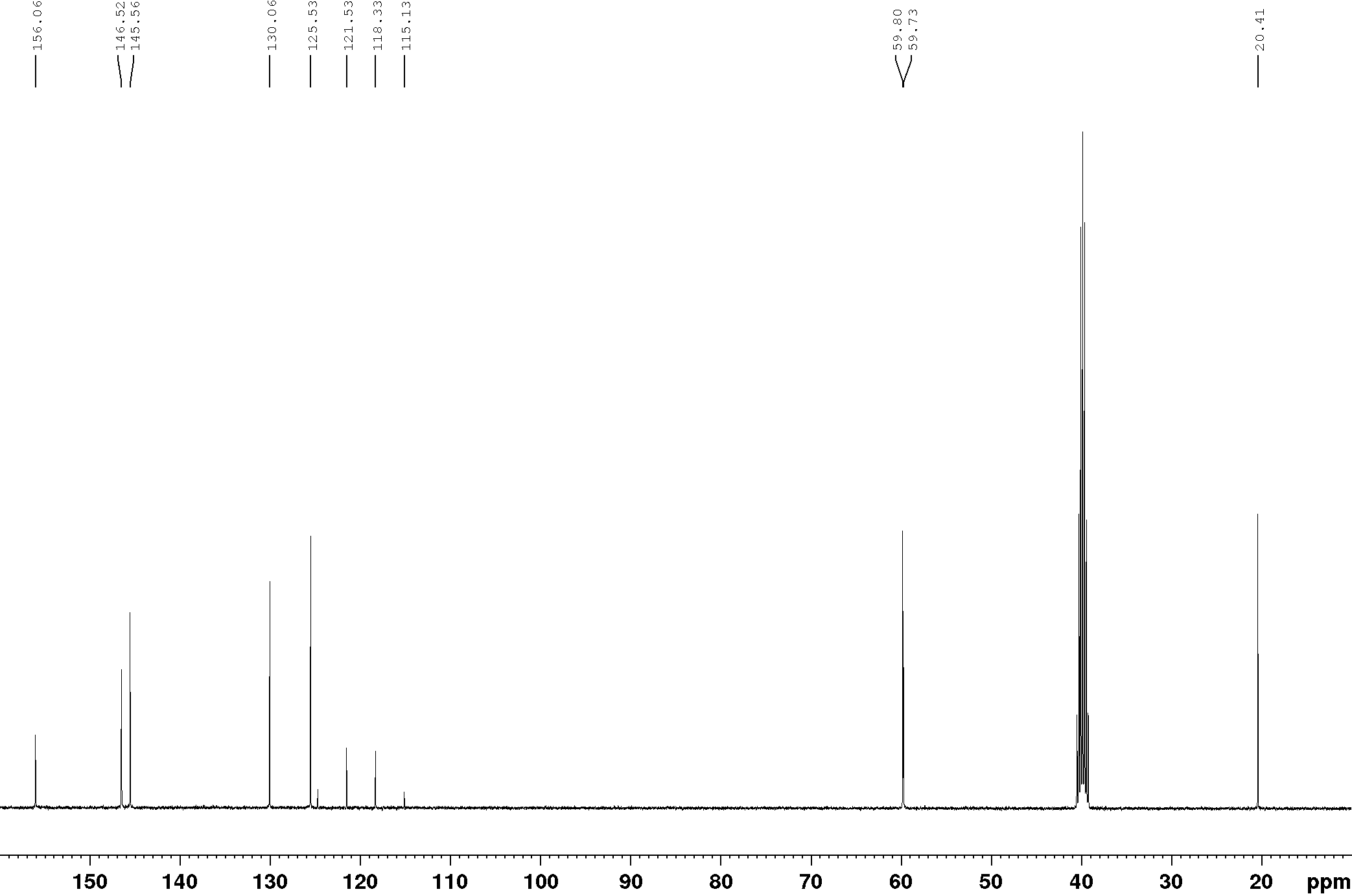


**Figure 27.** ^13^C NMR of 1-(2-hydroxyethyl)-2-methylpyridinium bis(trifluoromethyl sulfonyl) amide**, 2Tf_2_N**


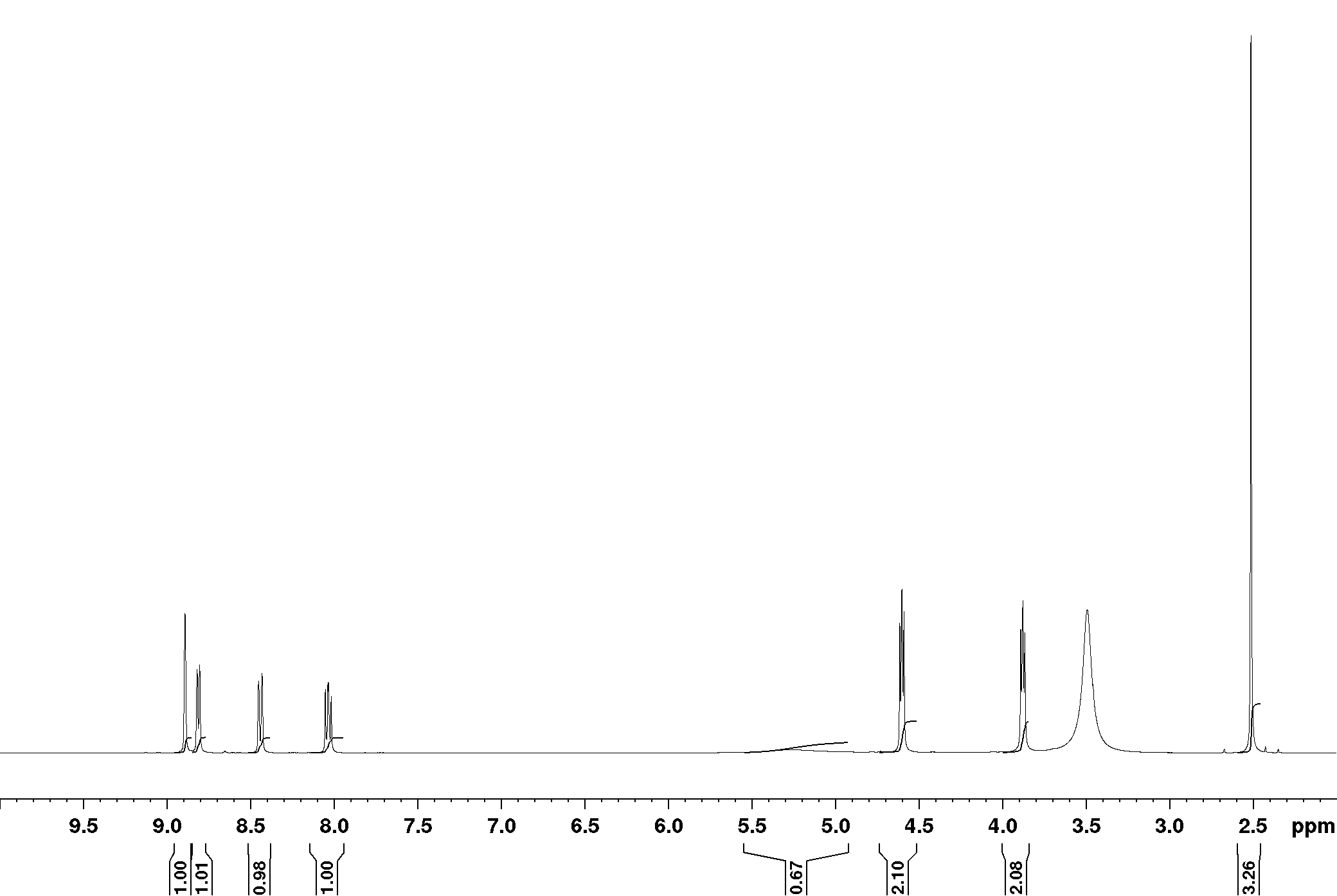


**Figure 28.** ^13^C NMR of 1-(2-hydroxyethyl)-3-methylpyridinium bis (trifluoromethyl sulfonyl)amide, **3Tf_2_N**


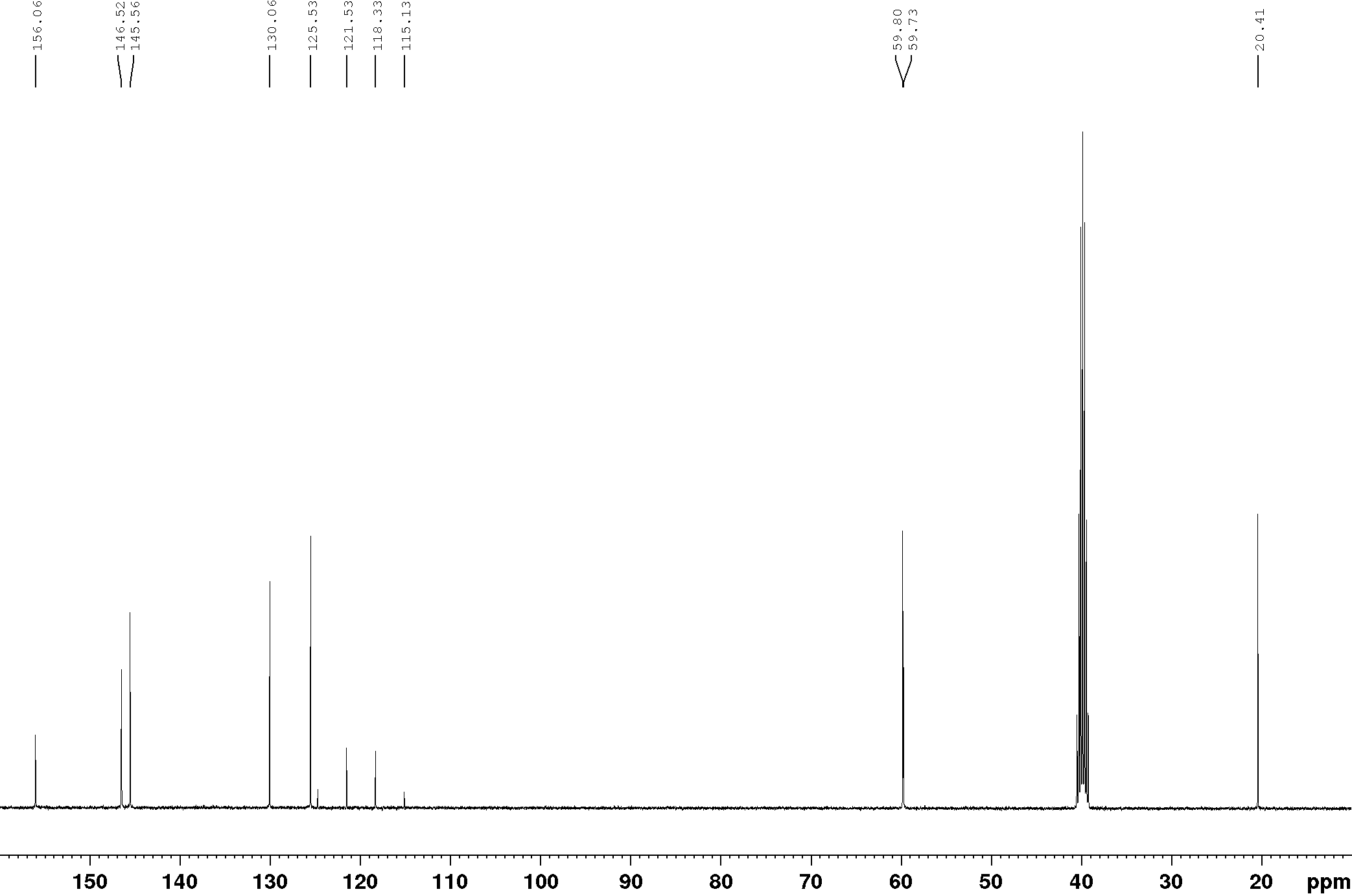


**Figure 29.** ^13^C NMR of 1-(2-hydroxyethyl)-3-methylpyridinium bis(trifluoromethyl sulfonyl)amide, **3Tf_2_N**


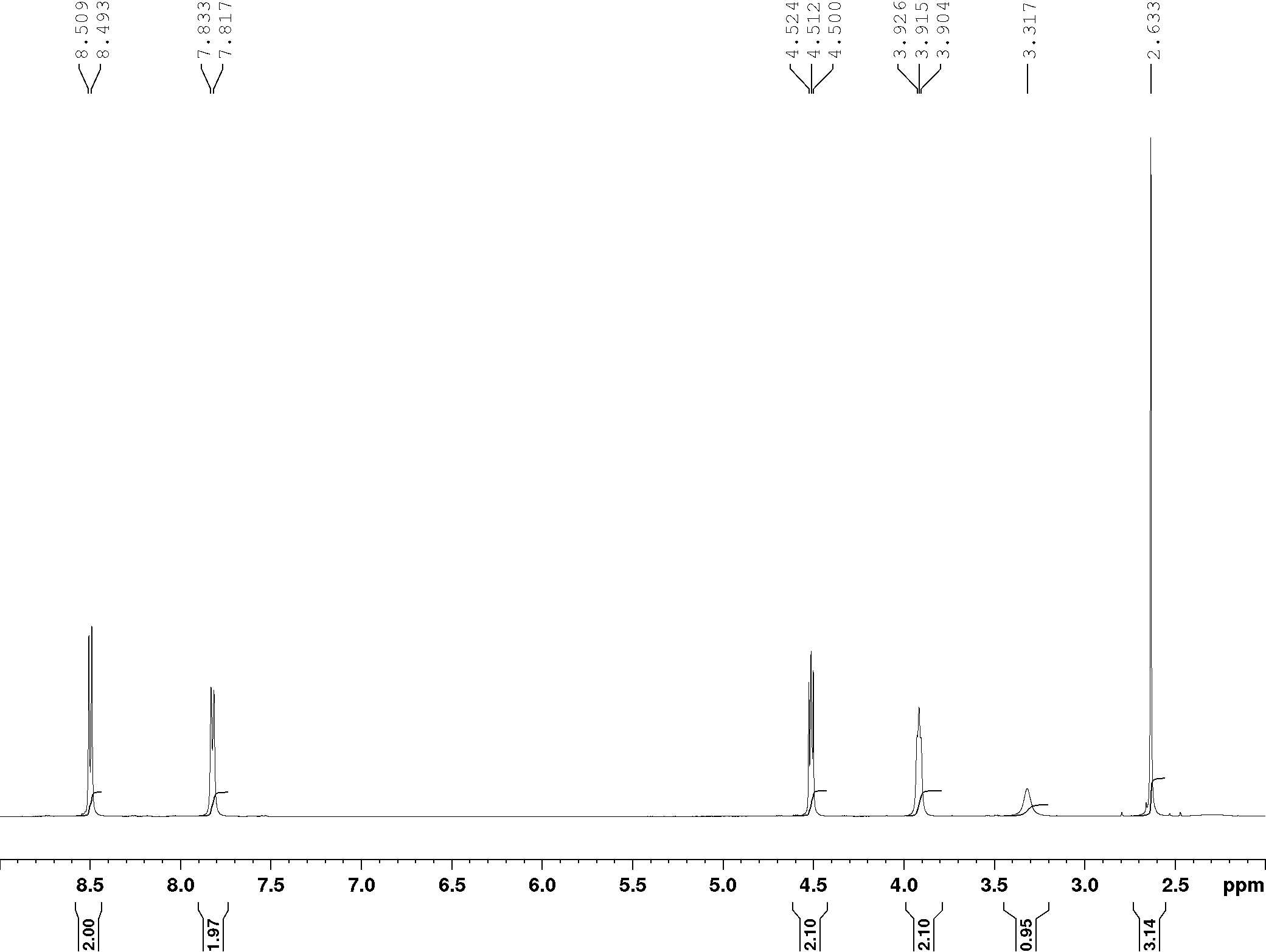


**Figure 30.** ^1^H NMR of 1-(2-hydroxyethyl)-4-methylpyridinium bis(trifluoromethyl sulfonyl)amide, **4Tf_2_N**


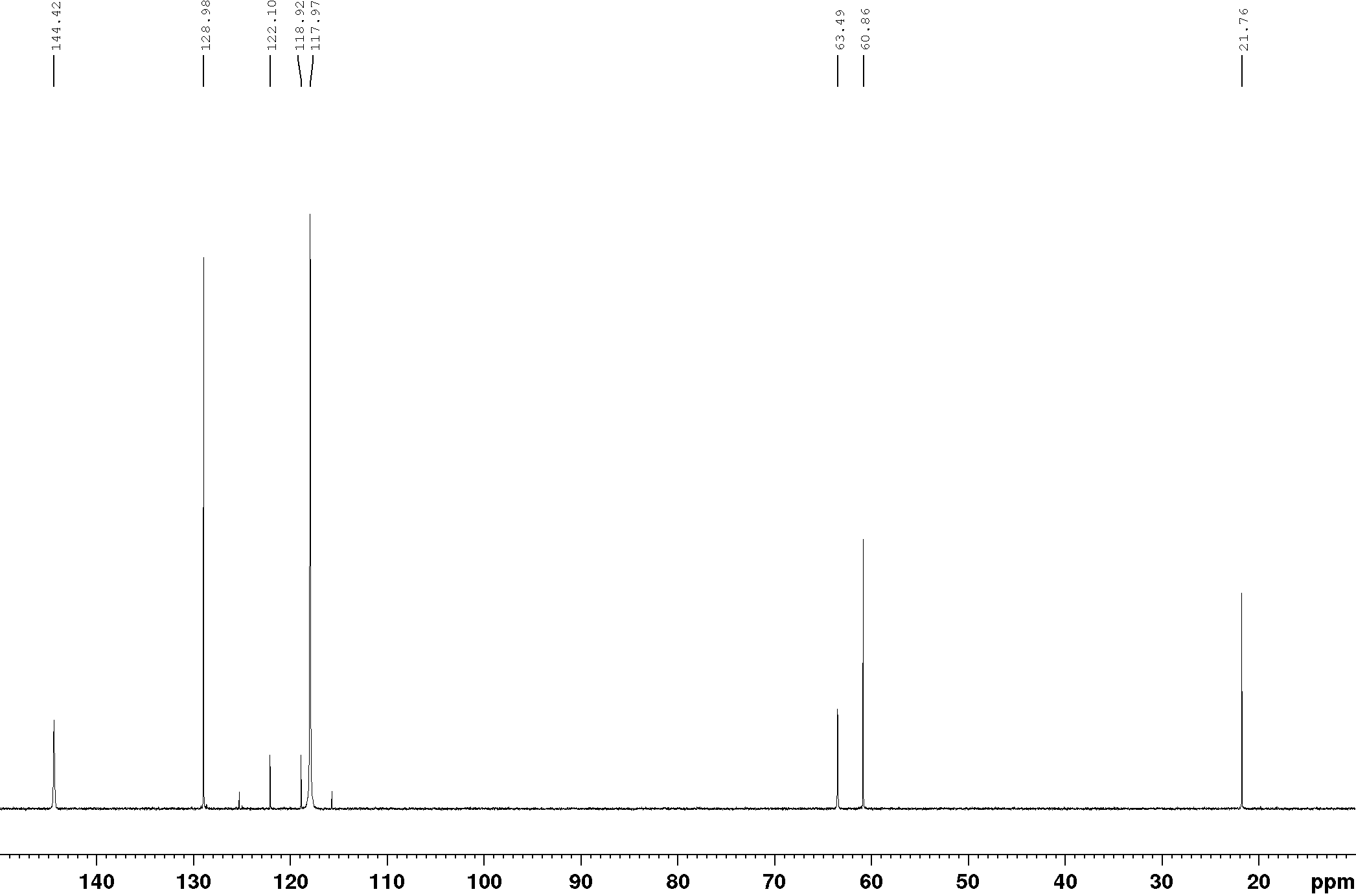


**Figure 31.** ^13^ C NMR of 1-(2-hydroxyethyl)-4-methylpyridinium bis(trifluoromethyl sulfonyl)amide, **4Tf_2_N**


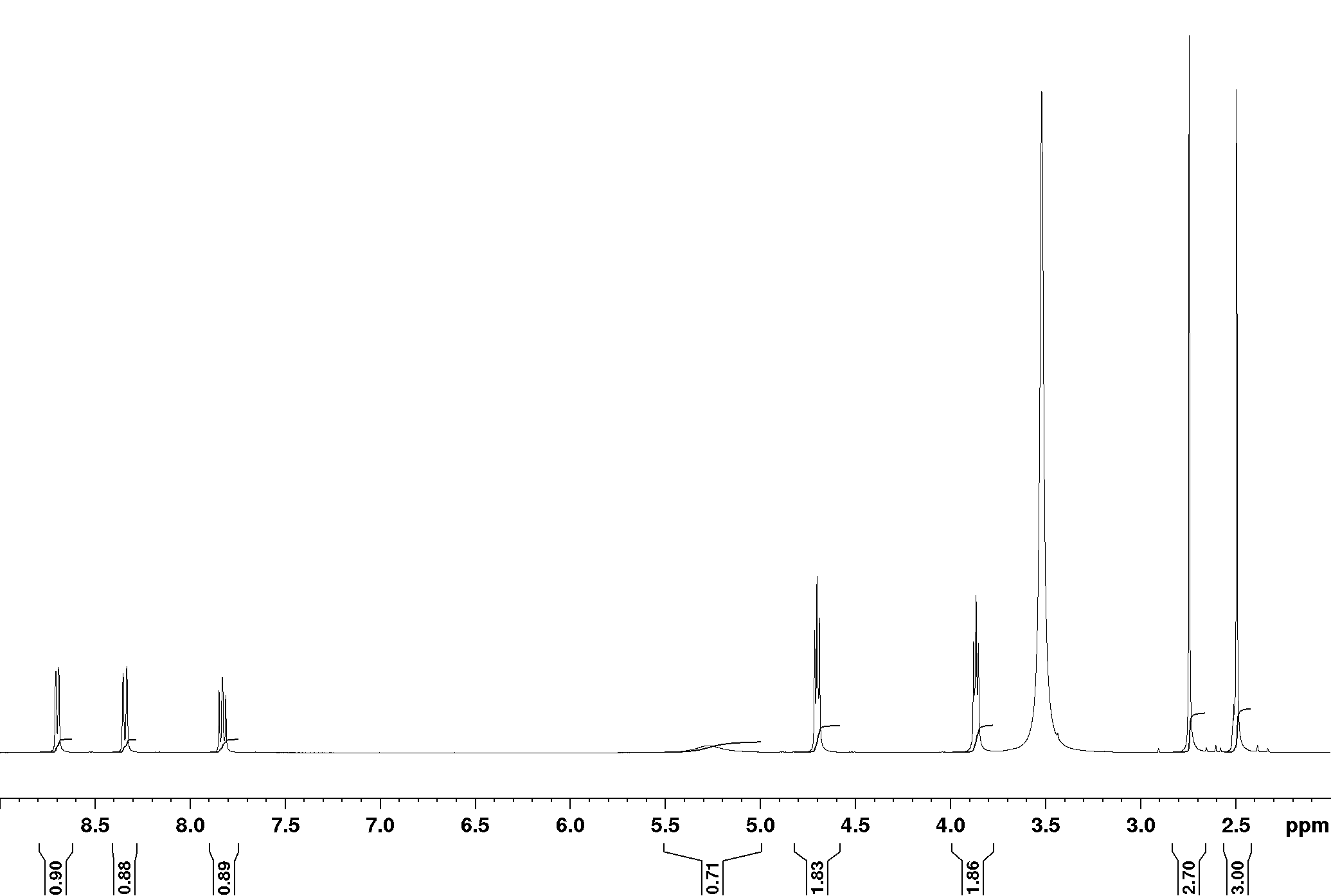


**Figure 32.** ^1^ H NMR of 1-(2-hydroxyethyl)-2,3-dimethylpyridinium bis(trifluoromethyl sulfonyl) amide, **5Tf_2_N**


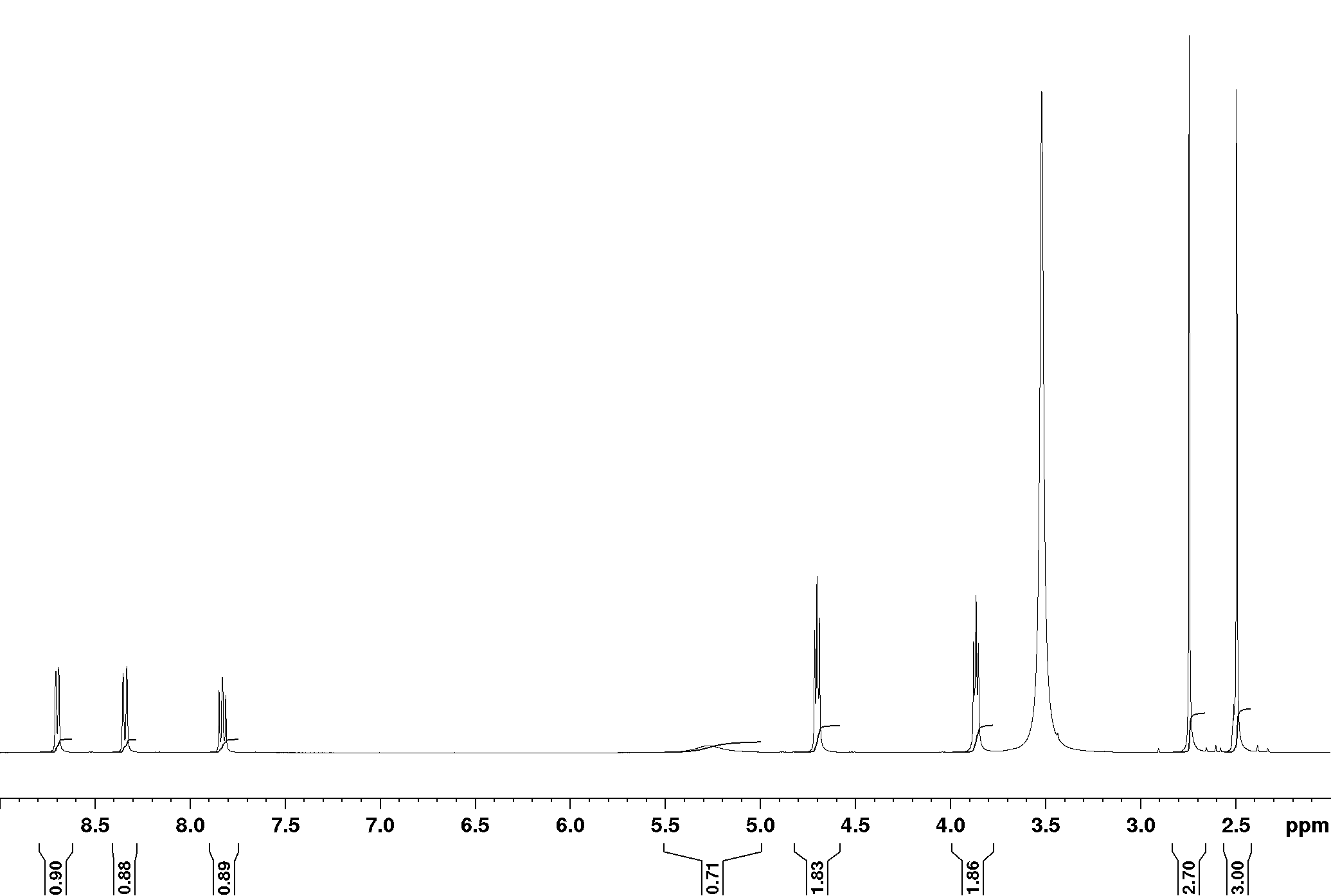


**Figure 33.** ^13^ C NMR of 1-(2-hydroxyethyl)-2,3-dimethylpyridinium bis(trifluoromethyl sulfonyl) amide, **5Tf_2_N**


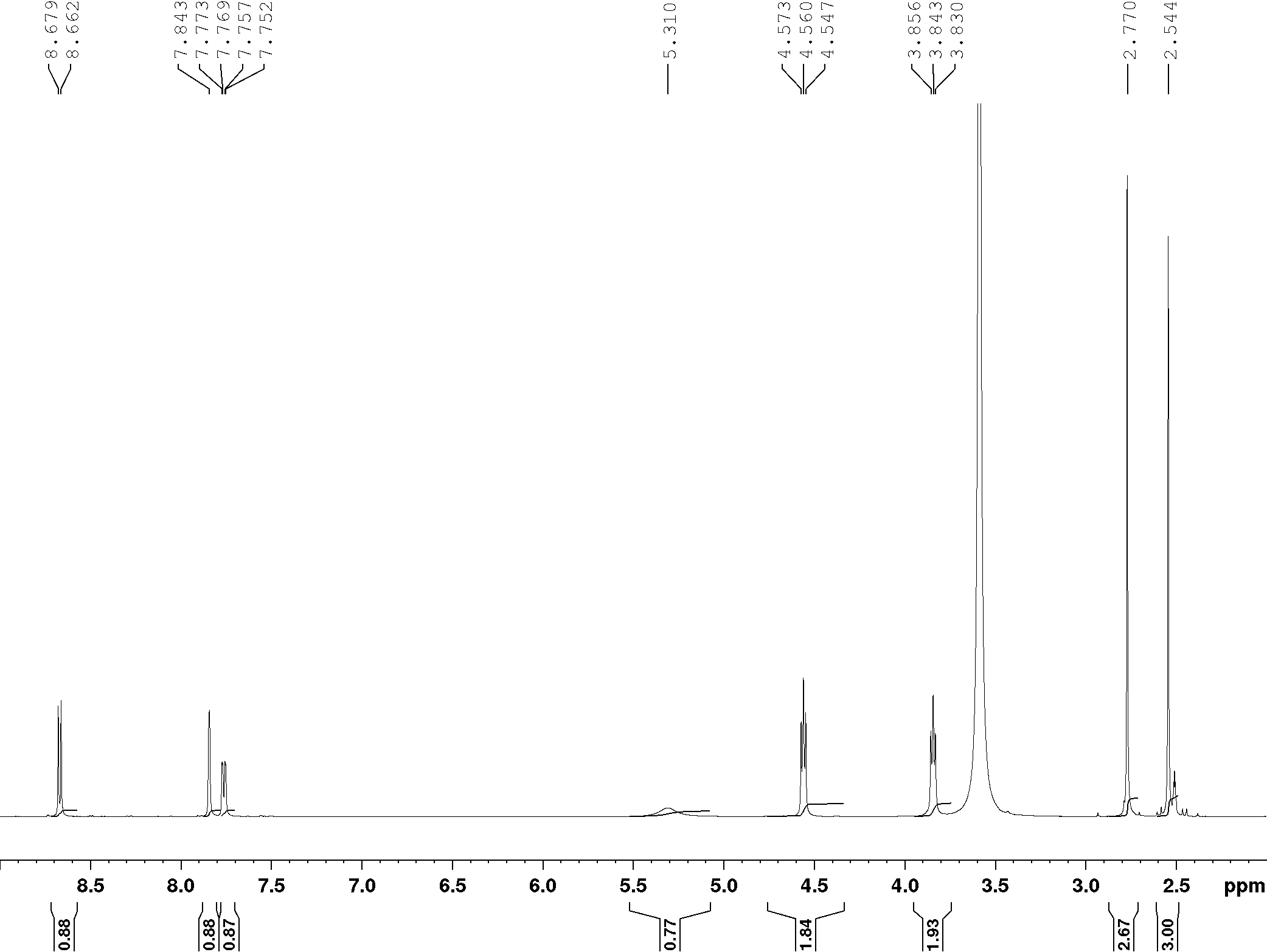


**Figure 32.** ^1^ H NMR of 1-(2-hydroxyethyl)-2,4-dimethylpyridinium bis (trifluoromethyl sulfonyl) amide, **6Tf_2_N**


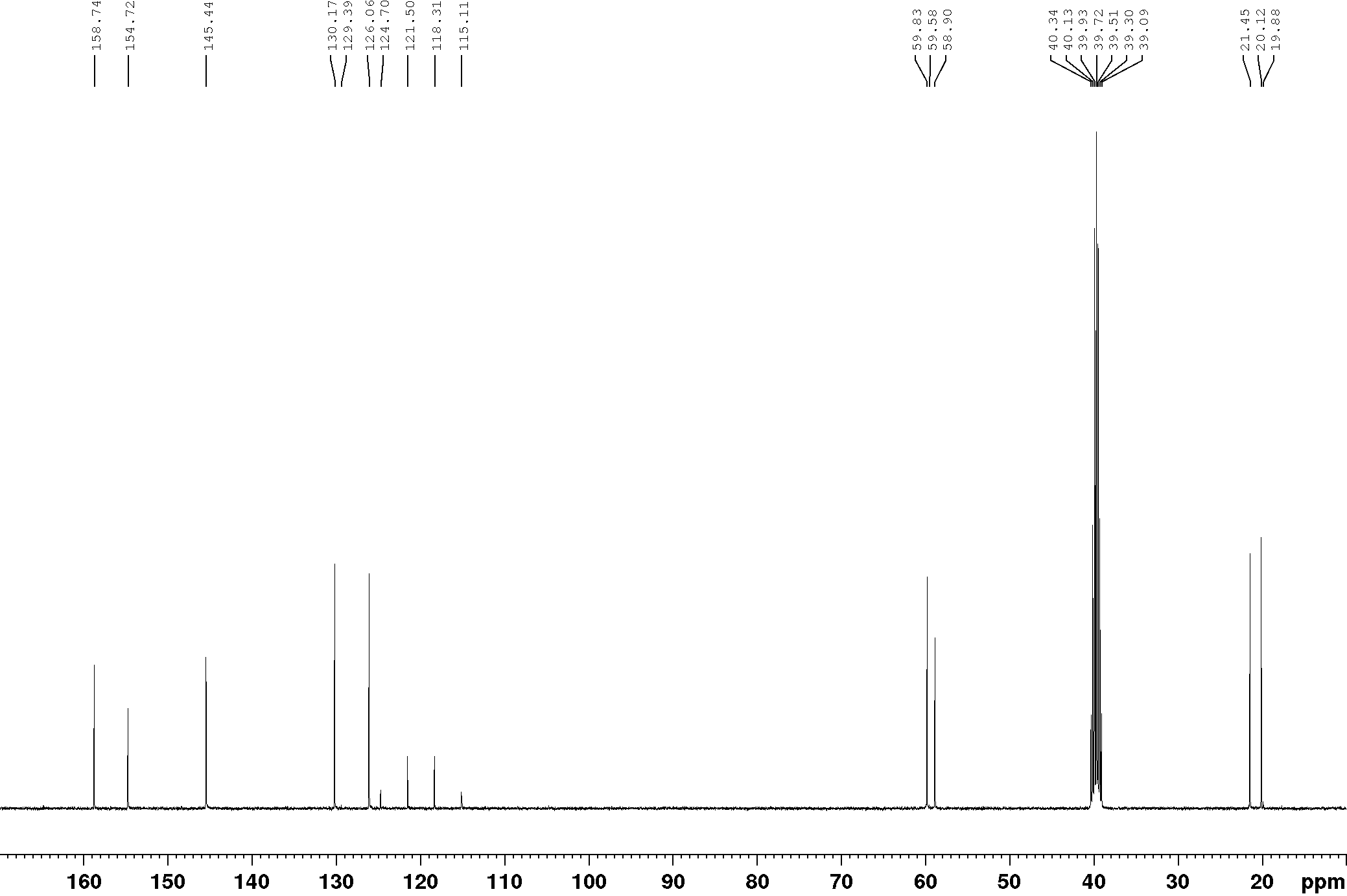


**Figure 33.** ^13^ C NMR of 1-(2-hydroxyethyl)-2,4-dimethylpyridinium bis (trifluoromethyl sulfonyl) amide, **6Tf_2_N**


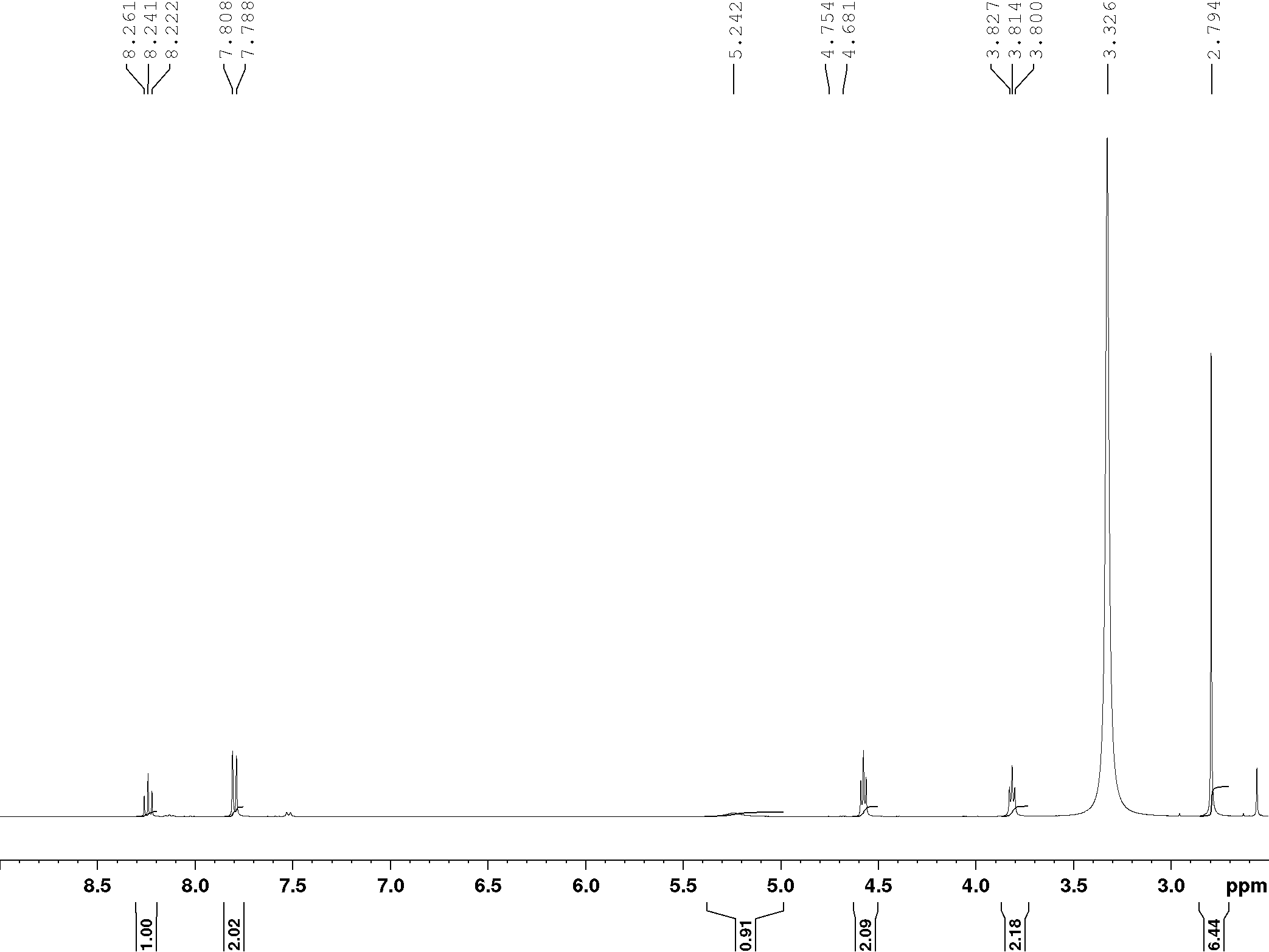


**Figure 34.** ^1^ H NMR of 1-(2-hydroxyethyl)-2,6-dimethylpyridinium bis(trifluoromethyl sulfonyl) amide, **7Tf_2_N**


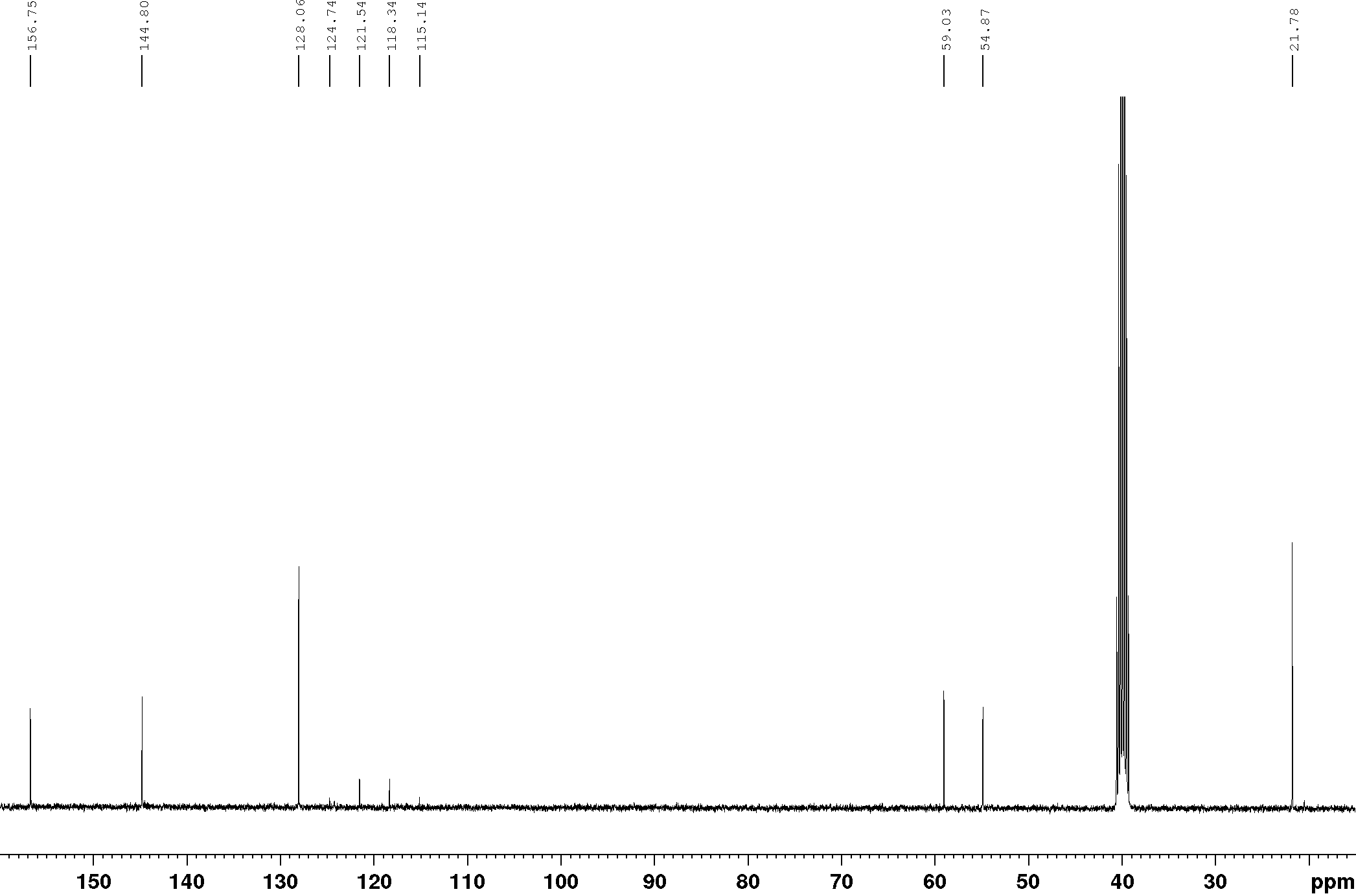


**Figure 34.** ^13^ C NMR of 1-(2-hydroxyethyl)-2,6-dimethylpyridinium bis(trifluoromethyl sulfonyl) amide, **7Tf_2_N**

**
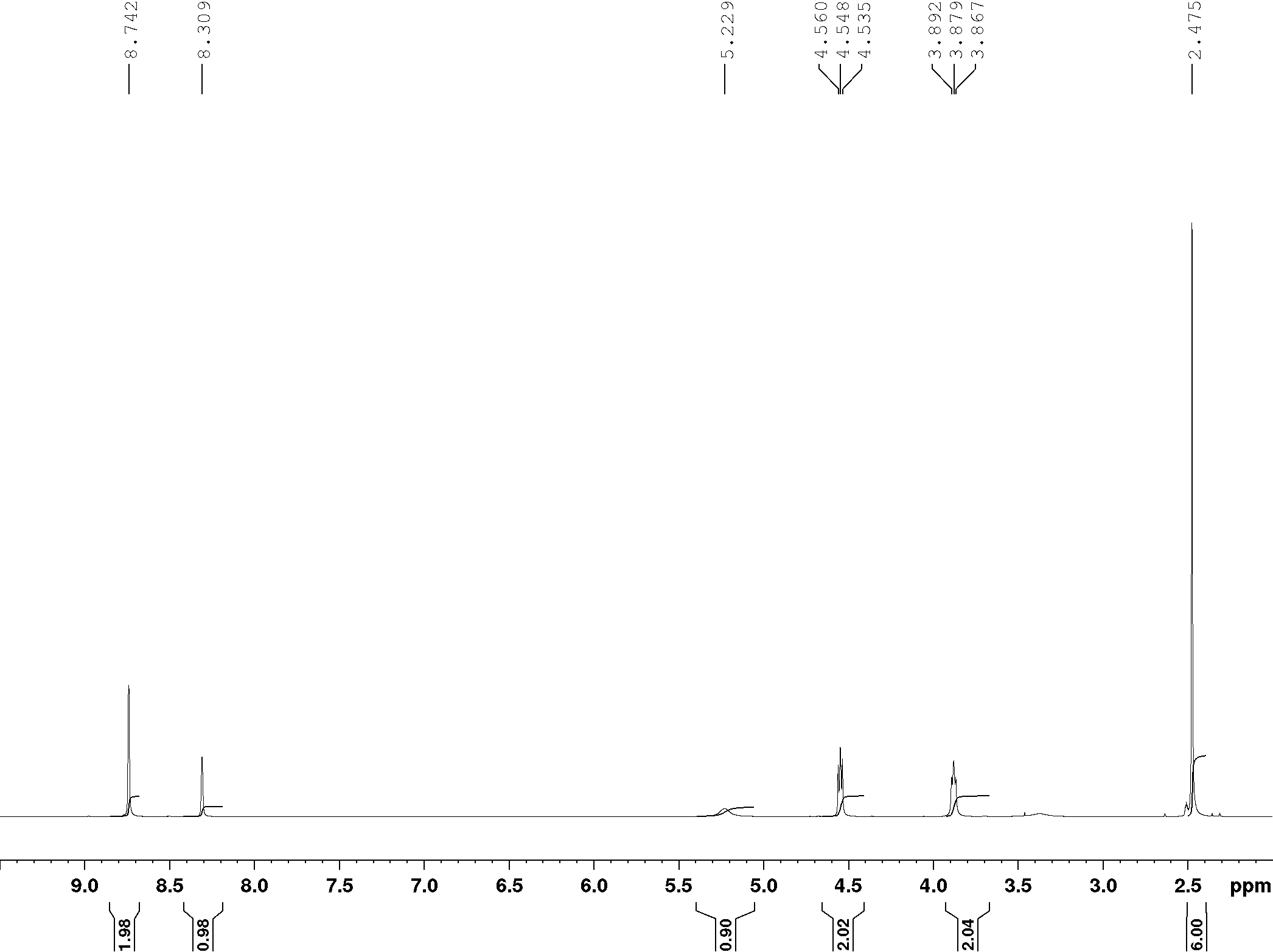
**

**Figure 35.** ^1^H NMR of 1-(2-hydroxyethyl)-3, 5-dimethylpyridinium bis(trifluoromethyl sulfonyl) amide, **8Tf_2_N**

**
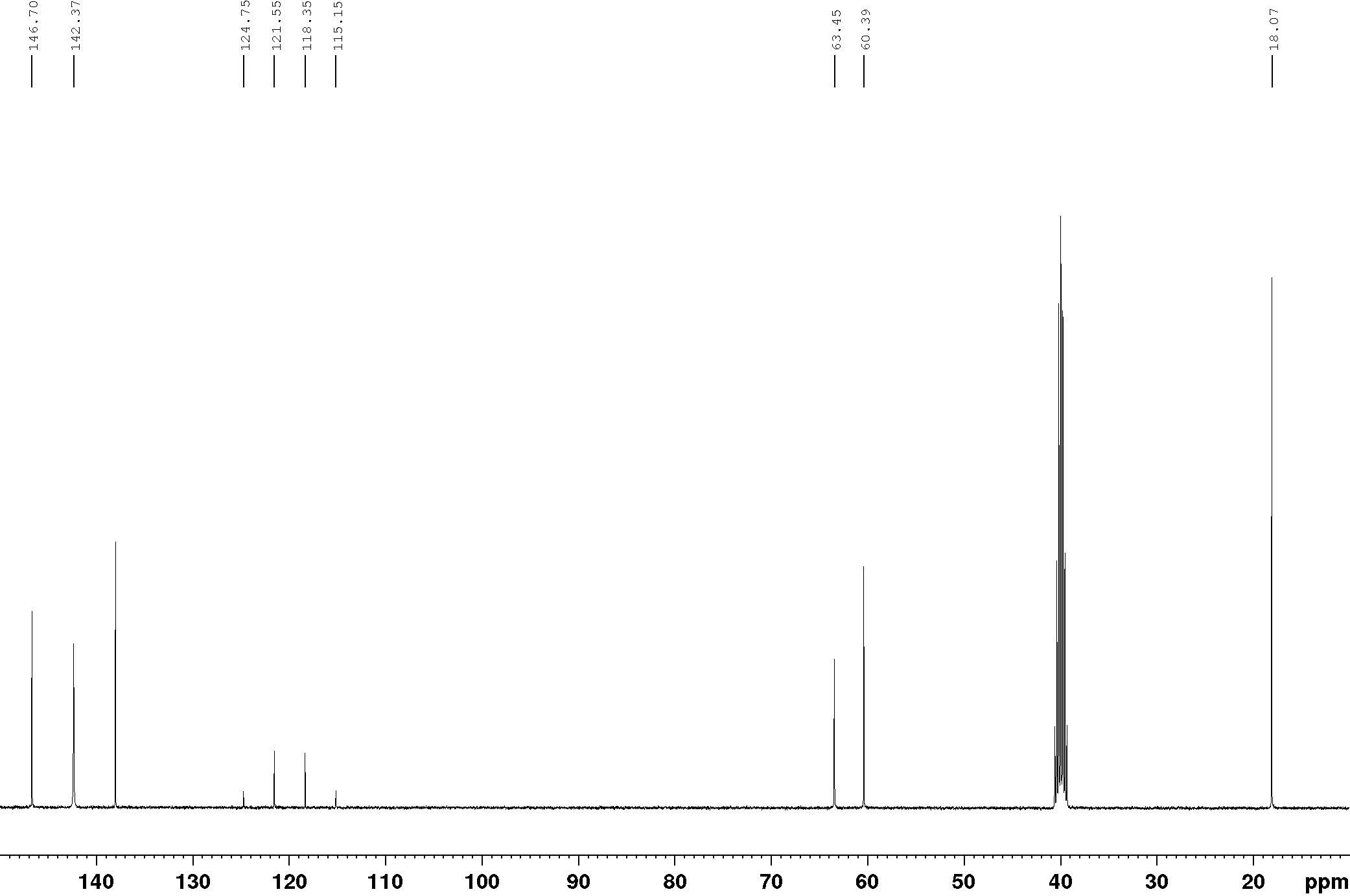
**

**Figure 36.** ^13^C NMR of 1-(2-hydroxyethyl)-3,5-dimethylpyridinium bis(trifluoromethylsulfonyl) amide, **8Tf_2_N**


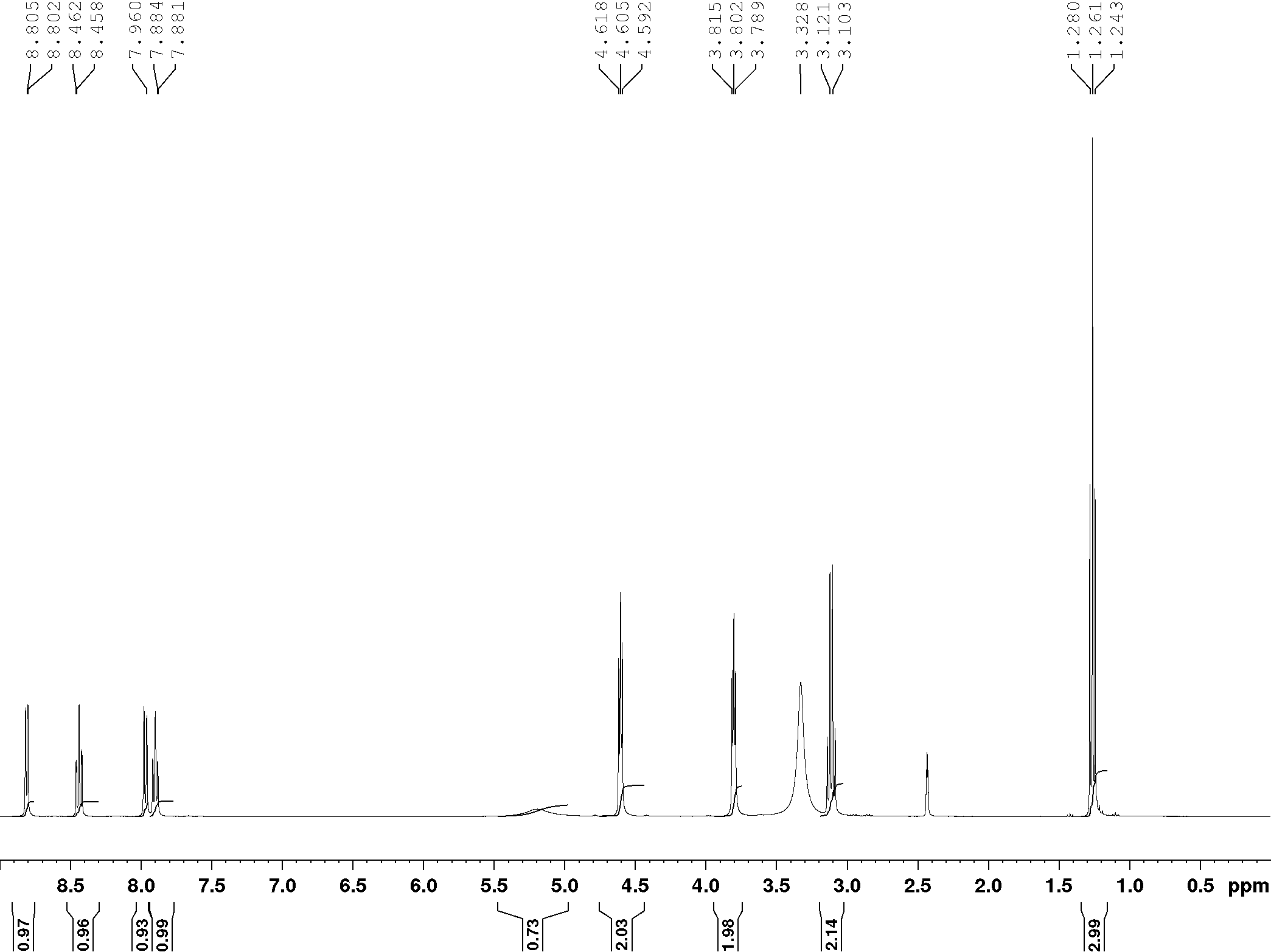


**Figure 37.** ^1^H NMR of 2-ethyl-1-(2-hydroxyethyl)pyridinium bis(trifluoromethyl)sulfonyl) amide, **9Tf_2_N**


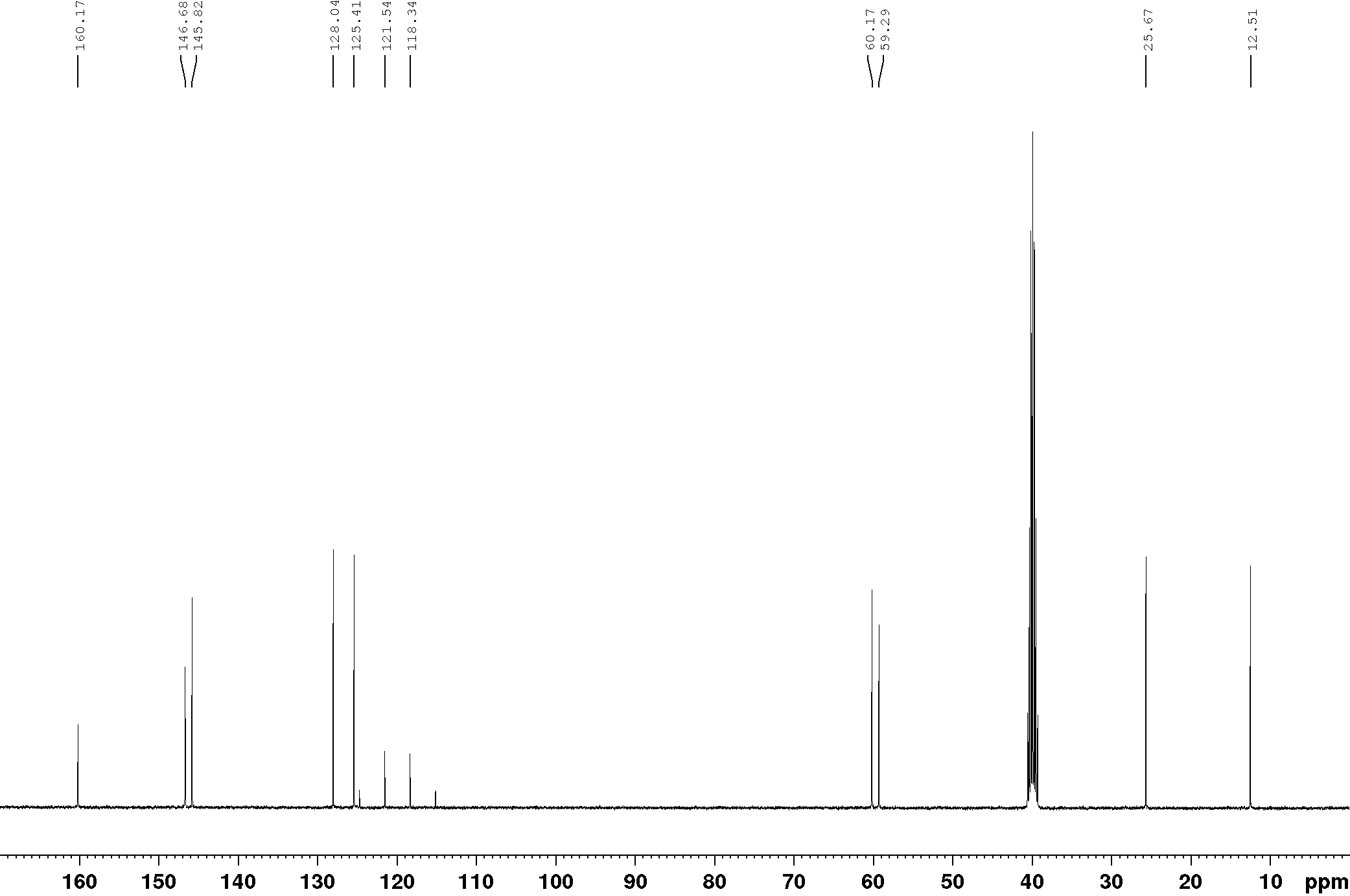


**Figure 38.** ^13^C NMR of 2-ethyl-1-(2-hydroxyethyl)pyridinium bis(trifluoromethylsulfonyl) amide, **9Tf_2_N**


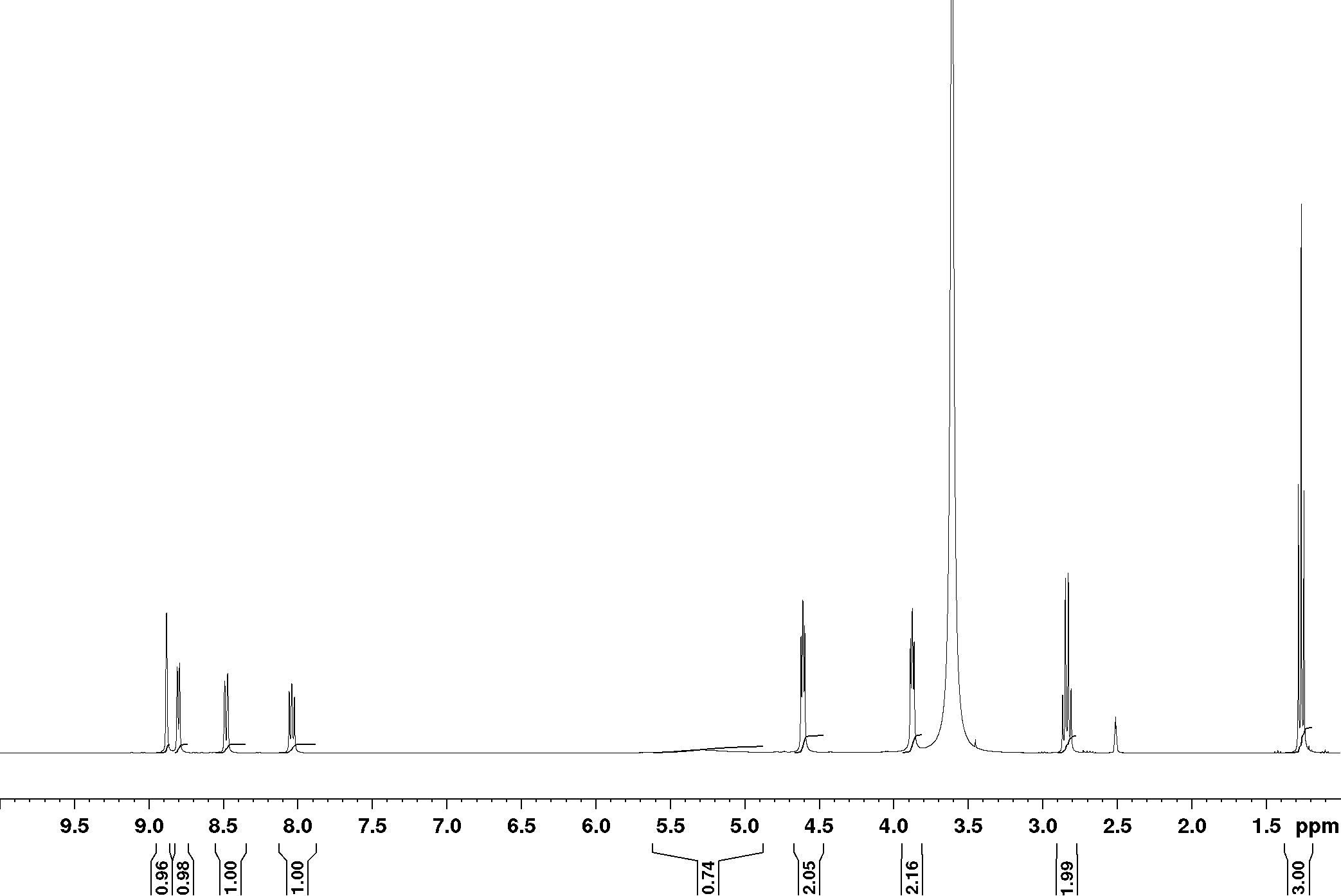


**Figure 39.** ^1^H NMR of 3-ethyl-1-(2-hydroxyethyl)pyridinium bis(trifluoromethyl sulfonyl) amide, **10Tf_2_N**


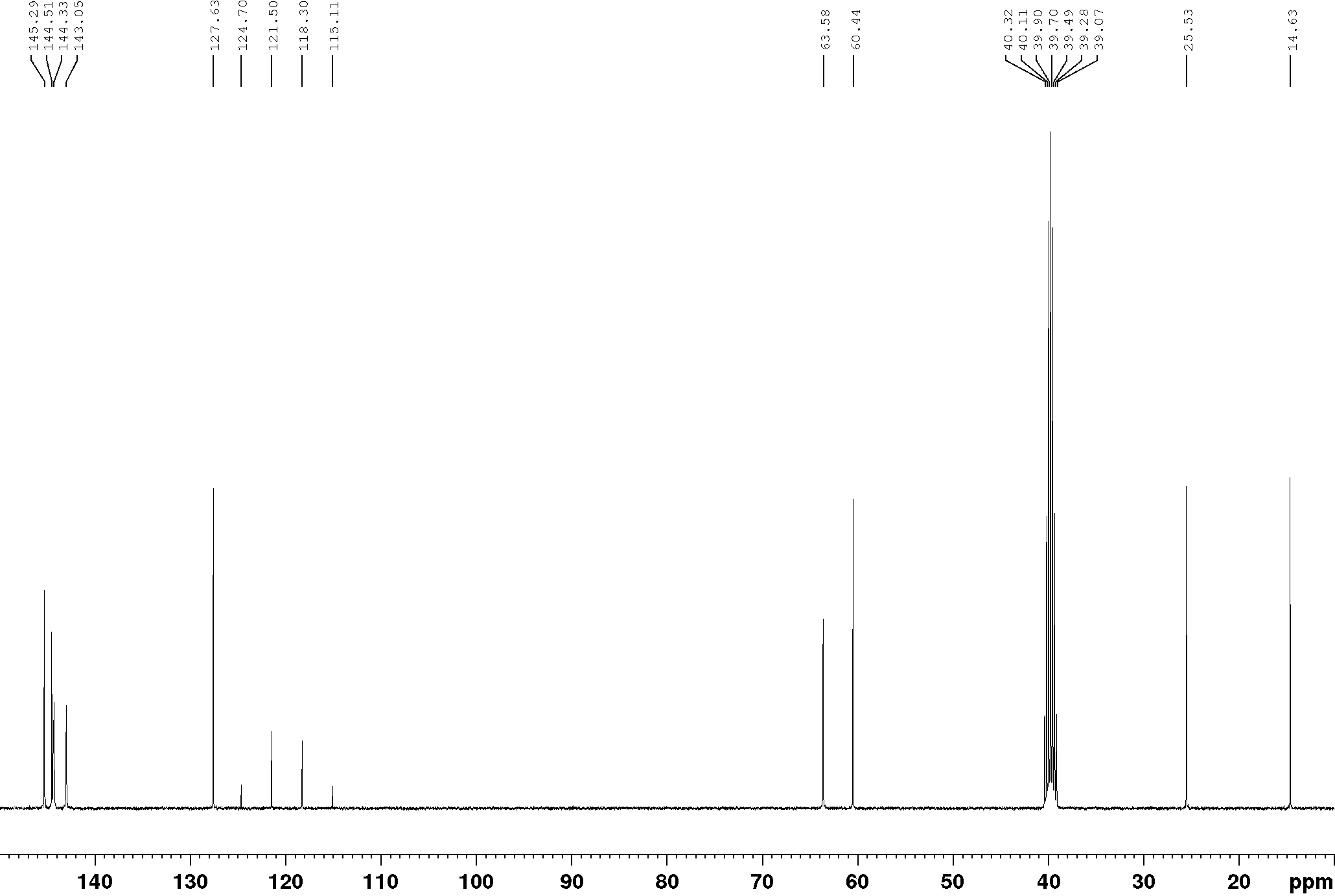


**Figure 40.** ^13^C NMR of 3-ethyl-1-(2-hydroxyethyl)pyridinium bis(trifluoromethyl sulfonyl) amide, **10Tf_2_N**


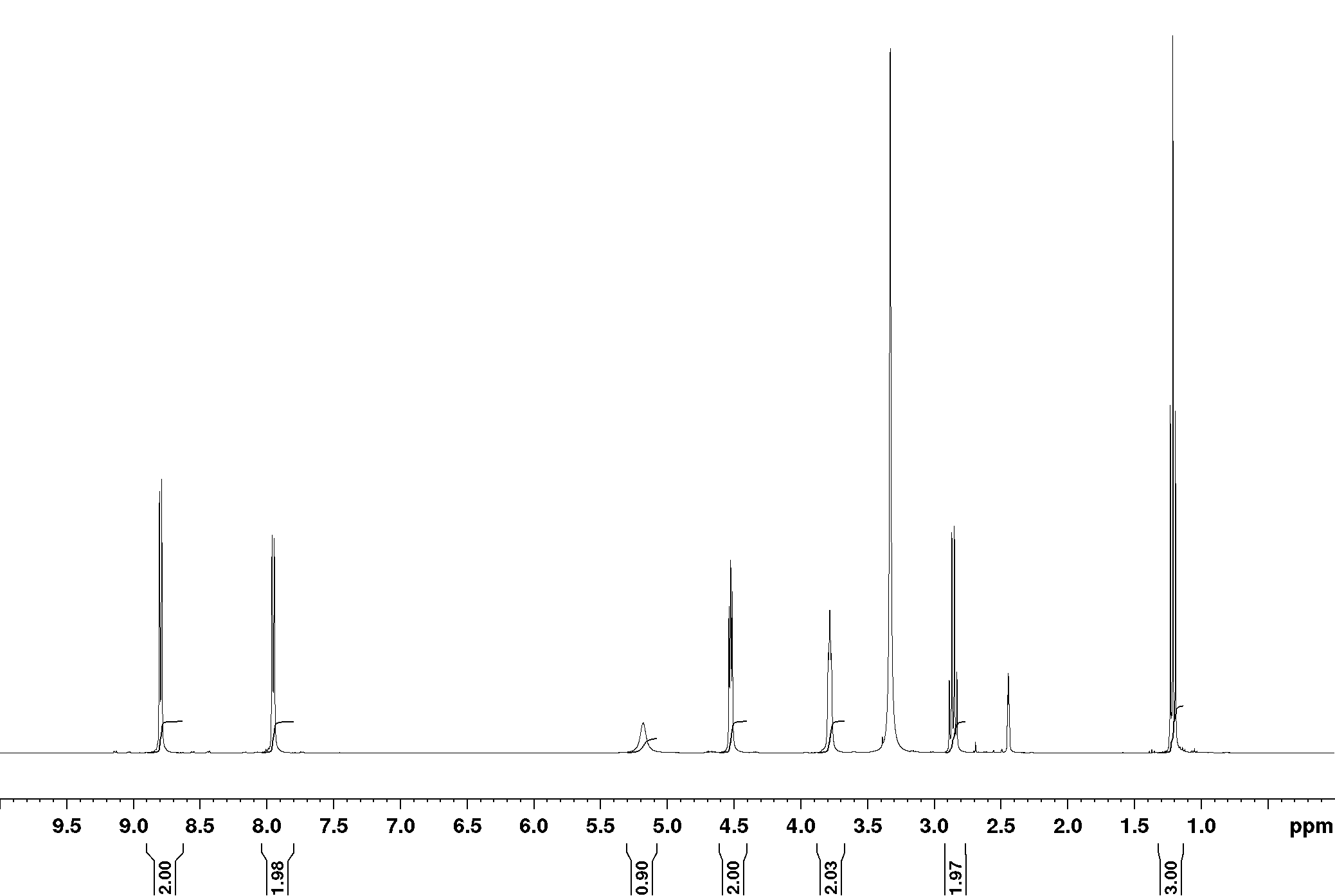


**Figure 41.** ^1^H NMR of 4-ethyl-1-(2-hydroxyethyl)pyridinium bis(trifluoromethyl sulfonyl) amide, **11Tf_2_N**


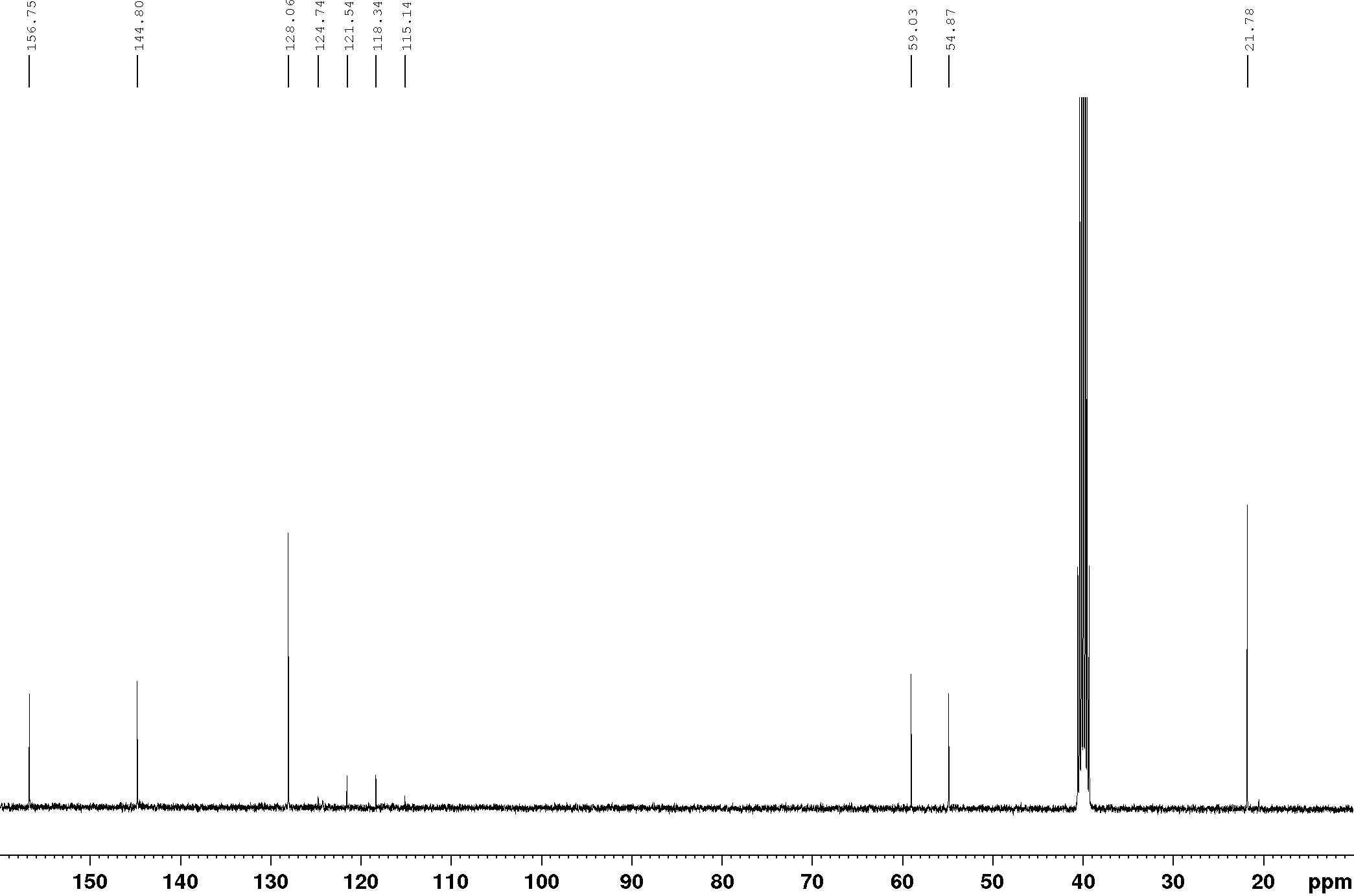


**Figure 42.** ^13^C NMR of 4-ethyl-1-(2-hydroxyethyl)pyridinium bis(trifluoromethyl sulfonyl) amide, **11Tf_2_N**


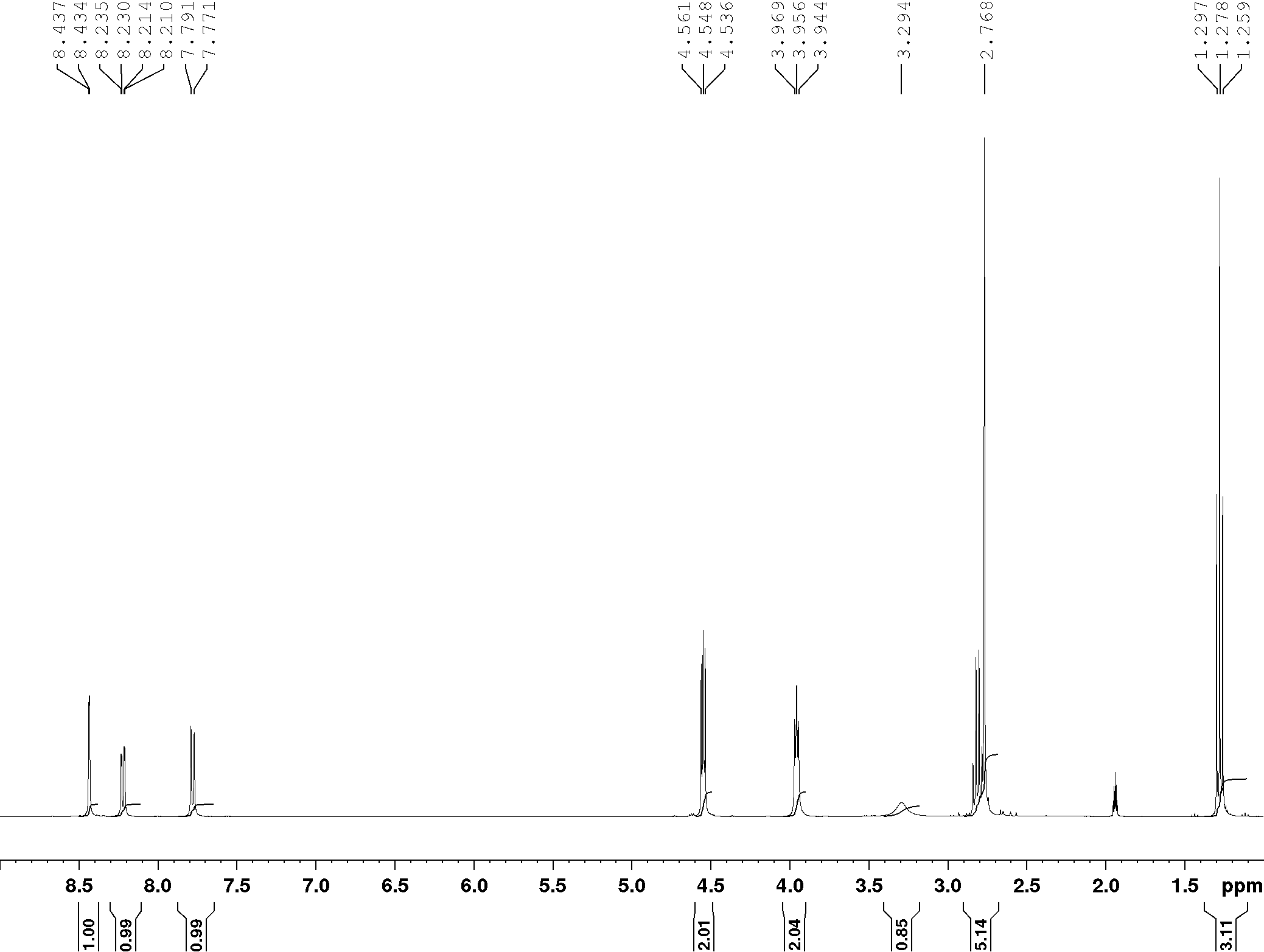


**Figure 43.** ^1^H NMR of 5-ethyl-1-(2-hydroxyethyl)-2-methylpyridinium bis(trifluoromethyl sulfonyl)amide, **12Tf_2_N**


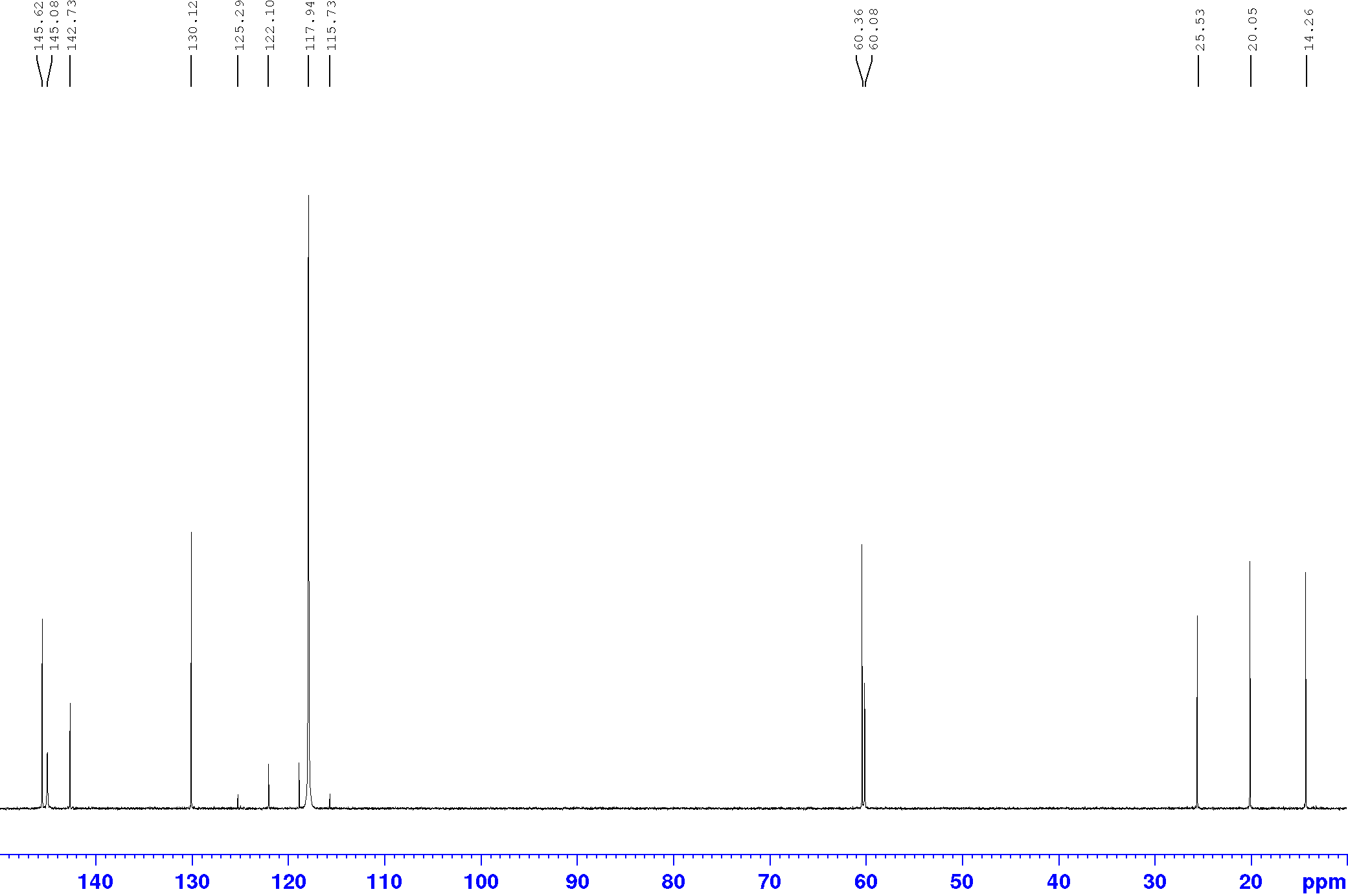
**Figure 44.** ^13^C NMR of 5-ethyl-1-(2-hydroxyethyl)-2-methylpyridinium bis(trifluoromethyl sulfonyl)amide, **12Tf_2_N**

**
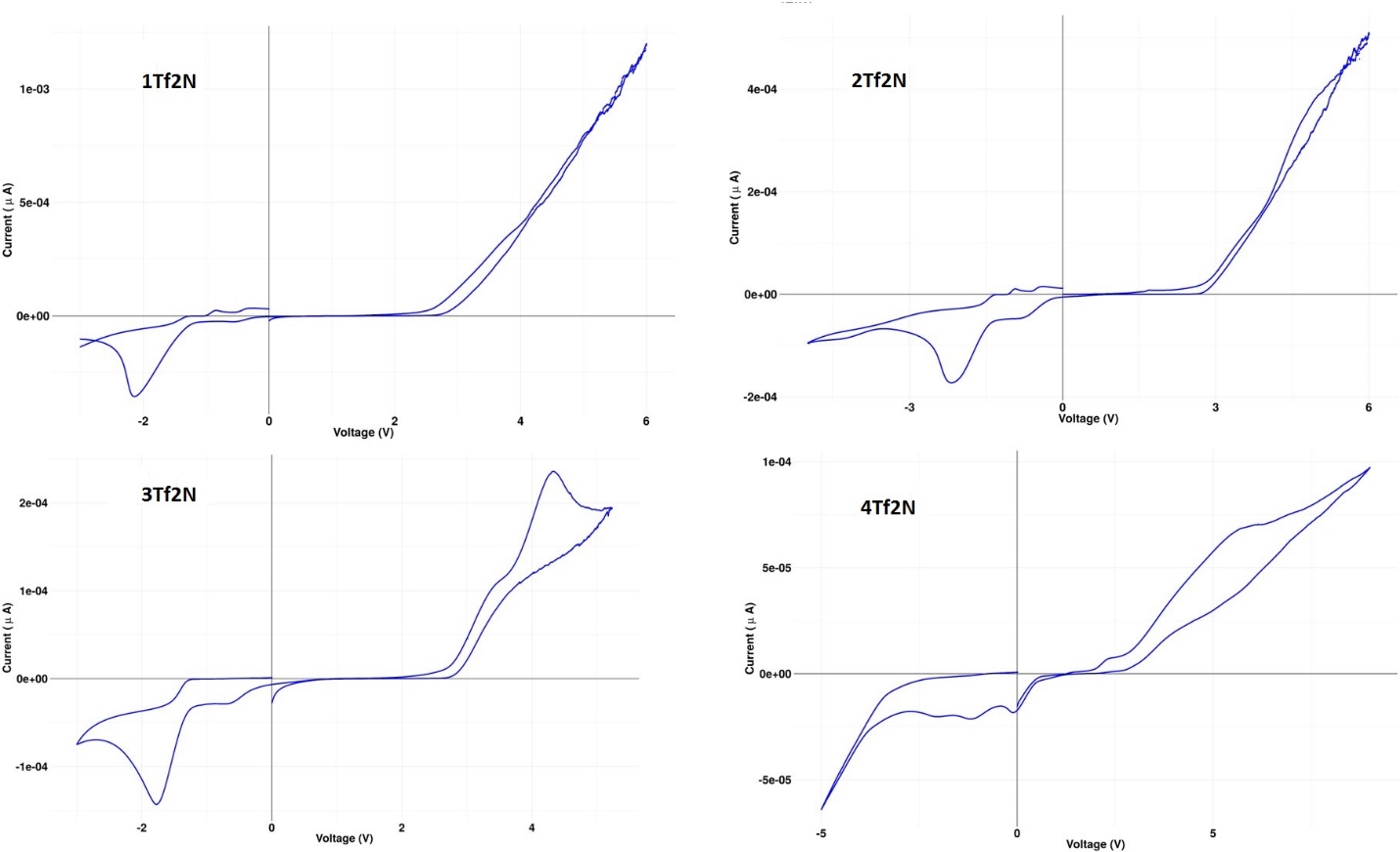
**

**
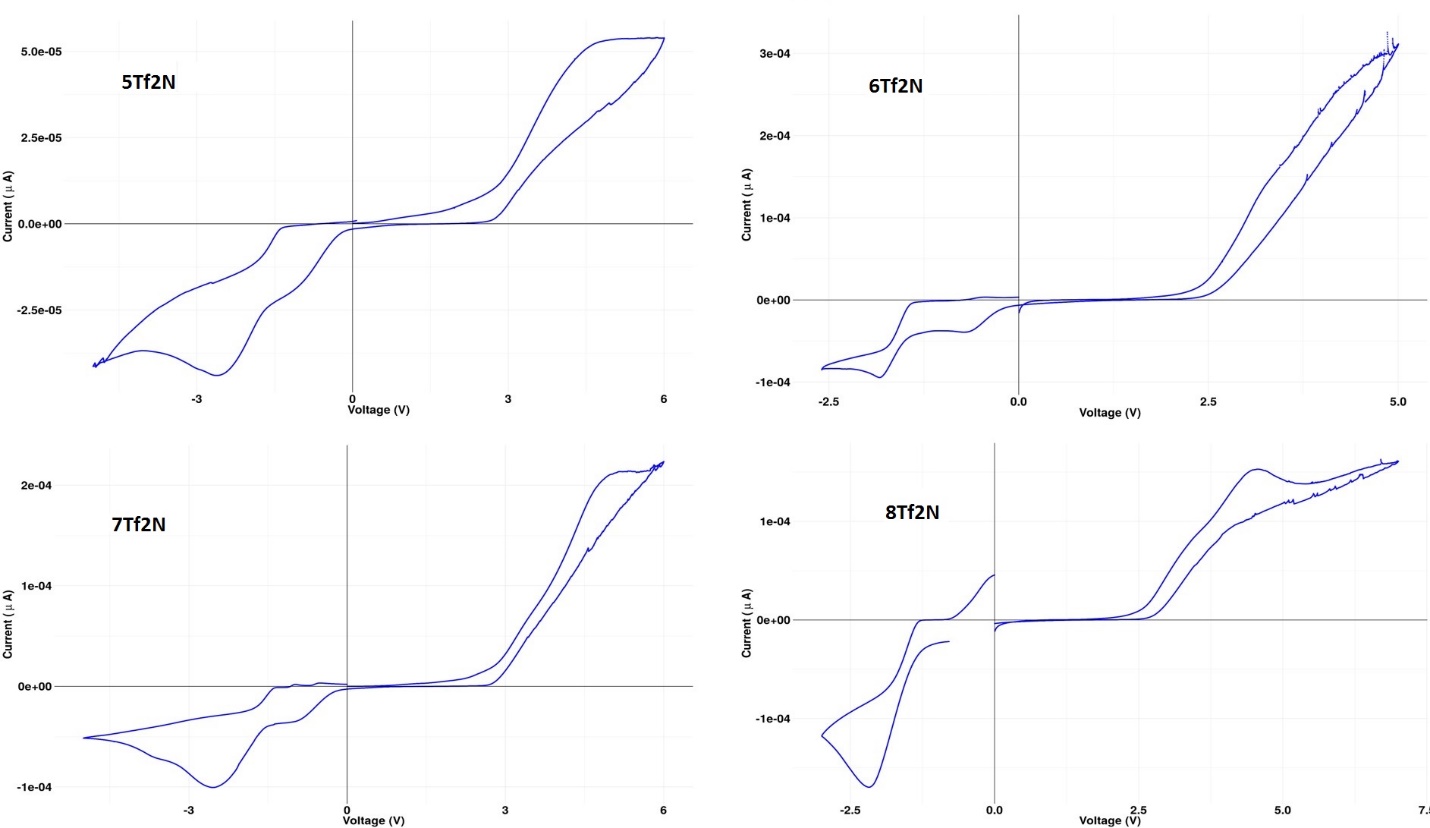
**

**
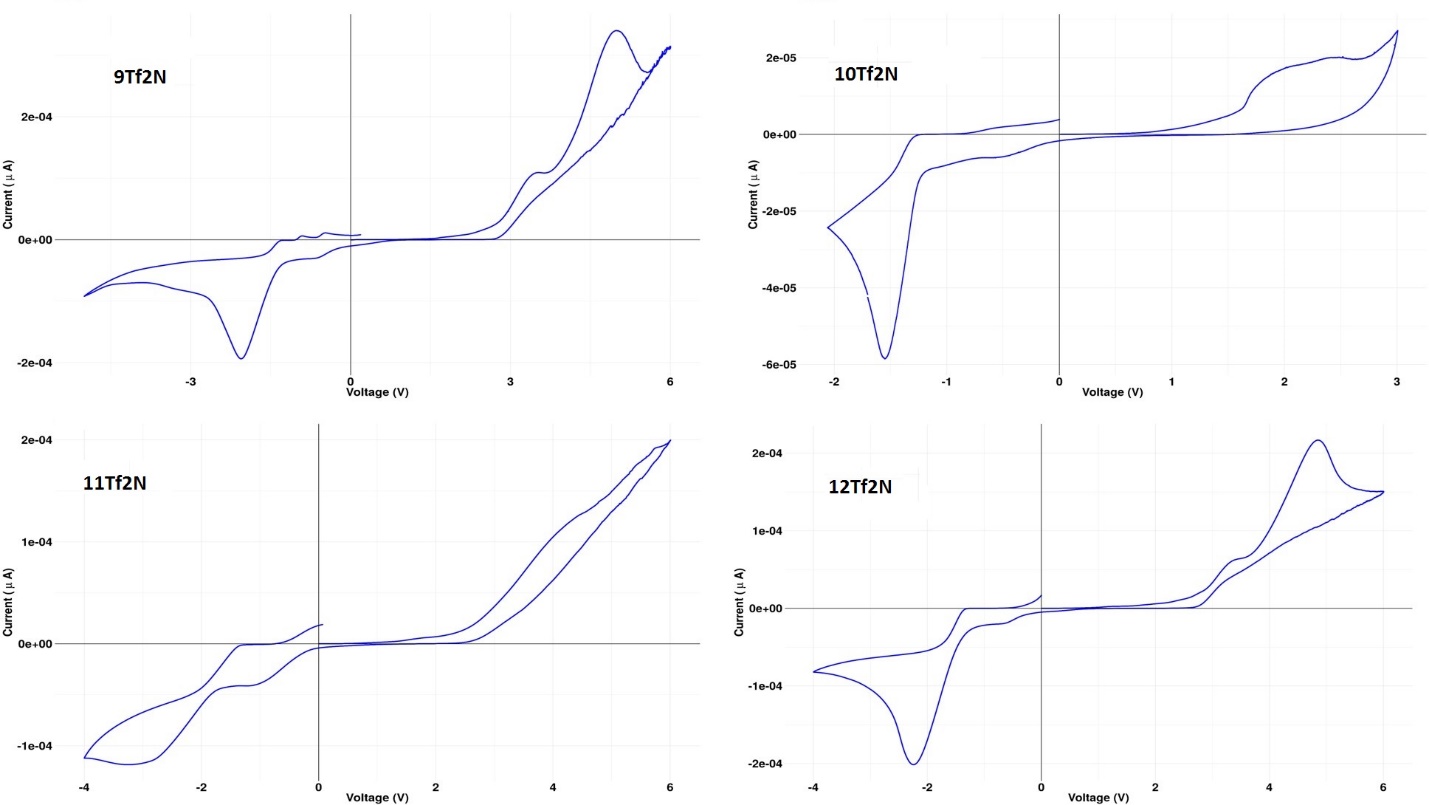
**

**Figure 45.** Cyclic voltammetry of hydroxyl-functionalized pyridinium ILs **1-12Tf_2_N.**

**Table 1.** Thermal decomposition temperature (T_d_) of hydroxyl-functionalized pyridinium ILs **1-12Tf_2_N.**

| **Entry** | **ILs** | **T_d_ [°C]** at 10 wt % loss  **Experimental** | **T_d_ [°C]** at 10 wt % loss  **Predicted** |
| --- | --- | --- | --- |
| 1 | **1 Tf_2_N** | 389 | 371± 56 |
| 2 | **2 Tf_2_N** | 392 | 372 ± 58 |
| 3 | **3Tf_2_N** | 401 | 376 ± 54 |
| 4 | **4 Tf_2_N** | 405 | 377 ± 56 |
| 5 | **5 Tf_2_N** | 377 | 374 ± 57 |
| 6 | **6 Tf_2_N** | 396 | 360 ±60 |
| 7 | **7 Tf_2_N** | 403 | 364 ± 59 |
| 8 | **8 Tf_2_N** | 401 | 372 ± 55 |
| 9 | **9Tf_2_N** | 384 | 369 ± 59 |
| 10 | **10 Tf_2_N** | 401 | 378 ± 53 |
| 11 | **11Tf_2_N** | 401 | 376 ± 56 |
| 12 | **12 Tf_2_N** | 397 | 366 ± 60 |

Experimental standard uncertainties are u(T) = ± 1°C. Predicted uncertainties for the ML models are based on bootstrapping [1].

| **Entry** | **ILs** | **T_d_ [°C] at 10 % wt loss**  **(experimental)** | **T_d_ [°C] at 10 % wt loss**  **(Predicted)** |
| --- | --- | --- | --- |
| 1 | **1Br** | 267 | 224 ± 64 |
| 2 | **2Br** | 261 | 216 ± 54 |
| 3 | **3Br** | 268 | 213 ± 54 |
| 4 | **4Br** | 268 | 222 ± 65 |
| 5 | **5Br** | 234 | 210 ± 57 |
| 6 | **6Br** | 261 | 214 ± 52 |
| 7 | **7Br** | 268 | 229 ± 57 |
| 8 | **8Br** | 269 | 217 ± 53 |
| 9 | **9Br** | 251 | 228 ± 59 |
| 10 | **10Br** | 267 | 218 ± 54 |
| 11 | **11Br** | 259 | 220 ± 66 |
| 12 | **12Br** | 263 | 208 ± 43 |

**Table 2.** Thermal decomposition temperature (T_d_) of hydroxyl-functionalized pyridinium [Br ^-^] salts **1-12Br**

Experimental standard uncertainties are u(T) = ± 1°C. Predicted uncertainties for the ML models are based on bootstrapping [1].

**Table 3.** Experimental density values of Tf_2_N anion based hydroxyl-functionalized ILs **1-12Tf_2_N.**

| T ºC | **Density (***ρ*/g/ cm^3^); Experimental | | | | | | | | | | | |
| --- | --- | --- | --- | --- | --- | --- | --- | --- | --- | --- | --- | --- |
|  | **1Tf_2_N** | **2Tf_2_N** | **3Tf_2_N** | **4Tf_2_N** | **5Tf_2_N** | **6Tf_2_N** | **7Tf_2_N** | **8Tf_2_N** | **9Tf_2_N** | **10Tf_2_N** | **11Tf_2_N** | **12Tf_2_N** |
| 10 | 1.602 | 1.551 | 1.540 | 1.544 | 1.534 | 1.521 | 1.535 | 1.507 | 1.528 | 1.511 | 1.554 | 1.483 |
| 15 | 1.597 | 1.547 | 1.536 | 1.539 | 1.529 | 1.516 | 1.530 | 1.503 | 1.523 | 1.507 | 1.549 | 1.478 |
| 20 | 1.592 | 1.542 | 1.531 | 1.534 | 1.524 | 1.511 | 1.526 | 1.498 | 1.519 | 1.502 | 1.544 | 1.473 |
| 25 | 1.587 | 1.537 | 1.526 | 1.529 | 1.519 | 1.506 | 1.521 | 1.493 | 1.514 | 1.497 | 1.539 | 1.469 |
| 30 | 1.582 | 1.532 | 1.522 | 1.524 | 1.515 | 1.502 | 1.516 | 1.488 | 1.509 | 1.492 | 1.534 | 1.464 |
| 35 | 1.577 | 1.527 | 1.517 | 1.520 | 1.510 | 1.497 | 1.511 | 1.484 | 1.504 | 1.487 | 1.529 | 1.460 |
| 40 | 1.572 | 1.522 | 1.512 | 1.515 | 1.505 | 1.492 | 1.506 | 1.479 | 1.500 | 1.483 | 1.524 | 1.455 |
| 45 | 1.567 | 1.518 | 1.507 | 1.510 | 1.501 | 1.487 | 1.502 | 1.474 | 1.495 | 1.479 | 1.519 | 1.450 |
| 50 | 1.562 | 1.513 | 1.503 | 1.505 | 1.496 | 1.483 | 1.497 | 1.470 | 1.490 | 1.473 | 1.514 | 1.446 |
| 55 | 1.557 | 1.508 | 1.498 | 1.500 | 1.491 | 1.478 | 1.492 | 1.465 | 1.486 | 1.469 | 1.509 | 1.441 |
| 60 | 1.552 | 1.504 | 1.493 | 1.496 | 1.487 | 1.474 | 1.487 | 1.460 | 1.481 | 1.464 | 1.499 | 1.437 |
| 65 | 1.548 | 1.499 | 1.489 | 1.491 | 1.482 | 1.469 | 1.483 | 1.456 | 1.476 | 1.460 | 1.494 | 1.433 |
| 70 | 1.543 | 1.494 | 1.484 | 1.486 | 1.478 | 1.464 | 1.478 | 1.451 | 1.472 | 1.455 | 1.489 | 1.428 |
| 75 | 1.538 | 1.490 | 1.480 | 1.482 | 1.473 | 1.460 | 1.473 | 1.447 | 1.467 | 1.450 | 1.484 | 1.424 |
| 80 | 1.533 | 1.485 | 1.475 | 1.477 | 1.469 | 1.455 | 1.469 | 1.442 | 1.463 | 1.446 | 1.479 | 1.419 |

Experimental standard uncertainties are u(ρ) = ± 0.00001 g·cm−3 and u(T) = ± 0.01 K

**Table 4.** Predicted density values of Tf_2_N anion based hydroxyl-functionalized ILs **1-12Tf_2_N.**

| T ºC | **Density (***ρ*/g/ cm^3^); Predicted | | | | | | | | | | | |
| --- | --- | --- | --- | --- | --- | --- | --- | --- | --- | --- | --- | --- |
|  | **1Tf_2_N** | **2Tf_2_N** | **3Tf_2_N** | **4Tf_2_N** | **5Tf_2_N** | **6Tf_2_N** | **7Tf_2_N** | **8Tf_2_N** | **9Tf_2_N** | **10Tf_2_N** | **11Tf_2_N** | **12Tf_2_N** |
| 10 | 1.583 | 1.549 | 1.545 | 1.535 | 1.508 | 1.502 | 1.507 | 1.496 | 1.501 | 1.492 | 1.511 | 1.463 |
| 15 | 1.578 | 1.544 | 1.530 | 1.531 | 1.503 | 1.497 | 1.503 | 1.491 | 1.496 | 1.487 | 1.507 | 1.458 |
| 20 | 1.573 | 1.539 | 1.535 | 1.526 | 1.498 | 1.492 | 1.498 | 1.486 | 1.491 | 1.482 | 1.502 | 1.453 |
| 25 | 1.568 | 1.534 | 1.530 | 1.521 | 1.493 | 1.487 | 1.493 | 1.481 | 1.486 | 1.478 | 1.497 | 1.448 |
| 30 | 1.563 | 1.529 | 1.525 | 1.516 | 1.488 | 1.482 | 1.488 | 1.476 | 1.481 | 1.473 | 1.492 | 1.443 |
| 35 | 1.558 | 1.524 | 1.520 | 1.511 | 1.483 | 1.477 | 1.483 | 1.471 | 1.476 | 1.468 | 1-488 | 1.439 |
| 40 | 1.553 | 1.520 | 1.515 | 1.506 | 1.479 | 1.472 | 1.478 | 1.467 | 1.471 | 1.463 | 1.483 | 1.434 |
| 45 | 1.548 | 1.515 | 1.511 | 1.501 | 1.474 | 1.468 | 1.473 | 1.462 | 1.467 | 1.458 | 1.478 | 1.429 |
| 50 | 1.543 | 1.510 | 1.506 | 1.496 | 1.469 | 1.463 | 1.468 | 1.457 | 1.462 | 1.454 | 1.474 | 1.424 |
| 55 | 1.538 | 1.505 | 1.501 | 1.491 | 1.464 | 1.458 | 1.464 | 1.452 | 1.457 | 1.449 | 1.469 | 1.420 |
| 60 | 1.534 | 1.500 | 1.496 | 1.487 | 1.459 | 1.453 | 1.459 | 1.448 | 1.452 | 1.444 | 1.464 | 1.415 |
| 65 | 1.529 | 1.495 | 1.491 | 1.482 | 1.455 | 1.449 | 1.454 | 1.443 | 1.448 | 1.439 | 1.460 | 1.410 |
| 70 | 1.524 | 1.490 | 1.486 | 1.477 | 1.450 | 1.444 | 1.449 | 1.438 | 1.443 | 1.435 | 1.455 | 1.406 |
| 75 | 1.519 | 1.486 | 1.482 | 1.472 | 1.445 | 1.439 | 1.445 | 1.433 | 1.438 | 1.430 | 1.451 | 1.401 |
| 80 | 1.514 | 1.481 | 1.477 | 1.467 | 1.440 | 1.434 | 1.440 | 1.429 | 1.433 | 1.425 | 1.446 | 1.396 |

**Table 5.** Experimental viscosity values of Tf_2_N anion based hydroxyl-functionalized ILs **1-12Tf_2_N.**

| T ºC | **Viscosity (***η*/mPa.s) | | | | | | | | | | | |
| --- | --- | --- | --- | --- | --- | --- | --- | --- | --- | --- | --- | --- |
|  | **1Tf_2_N** | **2Tf_2_N** | **3Tf_2_N** | **4Tf_2_N** | **5Tf_2_N** | **6Tf_2_N** | **7Tf_2_N** | **8Tf_2_N** | **9Tf_2_N** | **10Tf_2_N** | **11Tf_2_N** | **12Tf_2_N** |
| 20 | 143.1 | 127.1 | 112.2 | 209.7 | 304.1 | 225.1 | 217.1 | 199.1 | 141.7 | 116.6 | 216.60 | 208.5 |
| 30 | 80.91 | 78.64 | 66.41 | 124.6 | 170.7 | 120.8 | 78.6 | 104.0 | 80.6 | 64.0 | 115.20 | 109.6 |
| 40 | 50.14 | 49.33 | 40.87 | 72.41 | 97.2 | 72.98 | 49.3 | 62.5 | 50.58 | 43.2 | 71.50 | 67.0 |
| 50 | 32.32 | 33.22 | 28.42 | 47.32 | 64.1 | 47.15 | 33.2 | 39.2 | 39.90 | 29.5 | 46.50 | 42.6 |
| 60 | 21.59 | 23.59 | 21.81 | 31.14 | 43.3 | 30.0 | 23.5 | 25.7 | 24.10 | 24.2 | 33.20 | 29.3 |
| 70 | 16.55 | 17.51 | 16.84 | 22.15 | 28.7 | 22.7 | 14.7 | 18.1 | 17.80 | 17.9 | 24.00 | 22.0 |
| 80 | 12.78 | 13.49 | 12.15 | 17.04 | 21.6 | 17.0 | 11.5 | 13.8 | 10.91 | 14.4 | 16.30 | 15.8 |

Experimental standard uncertainties are u (η) = ± 0.32 % mPa·s and u (T) = ± 0.01 K.

| T ºC | **Viscosity (***η*/mPa.s) | | | | | | | | | | | |
| --- | --- | --- | --- | --- | --- | --- | --- | --- | --- | --- | --- | --- |
|  | **1Tf_2_N** | **2Tf_2_N** | **3Tf_2_N** | **4Tf_2_N** | **5Tf_2_N** | **6Tf_2_N** | **7Tf_2_N** | **8Tf_2_N** | **9Tf_2_N** | **10Tf_2_N** | **11Tf_2_N** | **12Tf_2_N** |
| 20 | 108.1 | 74.14 | 76.93 | 103.68 | 75.38 | 73.86 | 94.81 | 98.60 | 82.49 | 92.60 | 117.80 | 83.97 |
| 30 | 65.83 | 46.24 | 47.92 | 63.81 | 47.35 | 46.46 | 58.97 | 61.28 | 51.65 | 57.72 | 72.68 | 52.91 |
| 40 | 42.15 | 30.25 | 31.31 | 41.24 | 31.17 | 30.62 | 38.48 | 39.96 | 33.90 | 37.74 | 47.09 | 34.93 |
| 50 | 28.19 | 20.63 | 21.33 | 27.83 | 21.38 | 21.03 | 26.19 | 27.18 | 23.19 | 25.73 | 31.84 | 24.02 |
| 60 | 19.60 | 14.60 | 15.08 | 19.49 | 15.21 | 14.97 | 18.49 | 19.18 | 16.45 | 18.20 | 22.35 | 17.12 |
| 70 | 14.09 | 10.66 | 11.01 | 14.12 | 11.16 | 11.00 | 13.49 | 13.98 | 12.05 | 13.29 | 16.21 | 12.60 |
| 80 | 10.44 | 8.02 | 8.27 | 10.53 | 8.43 | 8.31 | 10.12 | 10.49 | 9.08 | 9.99 | 12.11 | 9.53 |

**Table 6.** Predicted viscosity values of Tf_2_N anion based hydroxyl-functionalized ILs **1-12Tf_2_N.**

[1] V. Venkatraman, B.K. Alsberg, Quantitative structure-property relationship modelling of thermal decomposition temperatures of ionic liquids, J. Mol. Liq., 223 (2016) 60-67.
